# Renal tubular epithelial cell related partial epithelial-mesenchymal transition in AAⅠ induced renal fibrosis via Wnt7b/β-catenin signaling

**DOI:** 10.3389/fphar.2025.1571960

**Published:** 2025-05-13

**Authors:** Yi-Fan Wang, Dan Zheng, Ying Zhang, Xiao-Fen Li, Ming Xia, Hai-Ming Tang, Chun-Hua Huang, Mao-Juan Li, Di-Dong Lou

**Affiliations:** ^1^ Judicial Appraisal Center, Guizhou University of Traditional Chinese Medicine, Guiyang, Guizhou, China; ^2^ Key Laboratory of Forensic Toxicology of Herbal Medicines, Guizhou Education Department, Guiyang, Guizhou, China; ^3^ Guiyang Maternal and Child Healthcare Hospital, Guiyang, Guizhou, China

**Keywords:** aristolochic acid nephropathy, renal tubular epithelial cell, Wnt7b/β-catenin, epithelial-mesenchymal transition, damage and repair

## Abstract

**Introduction:**

This study investigates the pathological progressions in kidneys affected by aristolochic acid nephropathy (AAN) and explores the molecular mechanisms underlying the fibrotic process, specifically focusing on the Wnt7b/β-catenin signaling pathway.

**Methods:**

Both mice and human kidney-2 (HK-2) cells were treated with aristolochic acid I (AAI). In mice, we monitored blood urea nitrogen (BUN), serum creatinine (Scr), kidney injury molecule-1 (KIM-1), pathological modifications of renal tubular epithelial cells (RTECs), and fibrosis degrees during acute/chronic disease phases. Wnt7b/β-catenin expression was evaluated through transcriptome analysis and laboratory assays (immunohistochemistry, Western blotting, immunoelectron microscopy) in acute AAN and cultured cells. Concurrent assays measured representative proteins: Aquaporin 1 (AQP1), Topoisomerase IIα (TOP2A), Vascular Cell Adhesion Molecule-1 (VCAM-1), and α-smooth muscle actin (α-SMA) in chronic AAN RTECs.

**Results:**

AAI increased Scr, BUN, and KIM-1 levels by causing RTEC necrotic shedding in acute stages and promoted renal interstitial fibrosis chronically. Elevated Wnt7b pathway proteins enhanced damaged RTEC regeneration, with regenerated cells expressing mesenchymal proteins VCAM-1 and α-SMA.

**Discussion:**

The Wnt7b/β-catenin signaling pathway connects acute tubule damage to fibrosis, explaining AAN’s pathological continuum. These findings clarify how acute injury progresses to chronic fibrosis in AAN.

## 1 Introduction

Aristolochic acid Ⅰ, a polycyclic aromatic organic compound, is one of the principal active constituents in the Aristolochiaceae Juss (Wang et al., 2023). It can be potentially ingested either through remedies of specific herbal plants such as *Aristolochia debilis* Siebold & Zucc., *Isotrema manshuriense* (Kom.) H. Huber, and *Isotrema fangchi* in traditional Chinese medicine (TCM), or due to the consumption of AAⅠ-contaminated food or water (Kiliś-Pstrusińska and Wiela-Hojeńska, 2021; Kobets et al., 2022). For example, in the Balkan Peninsula of Europe, there have been many cases where grains were mixed with *Aristolochia clematitis* (Drăghia et al., 2021). Numerous studies have confirmed that these herbal preparations or AAⅠ-containing pollutants can induce AAN, with the main pathological manifestations being damage to RTECs and the development of renal interstitial fibrosis (Zhou et al., 2023). The progression from acute AAN to chronic AAN is a continuous and dynamic pathological process. Renal fibrosis is the final outcome, inevitably leading to chronic kidney disease (CKD) (Fu et al., 2018). Subsequently, renal failure becomes an inevitable fate, forcing patients to rely on kidney dialysis for survival or to turn to kidney transplantation (Lukinich-Gruia et al., 2022).

Aristolochic acid Ⅰ is absorbed into the bloodstream through the gastrointestinal tract and then binds to plasma albumin. Subsequently, it undergoes biochemical conversion and is excreted via membrane transport channels of RTECs (Pomyalov et al., 2024). The key factor involved in this excretion process is the organic anion transporter (OAT). OATs, which play a crucial role in the active or passive transport of various organic anions, such as endogenous metabolites, drugs, and their metabolites, are mainly located on the plasma membrane infoldings of RTECs (Li et al., 2020). They exhibit strong affinity for AAⅠ and mainly function to transport AAⅠ from the blood into RTECs for excretion, thereby forming AAⅠ-DNA adducts, ultimately inducing RTEC damage (Xue et al., 2011).

In this study, we aimed to explore relevant molecular events, as well as the pathological morphology and function of epithelial cells in the kidneys of mouse models of acute and chronic AAN. Specifically, we focused on the injury, regeneration, repair process of EMT, and renal interstitial fibrosis. The classic EMT signaling pathway, namely, the Wnt/β-catenin signaling pathway (Xue et al., 2024), was investigated to analyze the molecular mechanism underlying the initiation of damaged epithelial cell regeneration in acute AAN. The Wnt/β-catenin pathway, evolutionarily conserved across species, remains largely dormant in healthy adult tissues but reactivates during organ injury and repair (Schunk et al., 2021). Earlier studies have shown that Wnt7b, a protein released by renal medullary stem cells, helps develop the renal medulla and collecting ducts (Yu et al., 2009). It also helps maintain the integrity of PCTECs during repair (Schunk et al., 2021). Because of this, Wnt7b might serve as a potential prognostic biomarker for patients with AAN (Li et al., 2024). These findings have led to further investigations into the role of Wnt7b in both tubular repair and fibrotic remodeling. Moreover, the expression levels of AQP1 for epithelial characteristics, which is a water channel protein of renal tubular epithelial cells for facilitating fluid transport (Su et al., 2020), TOP2A for the characteristics of cell proliferation, which is a representative protein participating in DNA replication (Uusküla-Reimand and Wilson, 2022), VCAM-1 for mesenchymal adhesion characteristics, which is a representative protein assisting cell adhesion (Melchinger et al., 2024), and α-SMA for mesenchymal characteristics (Vierhout et al., 2021) were assessed. These analyses were designed to understand how the functions of EMT cells are connected to the activity of myofibroblasts in chronic AAN., and reveal how dysregulated repair mechanisms drive fibrotic progression through the sustained Wnt/β-catenin activation.

In conclusion, this project delved deeply into the molecular mechanism behind the damage and repair process of epithelial cells and its relation to interstitial fibrosis. By closely examining the acute and chronic pathological features in kidney tubular diseases caused by AAⅠ, this work is beneficial for further elucidating the cause of AAN, devising ways to prevent AAN, and providing basic information for treating AAN as well as for public health and safety.

## 2 Material and methods

### 2.1 Chemicals, antibodies and reagents

AAⅠ with a purity of 96% was purchased from Sichuan Vichy Biological Co., Ltd. (Sichuan, China). Dimethyl sulfoxide (DMSO), hematoxylin and eosin (H&E) staining solution, Cell Counting Kit-8 (CCK-8), and RIPA buffer were obtained from Beijing Solarbio Technology Co., Ltd. (Beijing, China). Sodium pentobarbital was purchased from Sigma-Aldrich Corporation (St. Louis, Missouri, United States). Carboxymethylcellulose sodium (CMC-Na) was purchased from Shanghai Zhangyun Chemical Co., Ltd. (Shanghai, China). Cisplatin was sourced from MedChemExpress (New Jersey, United States). Immunohistochemistry (IHC) SP kits were acquired from Beijing Zhongshan Jinqiao Biotechnology Co., Ltd. (Beijing, China). Dulbecco’s Modified Eagle Medium (DMEM) cell culture medium and fetal bovine serum (FBS) were purchased from Procell Life Science & Technology Co., Ltd. (Wuhan, China).

Rabbit polyclonal antibody against β-actin (WG3329146), Wnt7b (35C9BA18), α-SMA (XC3528314F), and Aquaporin 1 (AQP1, 35C9CA08) were purchased from Thermo Fisher Scientific (Massachusetts, United States). Rabbit polyclonal antibody against KIM-1 (AD110627), VCAM-1 (BB08102994), and TOP2A (BA09132893) were purchased from Bioss Biotechnology (Beijing, China). Rabbit polyclonal antibodies against β-catenin (00100092) and MMP7 (00101692) were obtained from Wuhan Sanying Biotechnology Co., Ltd. (Wuhan, China).

### 2.2 Animals

A total of 111 male Kunming mice, weighing 32–37 g and aged 8 weeks, were supplied by Changsha Tianqin Technology Co., Ltd. (Changsha, China). The mice were housed in a controlled environment where the temperature was maintained at 25°C ± 2°C, the humidity at 65% ± 5%. They were fed a standard pellet diet and had free access to water, and a 12-h light/dark cycle was implemented. The animals were allowed to acclimate for 1 week prior to the commencement of the experiment. All experimental procedures involving animals were carried out in strict accordance with the Guide for the Care and Use of Laboratory Animals published by the US National Institutes of Health. The animal study protocols were reviewed and approved by the ethics committee of Guizhou University of Traditional Chinese Medicine (Guizhou, China), with the ethics approval number 20220106.

Survival Curves: A solution of 2 mg/mL AAⅠ was prepared by dissolving 200 mg AAⅠ in 10 mL DMSO, followed by dilution with 0.4% CMC-Na solution. Mice were intragastrically administered with 5–20 mg/kg/day of AAⅠ or 6 mL/kg/day of 0.4% CMC-Na (n = 15). The number of surviving mice in each group was counted daily for 15 days. The log-rank test was employed to analyze the differences in survival rates among the groups.

The remaining mice were randomly sorted into six groups (n = 6): namely, the AAN-Con group, to which an equivalent volume of 0.4% CMC-Na was administered; the AAN-2d group, where AAⅠ was provided on the initial day; the AAN-4d group, in which AAⅠ was given twice with a 2-day interval between administrations; the AAN-6d group, with AAⅠ administered 3 times at 2-day intervals; the AAN-8d group, where AAⅠ was given 4 times at 2-day intervals; and the AAN-4w group, in which AAⅠ was administered 14 times at 2-day intervals. AAⅠ was dissolved in CMC-Na, and the intragastric gavage dose for each group was 5 mg/kg/d. The cumulative AAⅠ doses were established in accordance with the survival curve of the mice. Daily surveillance was carried out to identify any abnormal manifestations in all the mice throughout the study period.

After a 2-day interval following the final administration of AAⅠ, the mice were anesthetized by intraperitoneal injection of sodium pentobarbital at a dosage of 50 mg/kg. Blood samples were collected by retro-orbital puncture using heparinized capillary tubes. Thereafter, the mice were euthanized via cervical dislocation. The bilateral renal tissues were rinsed with PBS. Following tissue collection, the left kidney was fixed in 4% paraformaldehyde (PFA) for 24 h at 4°C for subsequent histological analysis. The right kidney was divided into two portions: three-quarters were homogenized in RIPA lysis buffer (containing 1 mM PMSF protease inhibitor) for Western blot analysis, while the remaining one-quarter from the AAN-4w and AAN-Con groups was immediately snap-frozen in liquid nitrogen and stored at −80°C for transcriptomic profiling.

### 2.3 The blood biochemical analysis

Blood samples were centrifuged at 3,000 × g for 10 min. Subsequently, the supernatant was carefully transferred to a new blood collection tube. The levels of BUN and Scr were quantified using an automated biochemistry analyzer (Mindray, Shenzhen, China).

### 2.4 Transcriptome analysis

Total RNA was extracted from kidney tissues of AAN-4w and AAN-Con groups using Trizol reagent. Sequencing libraries were prepared and sequenced on the BGI DNBSEQ-T7 (Wuhan Baiyihuineng Biotechnology Co., Ltd.). Raw data were filtered with FastQC software. The gene expression levels of each sample were quantitatively analyzed using the Kallisto software. *P* values were adjusted by the Benjamini–Hochberg (B&H) method. The screening threshold for significant differentially expressed genes (DEGs) was set as: adjusted *p* value ≤ 0.05 and |log2fold change| ≥ 2. Heatmap visualization of DEG expression patterns (z-score normalization) and bubble plots (enrichment analysis) were generated using the online tools of the Beijing Bioinformatics Center (BIC) platform. The bubble plots highlight key pathway gene sets, with color intensity representing the significance level [-log10(p-value)] and bubble size corresponding to the number of enriched genes.

### 2.5 Histopathological and immunohistochemical analysis

Renal tissues were fixed in 4% paraformaldehyde for 24 h, paraffin-embedded, and sectioned into 3-μm slices using a rotary microtome. Serial sections underwent hematoxylin and eosin (HE) staining and Masson’s trichrome staining using aniline blue for collagen visualization.

For immunohistochemistry (IHC), deparaffinized sections were subjected to antigen retrieval in citrate buffer (pH 6.0) at 95°C for 20 min, followed by endogenous peroxidase blockade with 3% H_2_O_2_ for 15 min and protein blocking with 5% BSA for 30 min. Sections were incubated overnight at 4°C with primary antibodies: Wnt7b (diluted 1:200), β-catenin (1:200), MMP7 (1:100), AQP1 (1:200), VCAM-1 (1:300), TOP2A (1:200), and α-SMA (1:200), Sections were then probed with HRP-conjugated secondary antibody (1:500) for 1 h at room temperature. DAB chromogenic reaction and hematoxylin counterstaining were performed prior to microscopic observation using a CX33 microscope (Leica Camera AG, Germany).

In the HE staining, tubules with more than 4/5 of the basement membrane covered by epithelial cells were classified as structurally normal tubules. The tubular injury indices (the ratio of pathological to structural changes) were calculated to evaluate the progression of the lesions. Masson-stained and IHC images were quantified following Prajakta’s method to calculate the average optical density via the random area counting method, as described by Deshpande (Deshpande et al., 2021).

### 2.6 Cell culture and treatment

HK-2 cells were maintained in low-glucose DMEM supplemented with 10% fetal bovine serum (FBS) under standard culture conditions (37°C, 5% CO_2_). Cells were passaged at 75%–80% confluence using 0.25% trypsin-EDTA and seeded into either 6-well plates (2.5 × 10^4^ cells/well) for morphological observation and protein extraction or 96-well plates (6 × 10^3^ cells/well) for viability assessment based on experimental requirements. After adherence, cultures were exposed to freshly prepared medium containing AAⅠ (25, 50, 100, 200 μM) or cisplatin (20 μM positive control) for 24 h. The AAⅠ stock solution (15 mM in DMSO) was stored at −20°C and diluted serially with complete medium immediately before exposure to achieve target concentrations. All treatments were maintained for 24 h prior to downstream analyses.

Treated cells in 96-well plates were incubated with 10 μL of CCK-8 reagent per well after treatment. Following 1 h incubation at 37°C, absorbance was measured at 450 nm using a microplate reader.

### 2.7 Western blot

Protein extraction was performed by aspirating treatment media from cultured cells, followed by ice-cold rinsing with PBS. Cellular proteins from both renal tissues and HK-2 cells were lysed using RIPA buffer containing 1 mM PMSF protease inhibitor. Lysates were centrifuged at 12,000 × g for 15 min at 4°C to collect supernatants. Protein samples (30 μg/lane) were separated via 10% SDS-PAGE at 15 V/cm for 90 min, then transferred to PVDF membranes using semi-dry electrophoresis. Membranes were blocked with 5% non-fat milk for 1 h at room temperature prior to overnight incubation.

The PVDF membranes were then incubated overnight at 4°C with primary antibodies, anti-AQP1 (1:800), anti-β-actin (1:5,000), anti-Wnt7b (1:500), anti-β-catenin (1:1,000), and anti-MMP7 (1:1,000). After thorough washing, the membranes were incubated with a goat anti-rabbit secondary antibody (1:5,000) at room temperature for 1 h. Protein bands were visualized using an ECL kit and photographed with a ChemiDoc imaging system (Biorad, United States). Quantitative densitometric analysis of band intensity was performed using ImageJ software, with experimental group data normalized to corresponding controls.

### 2.8 Statistical analysis

All data were processed using GraphPad Prism version 8.0 and expressed as mean ± standard deviation (SD). Normality was assessed using the Shapiro-Wilk test. For data conforming to normal distribution, intergroup comparisons were performed using one-way ANOVA followed by Dunnett’s *post hoc* test for multiple comparisons against a single control group. Statistical significance was defined as *p* < 0.05.

## 3 Results

### 3.1 The renal pathological manifestations of mice in acute AAN

**FIGURE 1:**
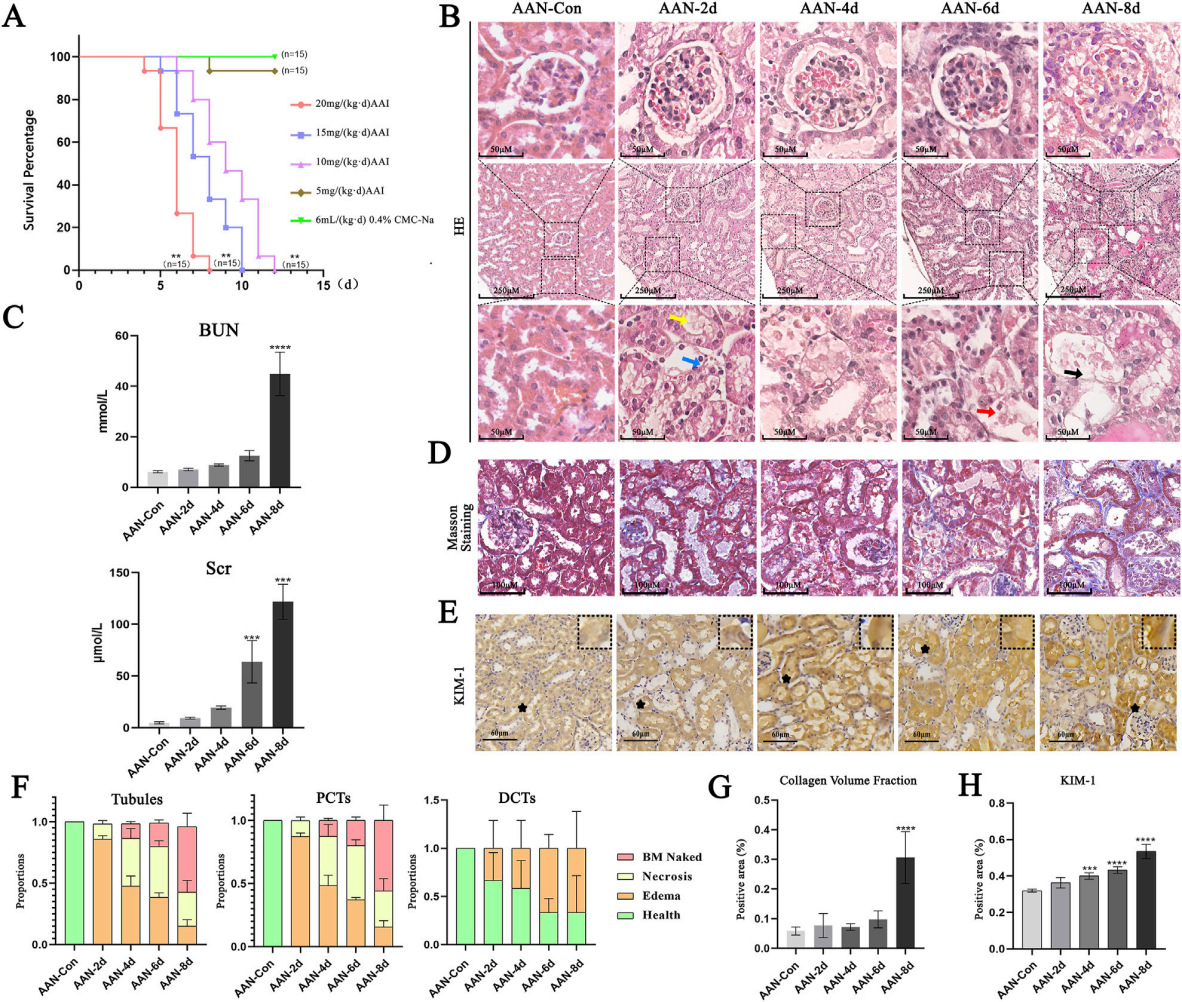
Renal pathological manifestations in acute AAN. **(A)** Survival curves. **(B)** Representative images of HE staining. Yellow arrows point to the rupture of RTEC villi. Blue arrows indicate cell edema. Red arrows show cell necrosis. Black arrows mark the naked basement membrane (BM naked), which is formed following the necrosis and shedding of epithelial cells. **(C)** Levels of BUN and Scr. **(D)** Representative images of Masson staining. **(E)** Representative images of KIM-1. **(F)** Injured Proportions of total tubules, PCT, and DCT. **(G)** Analysis of renal interstitial fibrosis. **(H)** Analysis of KIM-1 positive areas. Notes: Data were presented as mean ± SD with at least three independent experiments. **p < 0.01, ***p < 0.005, ****p < 0.001 vs. AAN-Con.

The survival rate of mice declined significantly with the increase in AAⅠ dosage. All mice died within 8 days at a dosage of 20 mg/kg/day of AAⅠ, whereas most survived at 5 mg/kg/day (Figure 1A). Markedly elevated levels of BUN and Scr were detected in mice exposed to AAⅠ, suggesting impaired renal function (Figure 1C). The toxic effect of AAⅠ on renal tubules, rather than other renal structures, was more prominent, and the degree of injury escalated gradually with induction time. From 2 to 8 days after AAⅠ induction, villi rupture, edema, necrosis, and detachment from the basement membrane were successively observed in epithelial cells of proximal convoluted tubule (PCT). Renal tubular casts were present at the corticomedullary junction (CMJ) from day 6 to day 8. Meanwhile, on day 8, minor edema was exhibited in the distal convoluted tubule (DCT) and collecting tubule (CT). However, no obvious pathological changes were noted in the renal glomerulus (Figures 1B,F). Masson staining demonstrated that AAⅠ enhanced collagen fiber secretion and tubulointerstitial fibrosis (Figures 1D,G). The level of KIM-1 was also elevated in the renal tissues of AAN (Figures 1E,H).

### 3.2 Analysis of the transcriptome database in mice

**FIGURE 2:**
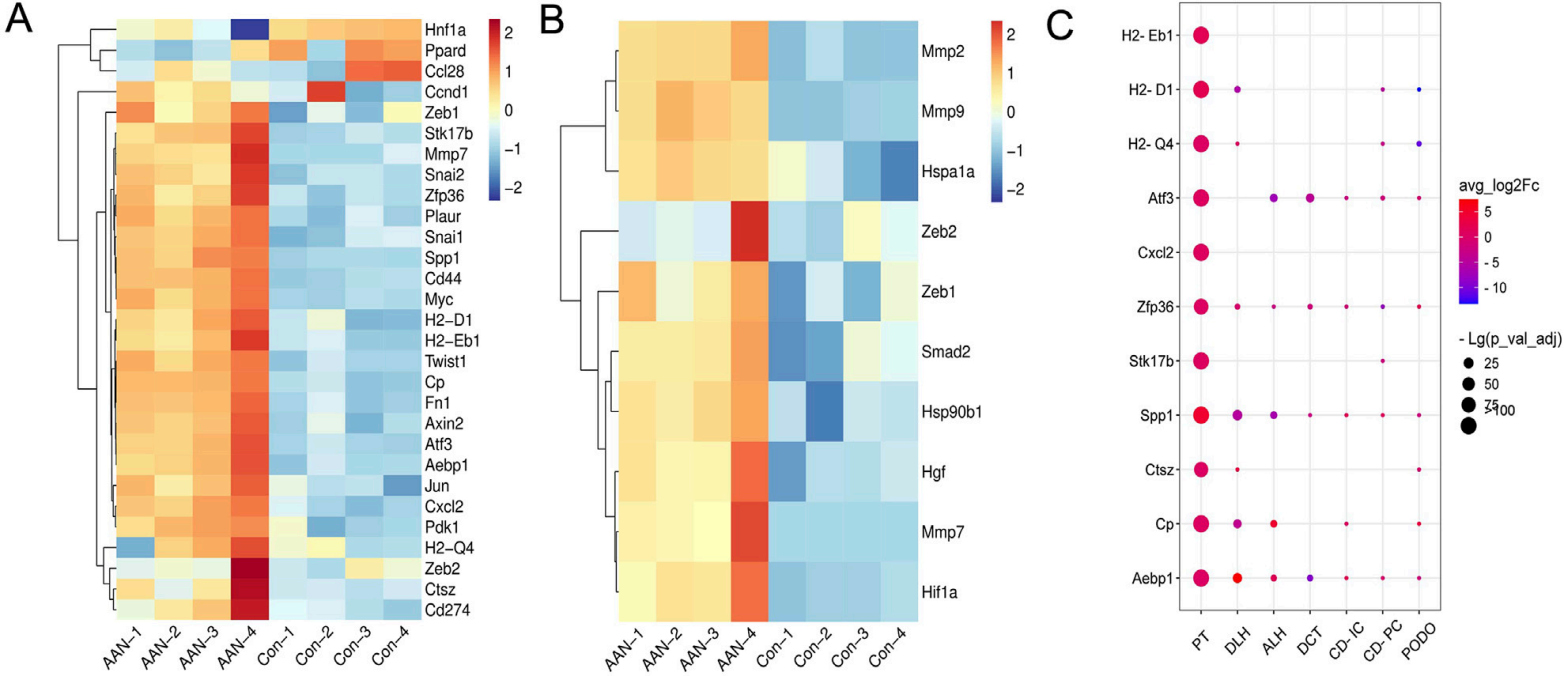
Analysis of the transcriptome database in AAN. **(A)** Expression levels of genes related to the Wnt signaling pathway. **(B)** Expression levels of EMT related genes. **(C)** Based on single-cell sequencing data from Chen (Chen et al., 2022), the levels of Wnt signaling pathway-related genes was increased in the proximal tubule cells.

In comparison with the control group, the AAN-4w group demonstrated significant upregulation of Wnt/β-catenin signaling-associated genes, namely, CD44, Snail2, and Axin2 (Figure 2A). Concurrently, the expression levels of genes related to the EMT process, such as MMP7, Zeb1, and Hgf, were also increased (Figure 2B). Re-analyzing the data of the single-cell sequencing results of acute AAN by Chen (Chen et al., 2022), it was found that the upregulation of genes related to the Wnt signaling pathway in proximal tubule cells was particularly noticeable (Figure 2C).

### 3.3 The levels of Wnt7b, β-catenin and MMP7 proteins in the kidney of mice

**FIGURE 3:**
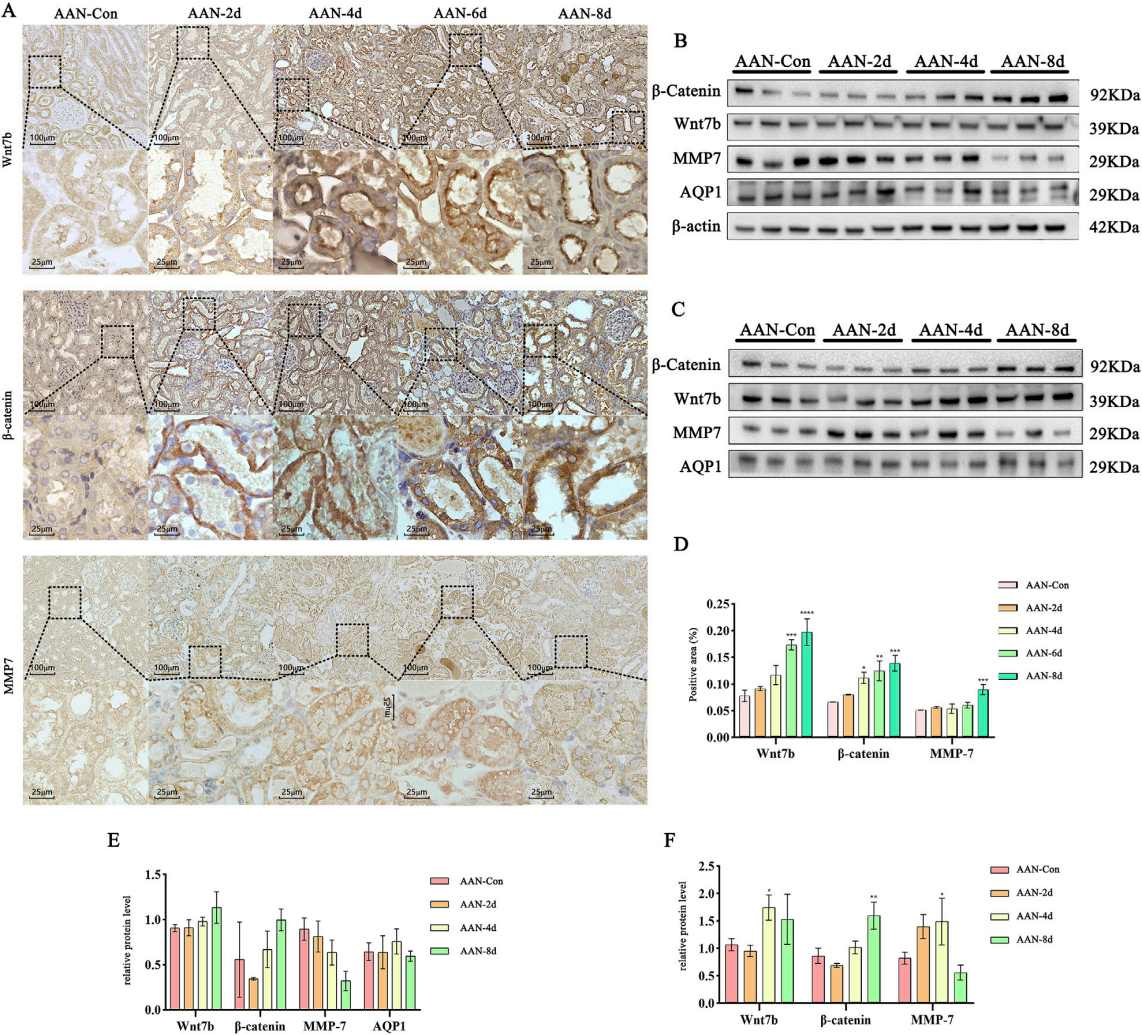
Expression Profiles of Wnt7b, β-catenin, and MMP7 Proteins. **(A)** Representative images obtained from IHC staining. **(B,C)** WB images with β-actin and AQP1 as references, respectively. **(D)** OD analysis of proteins in IHC. **(E,F)** Quantitative analysis of proteins with β-actin and AQP1 as references, respectively. Data are presented as mean ± SD based on at least three independent experiments. *p < 0.05, **p < 0.01, ***p < 0.005, ****p < 0.001 vs. AAN-Con.

IHC optical densities were elevated compared to the control, correlating positively with the AAⅠ treatment time. Wnt7b began to accumulate in the villi of proximal convoluted tubule epithelial cells (PCTECs) from day 2 and reached its peak on day 8. Similarly, the expression of β-catenin and MMP7 was basically synchronous with that of Wnt7b and were upregulated. In addition, β-catenin seemed to be expressed in the cytoplasm and nucleus, while MMP7 was clustered in the cytoplasm. All proteins were mainly expressed in PCTECs (Figures 3A,D).

To further validate protein expression levels, WB was performed to analyze three proteins in each group’s kidneys. Given the impact of necrotic/shed epithelial cells, β-actin (for all kidney cells) and AQP1 (for PCTECs) were respectively used as internal references. Results showed no significant upregulation of Wnt7b, β-catenin, and MMP7 with β-actin as reference (Figures 3B,E), but showed their upregulation in AAⅠ-induced mouse kidneys with AQP1 as reference (Figures 3C,F).

### 3.4 Ultrastructural localization of Wnt7b and β-catenin in PCTECs via immunoelectron microscopy

**FIGURE 4:**
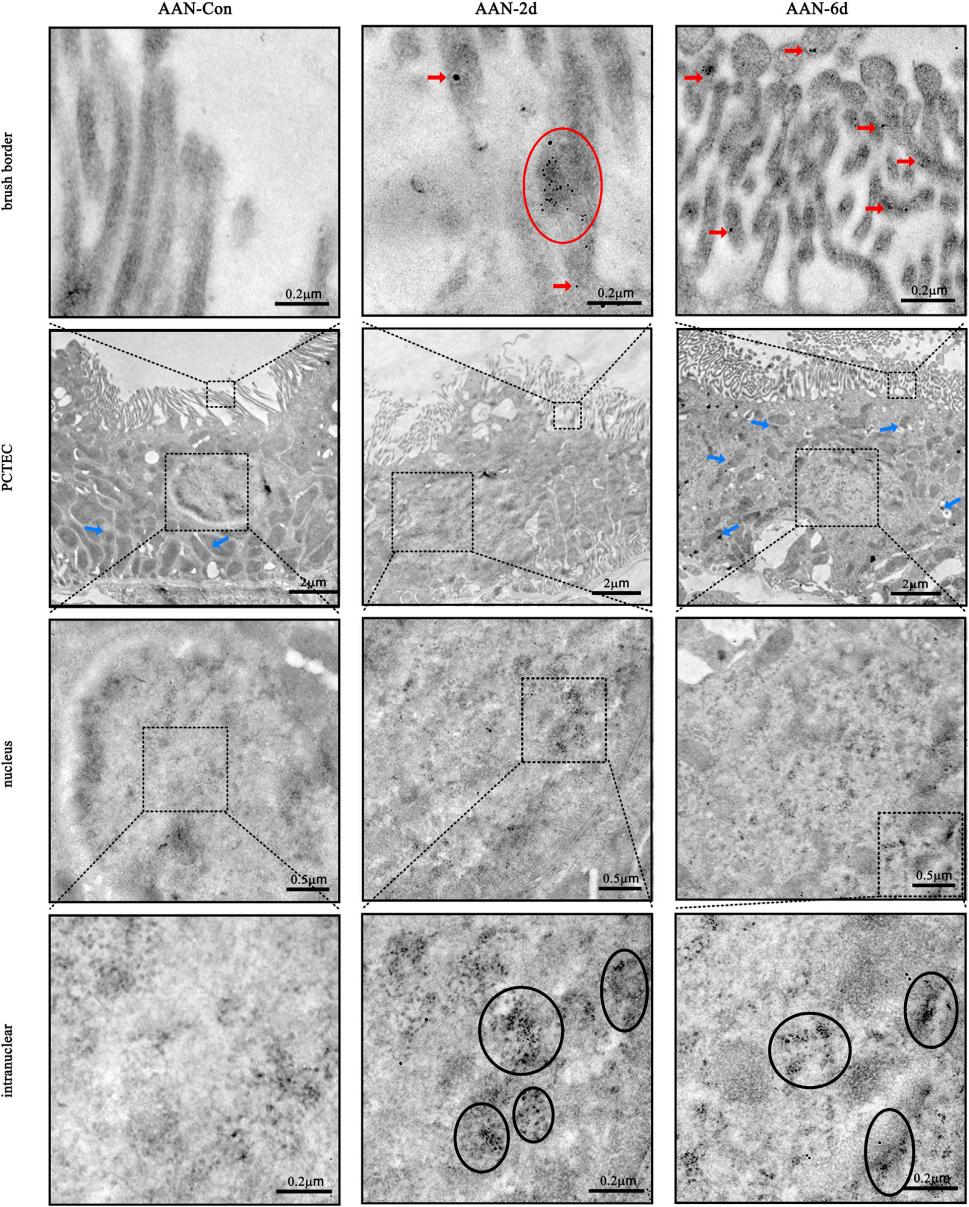
Ultrastructural localization of Wnt7b and β-catenin. Red arrows and circles highlight the presence of Wnt7b (tagged with 12 nm gold nanoparticles). Black circles denote the location of β-catenin (labeled with 4 nm gold nanoparticles). Blue arrows point to mitochondria.

In the control group, the villi of PCTECs were orderly arranged, and the mitochondria presented a normal morphology with distinct cristae. In contrast, in the AAN group, on the 6th day, the number of mitochondria was diminished, their shapes were altered, and structural fractures were observed. In the acute AAN group, Wnt7b was clustered at the boundaries of PCTEC villi. The β-catenin was progressively decreased in the cell cortex but accumulated in the nucleus (Figure 4).

### 3.5 The levels of Wnt7b, β-catenin and MMP7 proteins in HK-2 cells with AAⅠ

**FIGURE 5:**
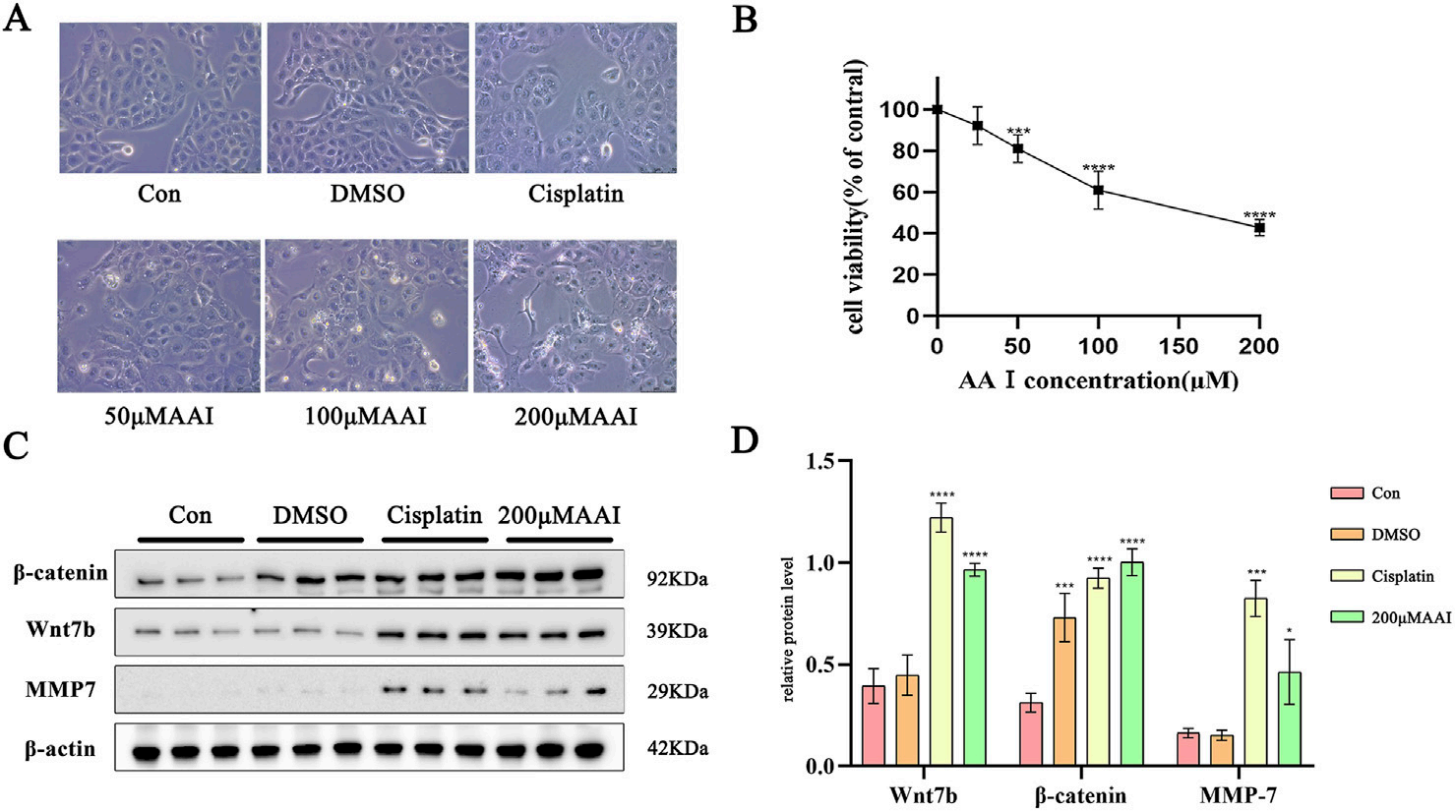
Response of HK-2 cells to AAI exposure. **(A)** Morphological changes of HK-2 cells. **(B)** Cell viability of HK-2 cells. **(C)** Levels of Wnt7b, β-catenin and MMP7 protein. **(D)** Analysis of Wnt7b, β-catenin and MMP7 in HK-2 cells. Data are represented as mean ± SD with at least three independent experiments. *p < 0.05, ***p < 0.005, ****p < 0.001vs. Con.

Upon exposure to AAⅠ, the typical epithelial morphology of HK-2 cells was transformed into a long spindle shape, as depicted in Figure 5A. In contrast to the control group, the cell viability, which had a negative correlation with the concentration of AAⅠ treatment, dropped to 50% when the cells were treated with 200 µM AAⅠ (Figure 5B). WB results indicated that the expressions of Wnt7b, β-catenin, and MMP7 were significantly upregulated (Figures 5C,D).

### 3.6 PCTECs regeneration and expression levels of AQP1, TOP2A, VCAM-1 and α-SMA on chronic AAN

**FIGURE 6:**
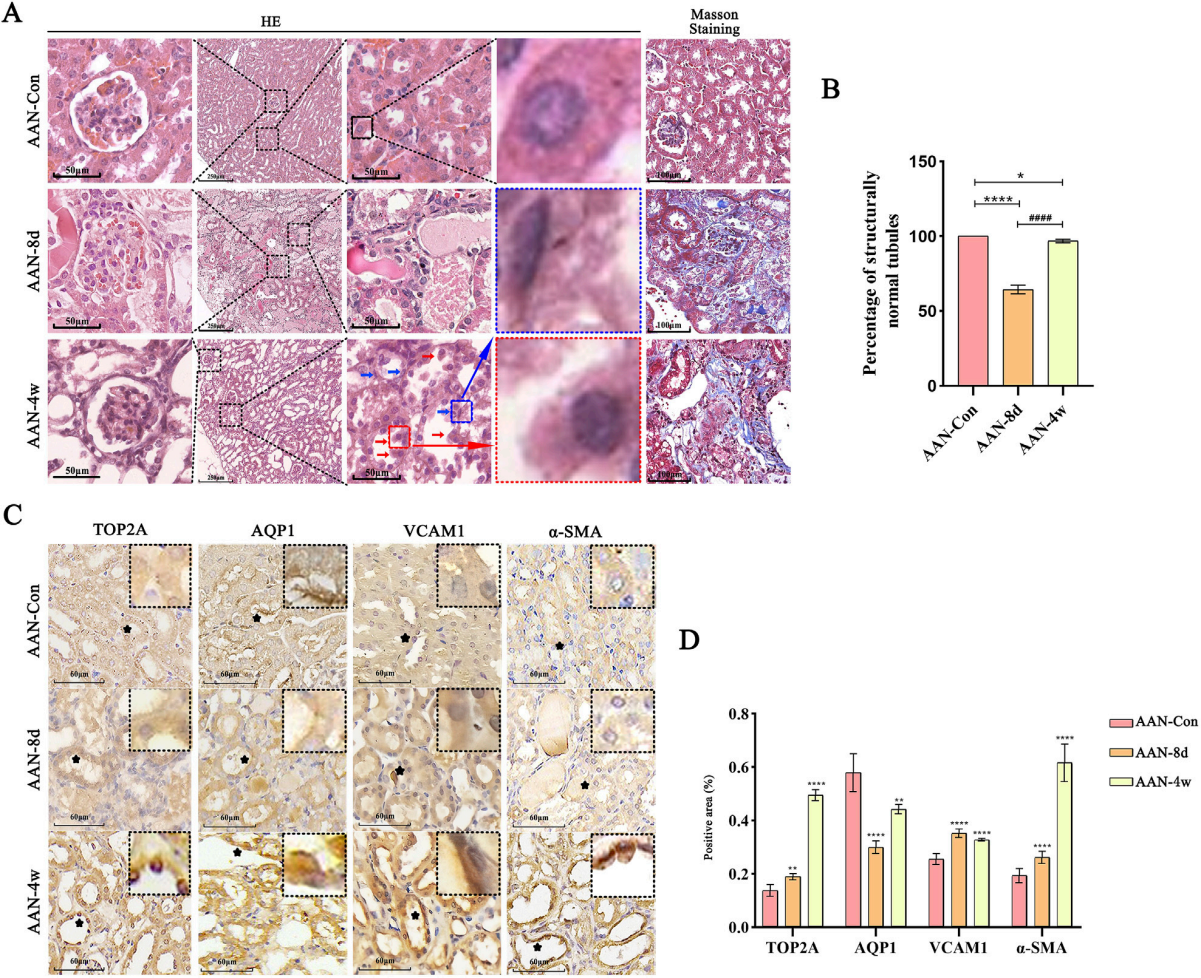
PCTECs regeneration and expression levels of AQP1, TOP2A, VCAM-1 and α-SMA. **(A)** Representative images of HE staining. Blue arrows indicate regenerated flat cells, red arrows indicate regenerated columnar cells. **(B)** Proportion of seemingly normal tubules (sn-PCTs) **(C)** Representative images of AQP1, TOP2A, VCAM-1 and α-SMA assayed by IHC staining. **(D)** OD analysis of proteins in IHC. **p < 0.01, ****p < 0.001 vs. AAN-Con*;* ^*####*^ *p < 0.001* vs. AAN-8d.

By the 8th day after exposure to AAⅠ, the PCTECs underwent necrosis and exfoliated from the basement membrane, and 60% of the tubules were left with only the bare basement membrane, showing an epithelial cell-deficient state (Figures 1C, 6A). However, 4 weeks later, seemingly normal PCTs (sn-PCTs) emerged. Here, the regenerated epithelial cells had altered morphologies: they were newly formed, arranged sawtooth-shaped with enlarged cellular spaces, flat or columnar, and had nuclei that stained deeply. Eventually, PCTs with bare basement membranes disappeared completely from the kidney (Figures 6A,B). After an exposure period of 8 days and 4 weeks, collagen fibers in the kidneys were detected (Figure 6A). IHC analysis revealed that, compared with the control group, TOP2A was highly expressed in the nuclei of PCTECs in mice at 4 weeks. Regarding AQP1, its expression level rebounded on the apical membrane of PCTECs by 4 weeks, but remained relatively low compared to controls. Additionally, the expression levels of VCAM-1 and α-SMA were elevated in mice following 4 weeks of AAⅠ induction (Figures 6C,D).

## 4 Discussion

**FIGURE 7:**
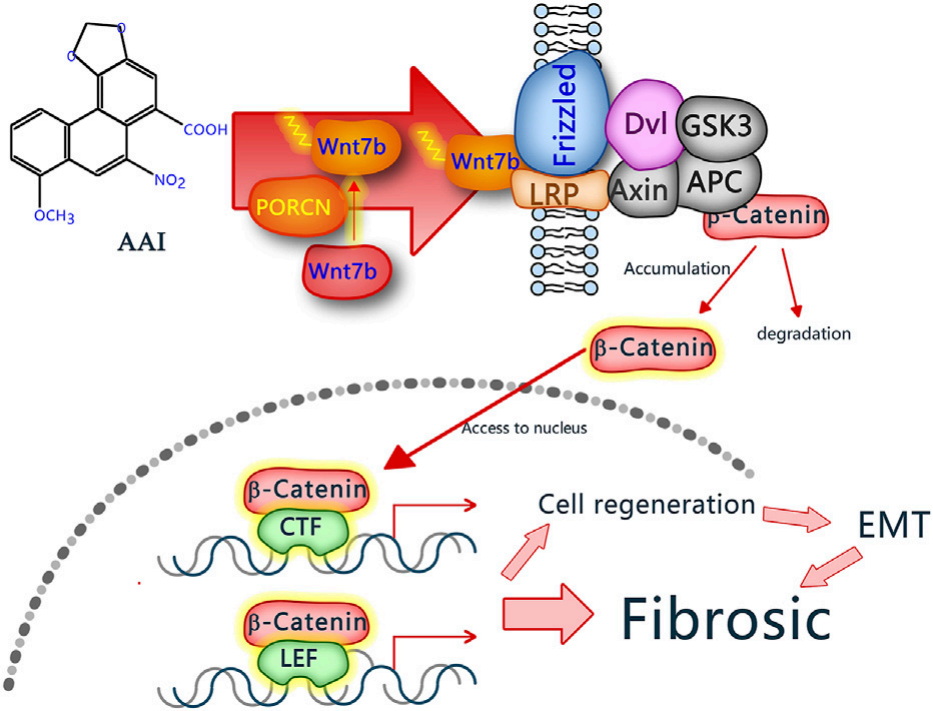
Mechanism of Wnt7b promoting renal fibrosis in AAN. When PCTECs are exposed to AAⅠ, the expression level of the Wnt7b protein rises. Subsequently, it is secreted extracellularly and activated by porcupine O-acyltransferase (PORCN). Once activated, Wnt7b attaches to receptors on the cell membrane, thus triggering the Wnt/β-catenin signaling pathway. As a result, downstream proteins of this pathway are synthesized, ultimately promoting the epithelial-mesenchymal transition of PCTECs, which is associated with renal fibrosis.

It is widely acknowledged across the globe that a large number of cases of chronic kidney diseases with no clear cause are suspected to be related to herbal medicines containing aristolochic acids (Baudoux et al., 2022). The findings of this study demonstrate that Wnt7b activates Wnt/β-catenin pathway, which then guides the injury and repair processes in AAN. The specific mechanism is depicted in Figure 7.

AAI is transported into RTECs via the organic anion transporters OAT1 and OAT3 (Li et al., 2020). Once inside the RTECs, AAI triggers several harmful processes. It generates reactive oxygen species (ROS) (Wang et al., 2022), which can damage cellular components. It also causes mitochondrial dysfunction and DNA damage, and it amplifies inflammatory responses (Ren et al., 2020). Ultimately, these effects lead to the death of RTECs (Zeng et al., 2014). Collectively, these pathological events disrupt the structure of the renal tubules and impair kidney function. Our experimental findings closely match these known mechanisms. Through continuous observation, we saw how renal tubular epithelial cells were progressively damaged. On day 2, we observed cellular swelling. By day 8, epithelial cells began to shedding, particularly in the PCTECs. The expression of KIM-1, a specific biomarker for renal tubular injury (Cuesta et al., 2022), also increases over time. Meanwhile, the fluctuations in BUN and Scr indicate that renal function deteriorates steadily as time goes by Shen et al. (2022). Compared to the extent of damage to PCTECs, only mild edema was noticed in DCT and CT. This is probably because OAT1/3 are predominantly situated within the PCTECs (Motohashi et al., 2013). They play a role in facilitating the transportation of AAⅠ, which subsequently leads to the accumulation of AAⅠ within PCTECs (Xue et al., 2011). Hence, we infer that AAⅠ may mainly trigger renal injury or fibrosis through the initial damage to PCTECs.

We selected both the 4-day and 4-week timepoint for transcriptome sequencing to comprehensively capture Wnt7b-mediated downstream transcriptional alterations, as Wnt pathway activation requires temporal progression following initial Wnt7b upregulation. At the 4-day timepoint, the mRNA expression of Wnt7b showed no significant differences at this early stage, which was consistent with the early adaptive response of the cells in our study. At this time, the cells were still in the adaptation phase and might not have accurately reflected the sustained activation of EMT signaling pathway. This observation was confirmed by our pathological and Wnt protein analyses in the ANN-4d group. We further analyzed our own data on kidney tissues from AAN-4w and compared them with a published study (Chen et al., 2022). We found that the mRNA levels of several proteins related to the Wnt/β-catenin signaling pathway, such as Snail2, Axin2, MMP7, Zeb1, and Hgf, were significantly higher. During chronic kidney scarring, regenerated cells may help repair tissues by rebuilding damaged basement membranes. They might also contribute to collagen buildup and fibrotic remodeling. In our subsequent experiments, we exposed PCTECs to AAI injury and observed a significant increase in the expression of Wnt7b, β-catenin, and MMP7. The same increase was also seen in HK-2 cells. Previous research has shown that Wnt ligands are activated when PORCN in mitochondria attaches palmitoyl-CoA to the serine residues of Wnt proteins (Liu et al., 2022). Once activated, these Wnt ligands are transported extracellularly and bind to membrane receptors (here strongly confirmed to accumulate in the villi of PCTECs via IHC and Immunoelectron assay), thereby triggering the Wnt/β-catenin signaling pathway (Rim et al., 2022). This activation further promotes the expression of β-catenin and MMP7, a downstream protein of the Wnt/β-catenin signaling pathway (Tan et al., 2019). The accumulation of β-catenin in the cytoplasm followed by its translocation to the nucleus is the pivotal steps in activating the Wnt/β-catenin signaling pathway (Liu et al., 2022). Further immunoelectron microscopy observations demonstrated that Wnt7b accumulated on the microvilli of PCTECs, while β-catenin translocated to the nucleus. When β-catenin moves into the nucleus, it binds to TCF/LEF transcription factors. This action turns on genes related to the epithelial-mesenchymal transition, such as Snail, Twist, and MMPs. Together, these molecular events push the EMT process forward in renal tubular epithelial cells (Tan et al., 2014). Meanwhile, it is noteworthy that, after AAⅠ induction, the mitochondrial morphology underwent significant changes, and aggregates of β-catenin were observed at the mitochondrial membrane fission sites, which may be associated with the necrosis of PCTECs. These findings strongly suggest that AAⅠ might be involved in the damage and repair processes of PCTECs in acute AAN, potentially through upregulating Wnt7b expression and activating the Wnt signaling pathway.

To further explore the regenerative and reparative capabilities of PCTECs in relation to the renal tubules’ naked basement membrane after the activation of the Wnt/β-catenin signaling pathway, we conducted an investigation into the abundance, morphology, and function of PCTECs in the kidneys of mice exposed to AAⅠ for 4 weeks. Our findings revealed that a large number of morphologically abnormal regenerative cells emerged on the naked basement membrane of PCT. These cells exhibited high expression levels of TOP2A (Liu et al., 2024), a protein closely associated with cell proliferation. These regenerative cells played a role in the repair of damaged renal tubules, giving rise to the appearance of seemingly normal PCTs (sn-PCT). However, the morphology of these epithelial cells of these sn-PCT deviated from that of normal PCT, and they were irregularly arranged. Moreover, they expressed the AQP1 protein (Su et al., 2020), epithelial marker protein, leading to a rebound in its expression level, though this level remained relatively low compared to that in normal PCTECs. Furthermore, the mesenchymal marker proteins VCAM-1 (Zhang et al., 2020) and α-SMA (Piera-Velazquez et al., 2011) were highly expressed, indicating that these cells possessed characteristics of mesenchymal cells. When the Wnt signaling pathway triggers the epithelial-mesenchymal transition, the cells that change (transdifferentiate) can turn into myofibroblasts on their own. These cells also release signaling molecules like connective tissue growth factor (CTGF), platelet-derived growth factor (PDGF), and transforming growth factor-β1 (TGF-β1). These molecules act on nearby fibroblasts, making them produce too much collagen fiber. This speeds up the process of kidney scarring (Sun et al., 2016). Importantly, more and more evidence shows that cells from EMT, along with activated fibroblasts and myofibroblasts, are the main drivers of kidney tissue scarring (Meran and Steadman, 2011). In our experiment, we also noticed a positive correlation between the abundance of tubular EMT cells and the degree of renal interstitial fibrosis in the kidneys of mice treated with AAⅠ for 4 weeks. We hypothesize that while Wnt7b promotes the regeneration of PCTECs, these regenerative cells display dual epithelial and mesenchymal characteristics that are related to renal fibrosis in chronic AAN. Additionally, in the kidneys of mice after 4 weeks, the epithelial cells of sn-PCT did not continue to undergo necrosis or shedding. The primary reason for this might be the partial reduction in the expression of some OAT proteins, which followed a similar trend to that of AQP1. This further attests to the mesenchymal-like characteristics of the regenerative epithelial cells.

Our findings suggest that targeting the Wnt/β-catenin signaling pathway may offer therapeutic potential for AAN. For instance, the drug LGK974 has been shown to effectively inhibit the secretion of Wnt7b protein (Liu et al., 2022). However, our current experimental approach has limitations, including the drug’s lack of specificity as LGK974 affects multiple Wnt proteins. To address this issue, we propose using conditional Wnt7b knockout mice to systematically evaluate kidney injury progression following AAI exposure. This model will clarify whether injury progression is altered in the absence of Wnt7b and whether this pathway exerts distinct effects at different injury stages.

In conclusion, AAⅠ is highly nephrotoxic. It predominantly impairs PCTECs, triggering the activation of the Wnt7b/β-catenin pathway. This, in turn, facilitates the regeneration of PCTECs and the epithelial-mesenchymal transition process, which is closely associated with renal fibrosis. We are convinced that the activation of the Wnt7b/β-catenin signaling pathway constitutes the central pivot in the development of renal fibrosis during AAN.

## Data Availability

The original contributions presented in the study are publicly available. This data can be found here: https://doi.org/10.6084/m9.figshare.28870688.

## Glossary

AAN: Aristolochic Acid Nephropathy
AA I: Aristolochic Acid I
HK-2: Human Renal Tubular Epithelial Cells
PCT: Proximal Convoluted Tubule
DCT: Distal Convoluted Tubule
CT: Collecting Tubule
PT: Proximal Tubule
PCTECs: Proximal Convoluted Tubule Epithelial Cells
Sn-PCTs: Seemingly Normal Pcts
EMT: Epithelial Mesenchymal Transition
Scr: Serum Creatinine
BUN: Blood Urea Nitrogen
KIM-1: Kidney Injury Molecule-1
RTECs: Renal Tubular Epithelial Cells
TOP2A: Topoisomerase Ⅱα
AQP1: Aquaporin 1
VCAM-1: Vascular Cell Adhesion Molecule-1
α-SMA: α-Smooth Muscle Actin
OAT: Organic Anion Transporter Protein
CCK-8: Cell Counting Kit-8
CMC-Na: Carboxymethylcellulose Sodium
IHC: Immunohistochemistry
FBS: Fetal Bovine Serum
MMP7: Matrix Metalloproteinase 7
DMEM: Dulbecco’s Modified Eagle Medium
DMSO: Dissolved In Dimethyl Sulfoxide
ROS: Reactive Oxygen Species
PORCN: Porcupine O-Acyltransferase
MMPs: Matrix Metalloproteinases
CTGF: Connective Tissue Growth Factor
PDGF: Platelet-Derived Growth Factor
