# Supplementary figures and images for: Renal tubular epithelial cell related partial epithelial-mesenchymal transition in AAⅠ induced renal fibrosis via Wnt7b/β-catenin signaling (part 2 of 2)
Source: Front Pharmacol. 2025 May 13;16:1571960. doi: 10.3389/fphar.2025.1571960 (PMC12106489; doi:10.3389/fphar.2025.1571960)

references:  $\beta$ -actin

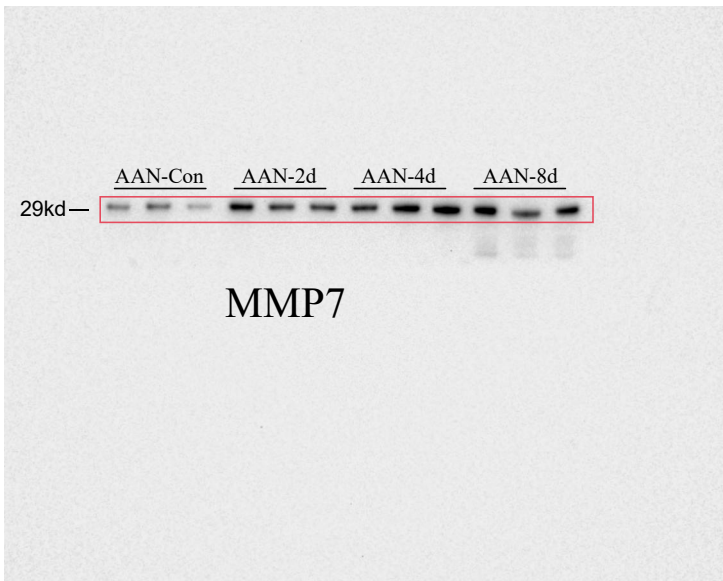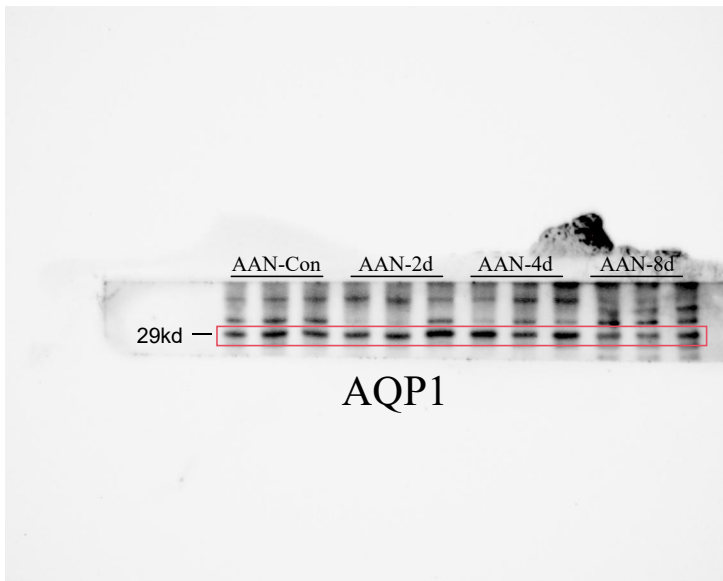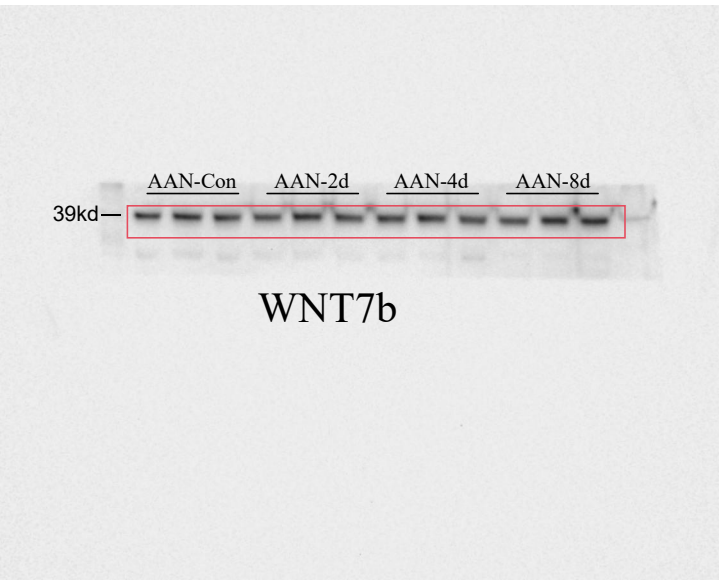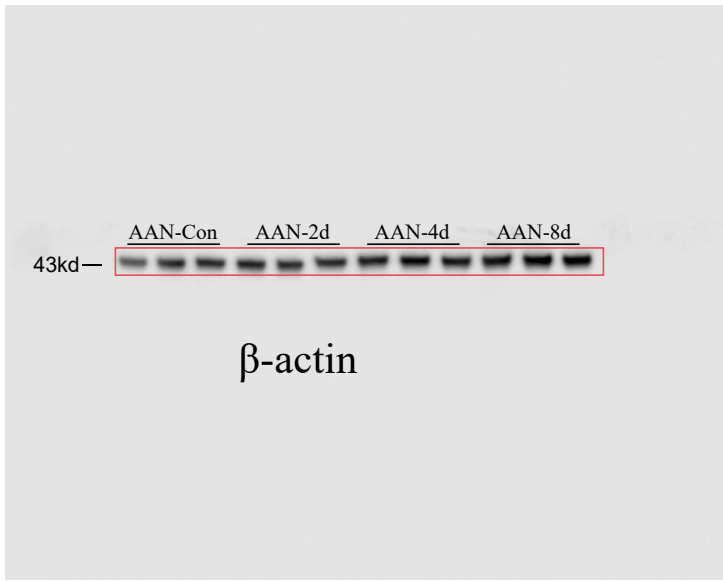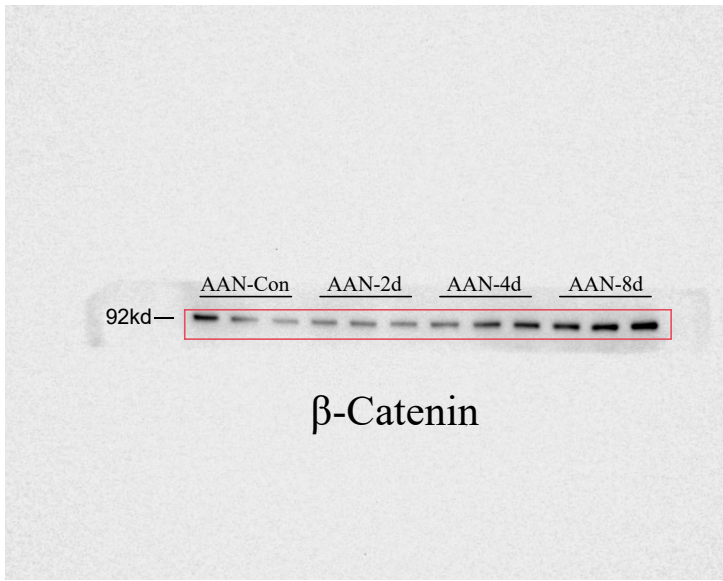

references: AQP1

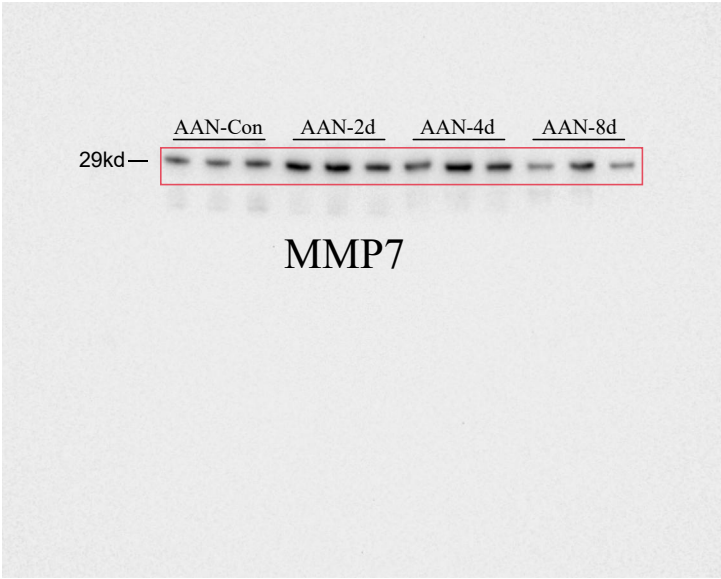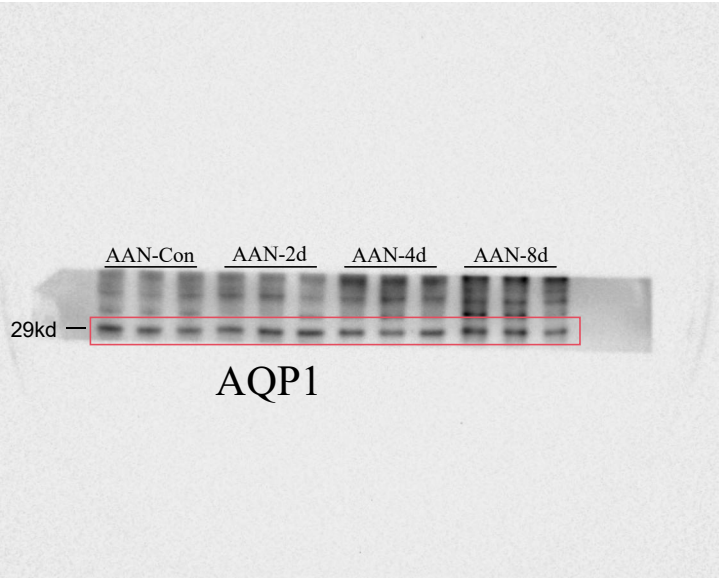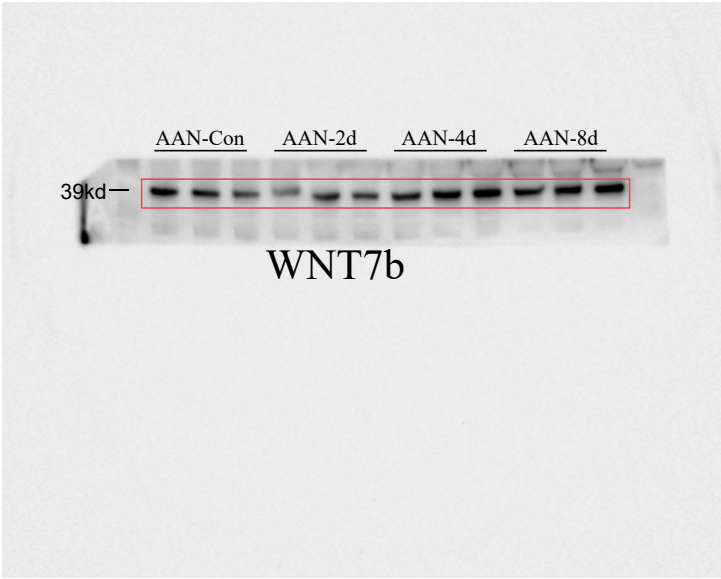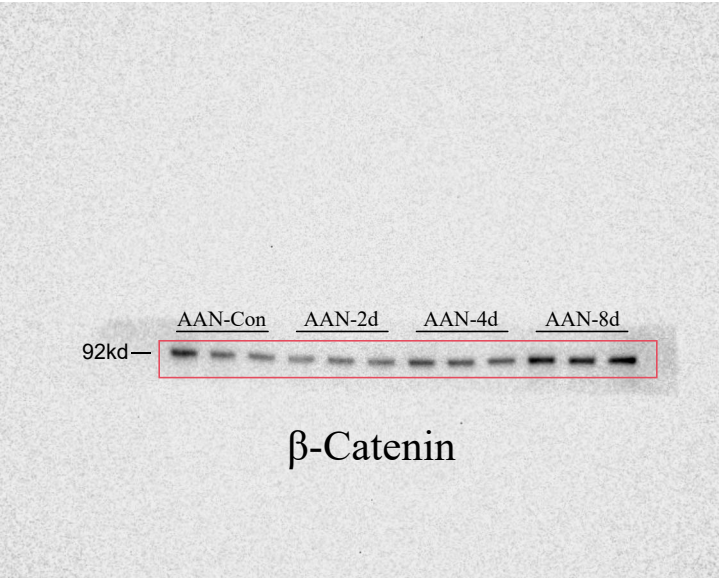

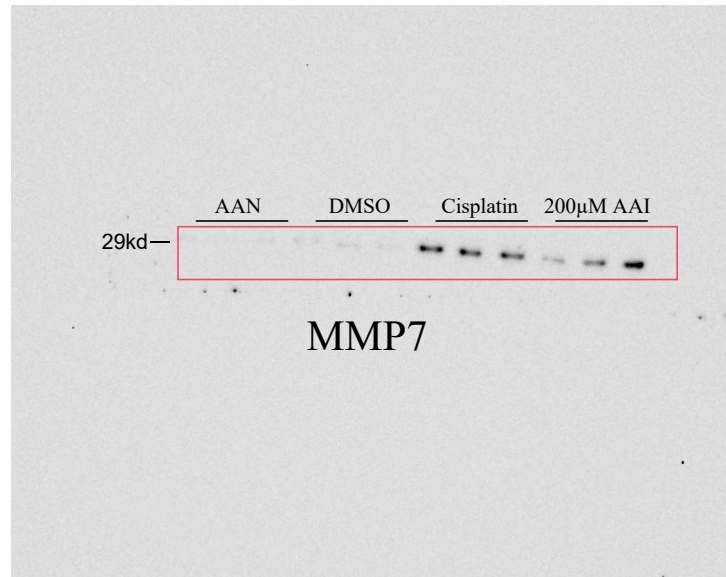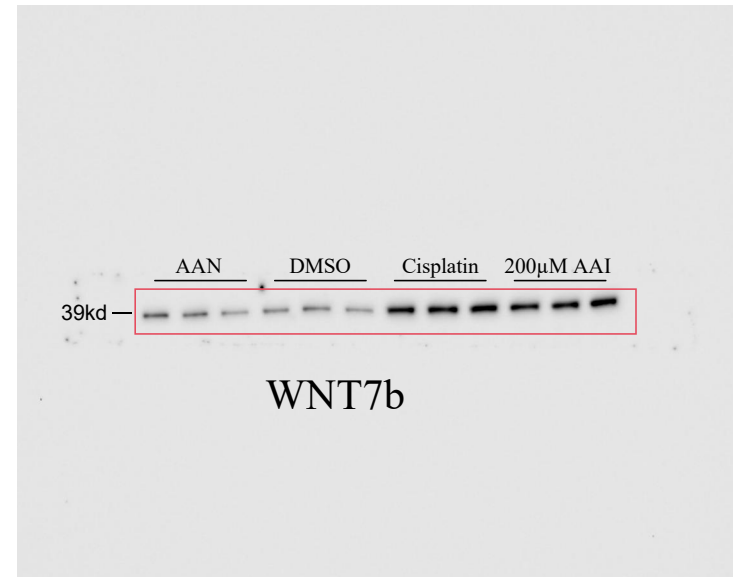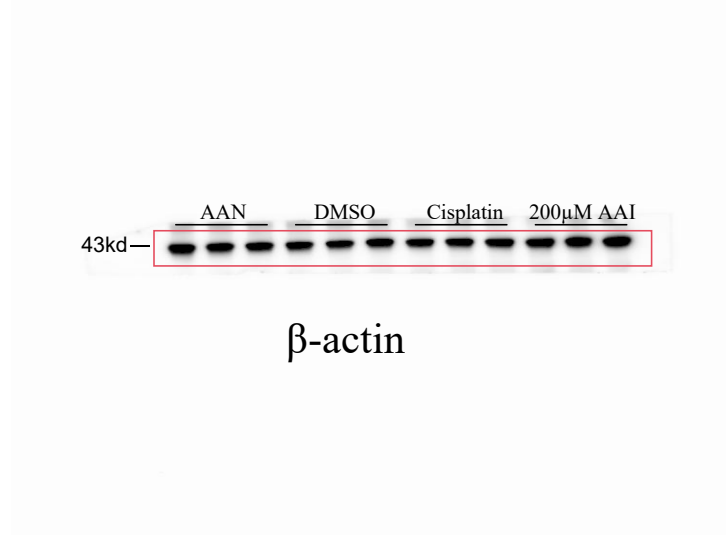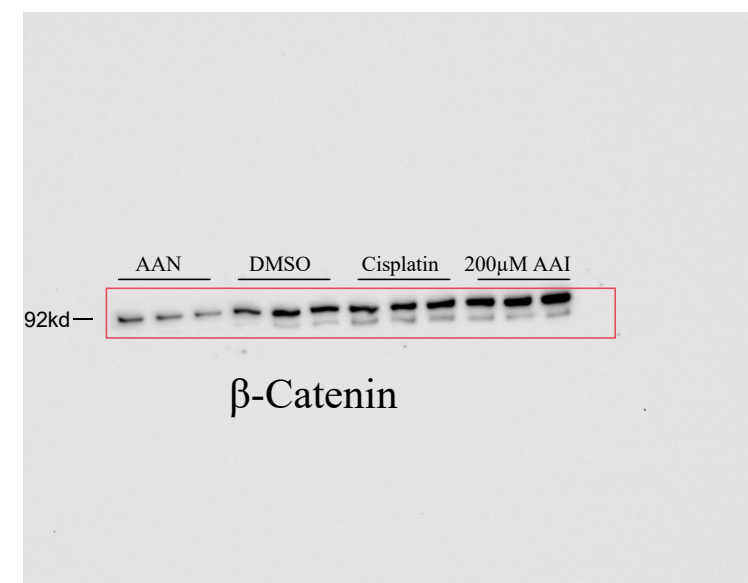

Supplement: Supplementary file 1 [file DataSheet1.zip › Original materials/WB.pdf]
