# Supplementary figures and images for: Renal tubular epithelial cell related partial epithelial-mesenchymal transition in AAⅠ induced renal fibrosis via Wnt7b/β-catenin signaling (part 1 of 2)
Source: Front Pharmacol. 2025 May 13;16:1571960. doi: 10.3389/fphar.2025.1571960 (PMC12106489; doi:10.3389/fphar.2025.1571960)

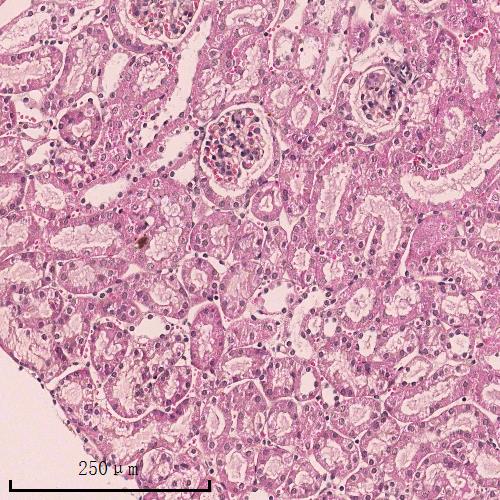

Supplement: Supplementary file 1 [file DataSheet1.zip › Original materials/Microscopic image/Fig 1/HE/AAN-2d 1.jpg]

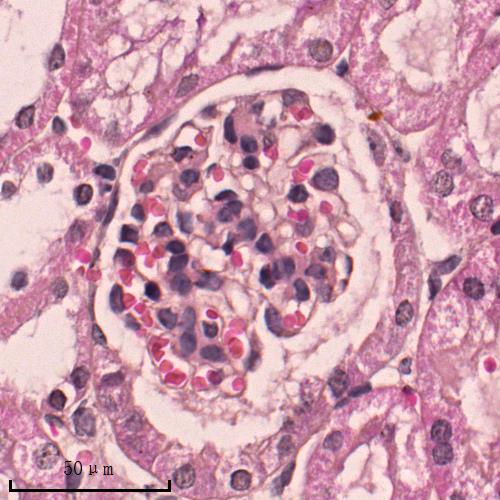

Supplement: Supplementary file 1 [file DataSheet1.zip › Original materials/Microscopic image/Fig 1/HE/AAN-2d 2.jpg]

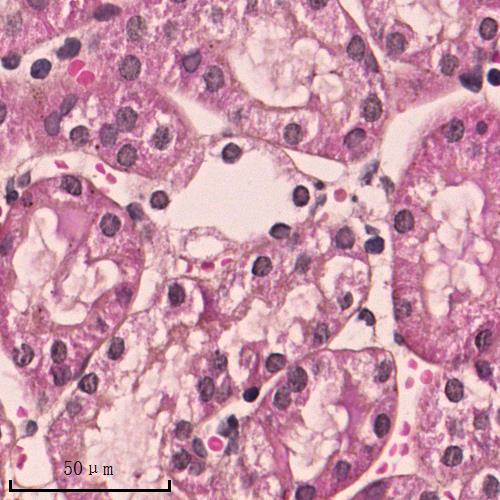

Supplement: Supplementary file 1 [file DataSheet1.zip › Original materials/Microscopic image/Fig 1/HE/AAN-2d 3.jpg]

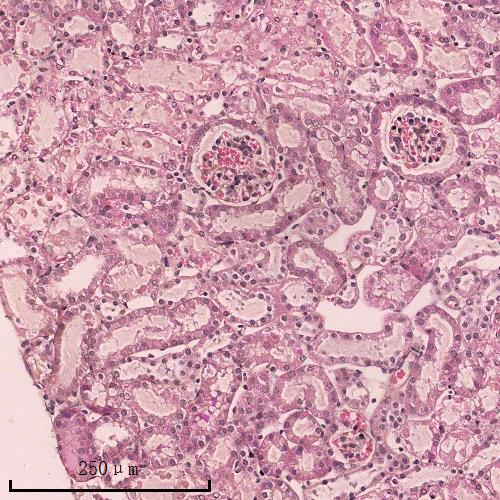

Supplement: Supplementary file 1 [file DataSheet1.zip › Original materials/Microscopic image/Fig 1/HE/AAN-4d 1.jpg]

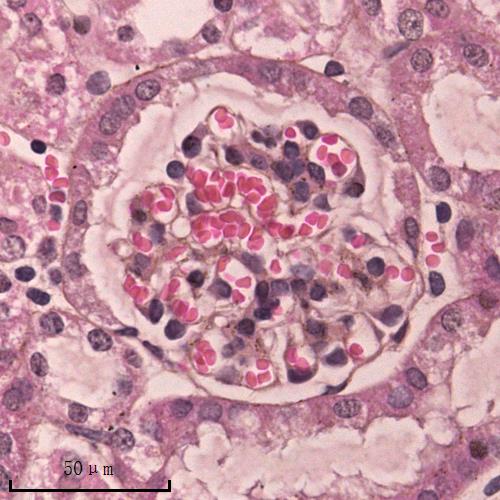

Supplement: Supplementary file 1 [file DataSheet1.zip › Original materials/Microscopic image/Fig 1/HE/AAN-4d 2.jpg]

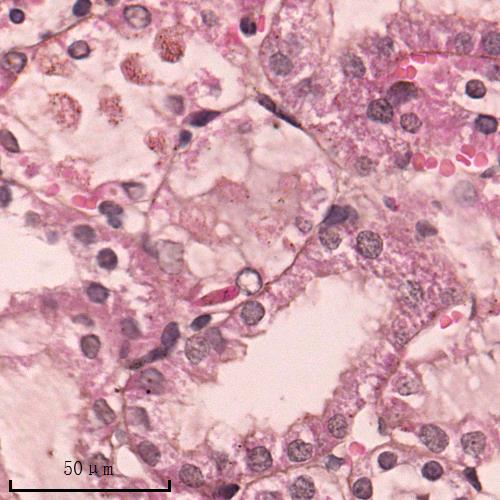

Supplement: Supplementary file 1 [file DataSheet1.zip › Original materials/Microscopic image/Fig 1/HE/AAN-4d 3.jpg]

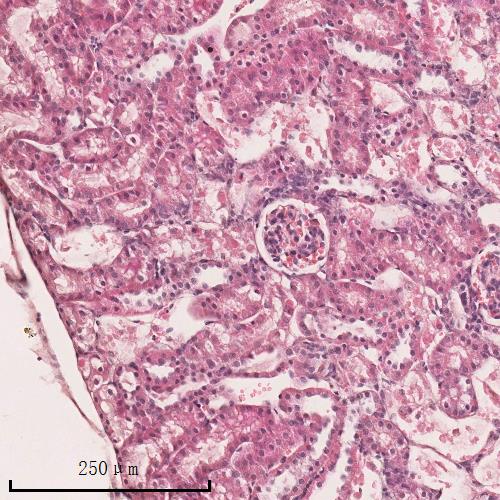

Supplement: Supplementary file 1 [file DataSheet1.zip › Original materials/Microscopic image/Fig 1/HE/AAN-6d 1.jpg]

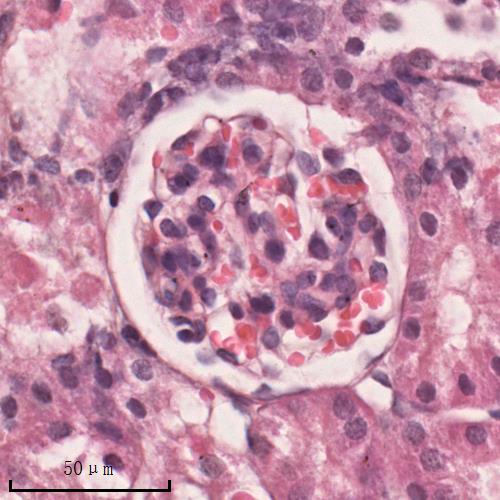

Supplement: Supplementary file 1 [file DataSheet1.zip › Original materials/Microscopic image/Fig 1/HE/AAN-6d 2.jpg]

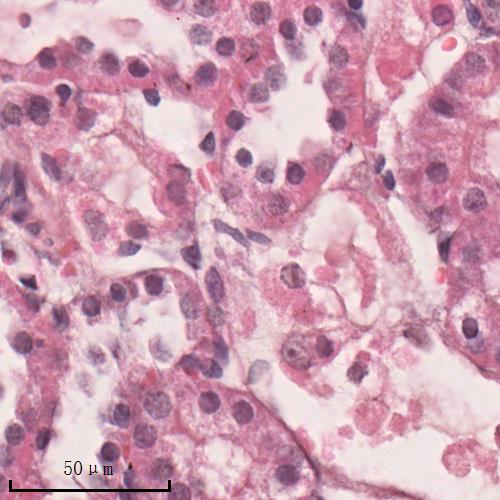

Supplement: Supplementary file 1 [file DataSheet1.zip › Original materials/Microscopic image/Fig 1/HE/AAN-6d 3.jpg]

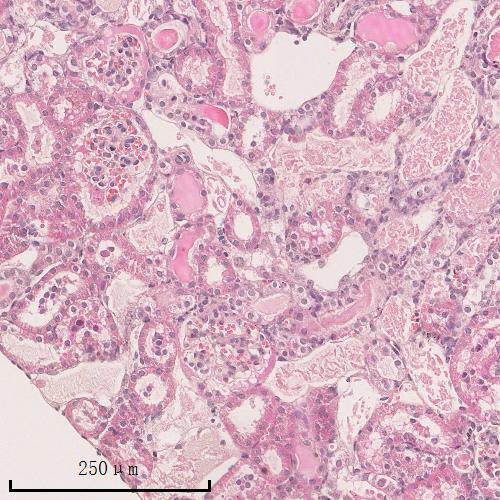

Supplement: Supplementary file 1 [file DataSheet1.zip › Original materials/Microscopic image/Fig 1/HE/AAN-8d 1.jpg]

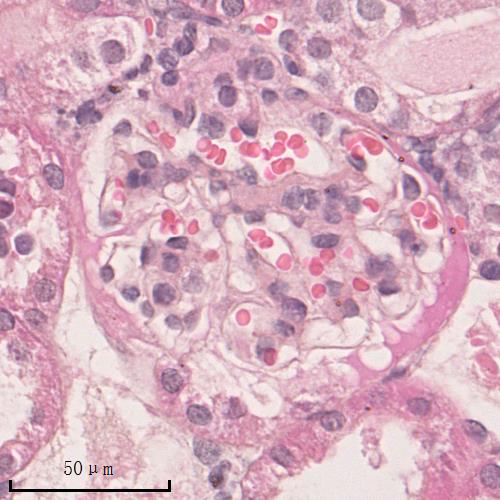

Supplement: Supplementary file 1 [file DataSheet1.zip › Original materials/Microscopic image/Fig 1/HE/AAN-8d 2.jpg]

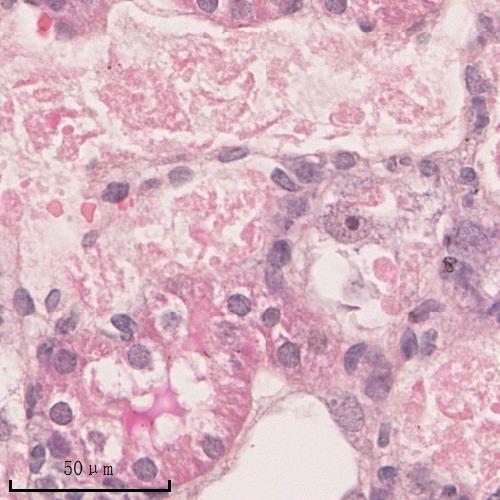

Supplement: Supplementary file 1 [file DataSheet1.zip › Original materials/Microscopic image/Fig 1/HE/AAN-8d 3.jpg]

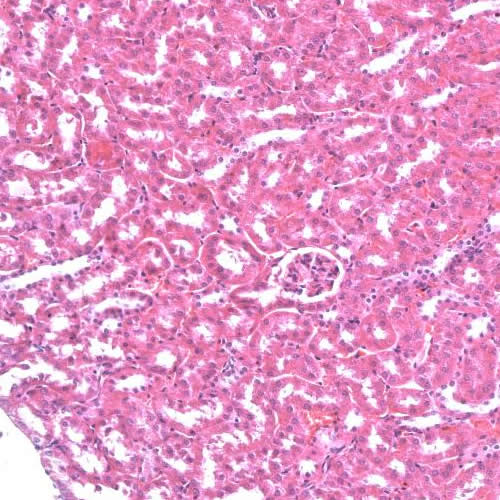

Supplement: Supplementary file 1 [file DataSheet1.zip › Original materials/Microscopic image/Fig 1/HE/AAN-Con 1.jpg]

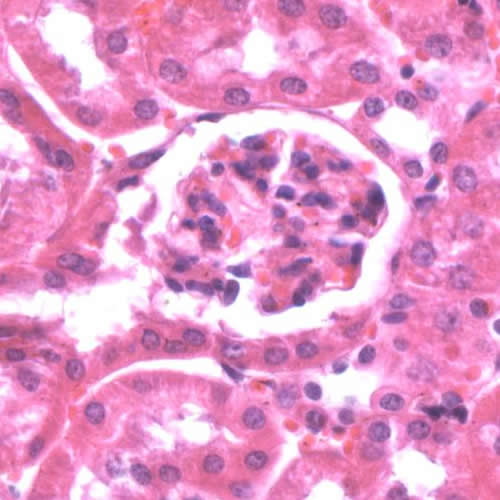

Supplement: Supplementary file 1 [file DataSheet1.zip › Original materials/Microscopic image/Fig 1/HE/AAN-Con 2.jpg]

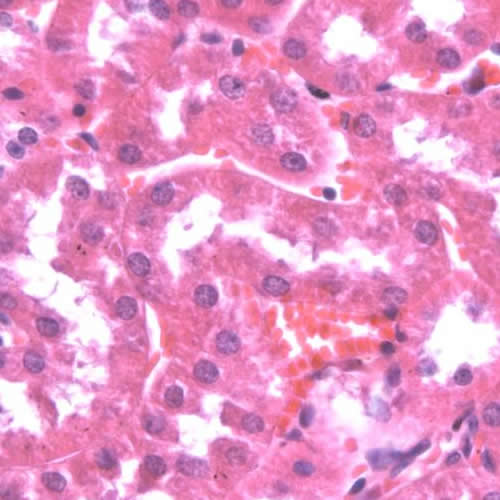

Supplement: Supplementary file 1 [file DataSheet1.zip › Original materials/Microscopic image/Fig 1/HE/AAN-Con 3.jpg]

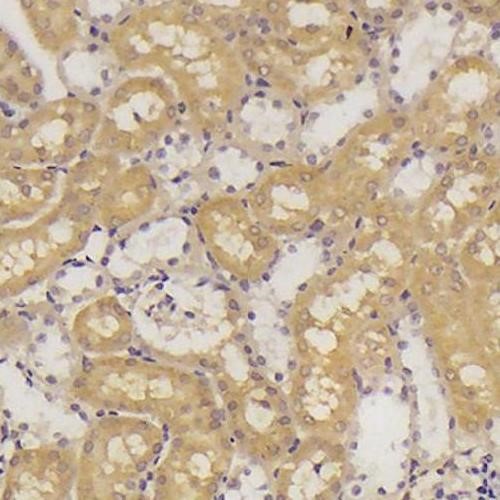

Supplement: Supplementary file 1 [file DataSheet1.zip › Original materials/Microscopic image/Fig 1/KIM-1/AAN-2d.jpg]

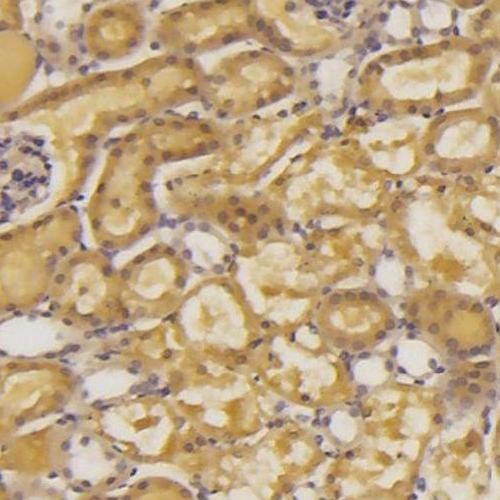

Supplement: Supplementary file 1 [file DataSheet1.zip › Original materials/Microscopic image/Fig 1/KIM-1/AAN-4d.jpg]

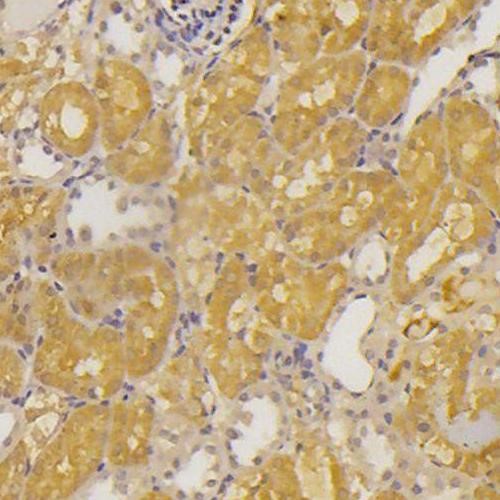

Supplement: Supplementary file 1 [file DataSheet1.zip › Original materials/Microscopic image/Fig 1/KIM-1/AAN-6d.jpg]

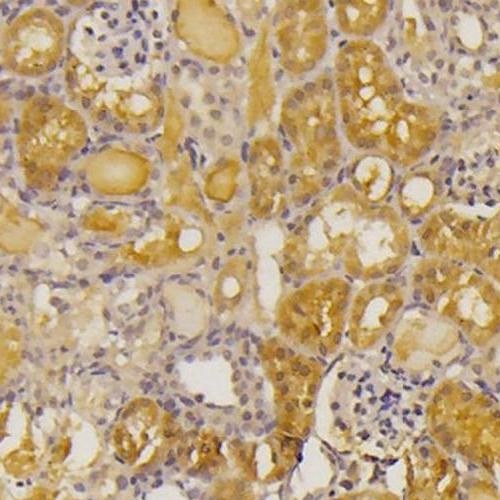

Supplement: Supplementary file 1 [file DataSheet1.zip › Original materials/Microscopic image/Fig 1/KIM-1/AAN-8d.jpg]

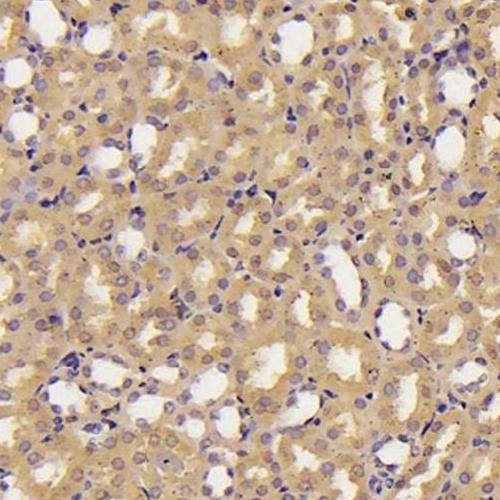

Supplement: Supplementary file 1 [file DataSheet1.zip › Original materials/Microscopic image/Fig 1/KIM-1/AAN-Con.jpg]

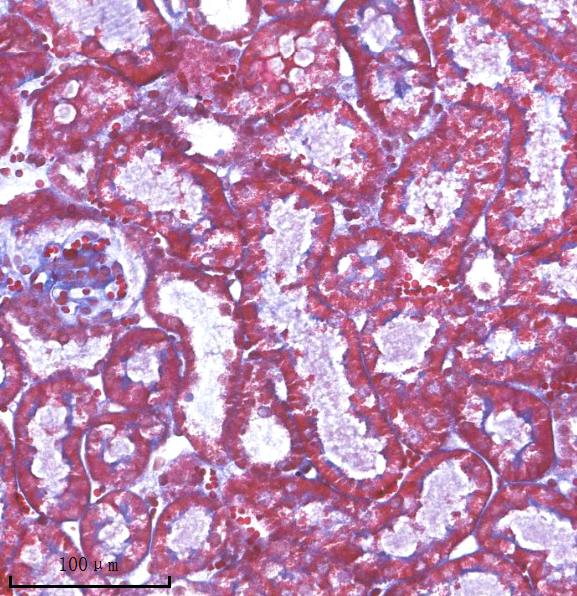

Supplement: Supplementary file 1 [file DataSheet1.zip › Original materials/Microscopic image/Fig 1/Masson/AAN-2d.jpg]

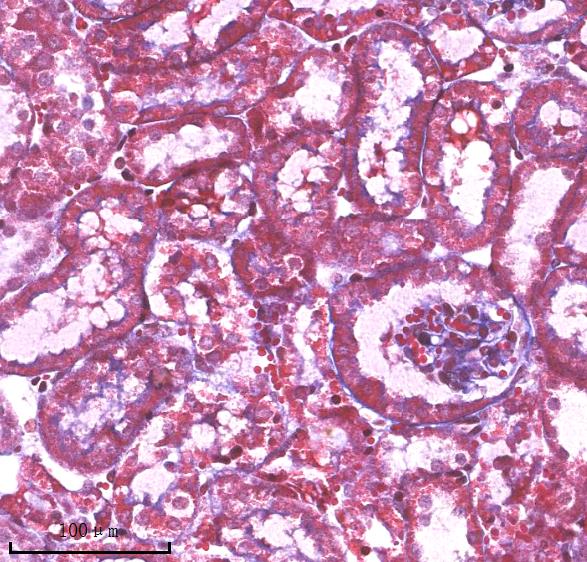

Supplement: Supplementary file 1 [file DataSheet1.zip › Original materials/Microscopic image/Fig 1/Masson/AAN-4d.jpg]

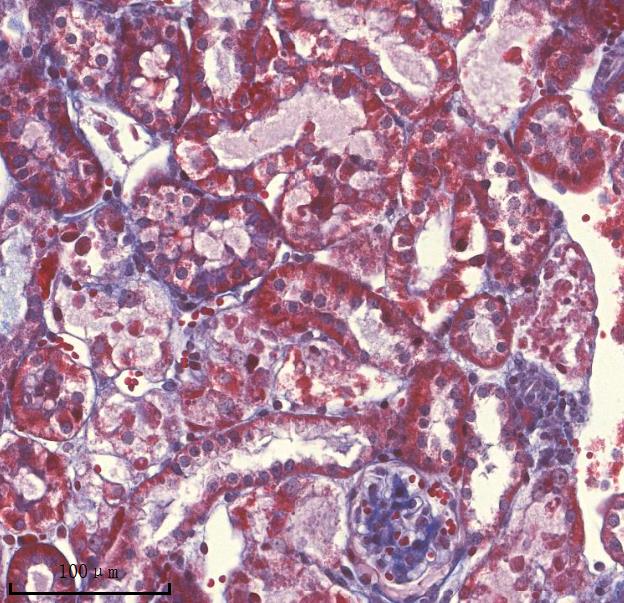

Supplement: Supplementary file 1 [file DataSheet1.zip › Original materials/Microscopic image/Fig 1/Masson/AAN-6d.jpg]

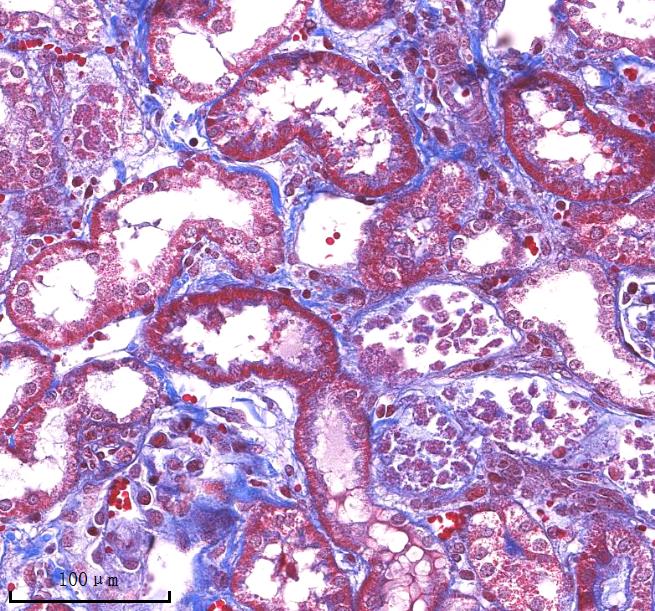

Supplement: Supplementary file 1 [file DataSheet1.zip › Original materials/Microscopic image/Fig 1/Masson/AAN-8d.jpg]

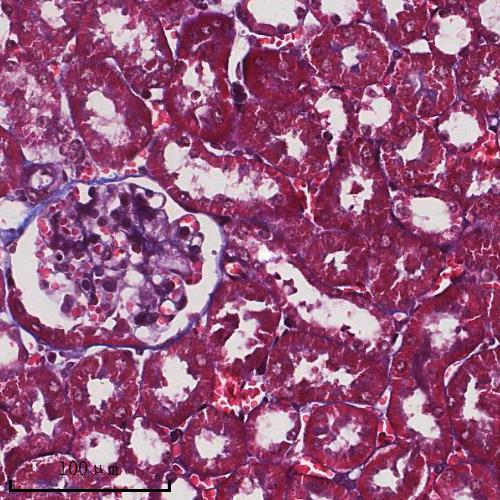

Supplement: Supplementary file 1 [file DataSheet1.zip › Original materials/Microscopic image/Fig 1/Masson/AAN-Con.jpg]

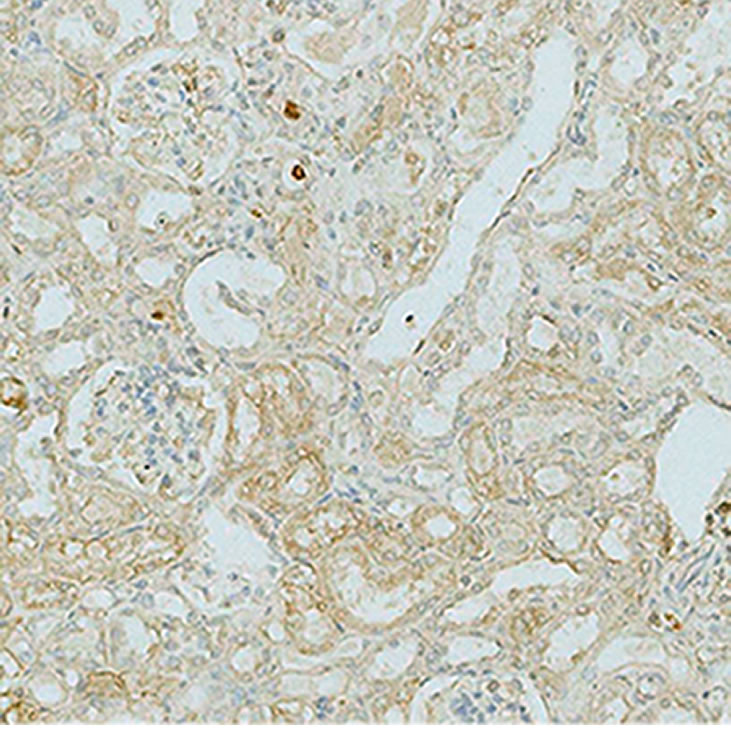

Supplement: Supplementary file 1 [file DataSheet1.zip › Original materials/Microscopic image/Fig 3/MMP7/AAN-2d 1.jpg]

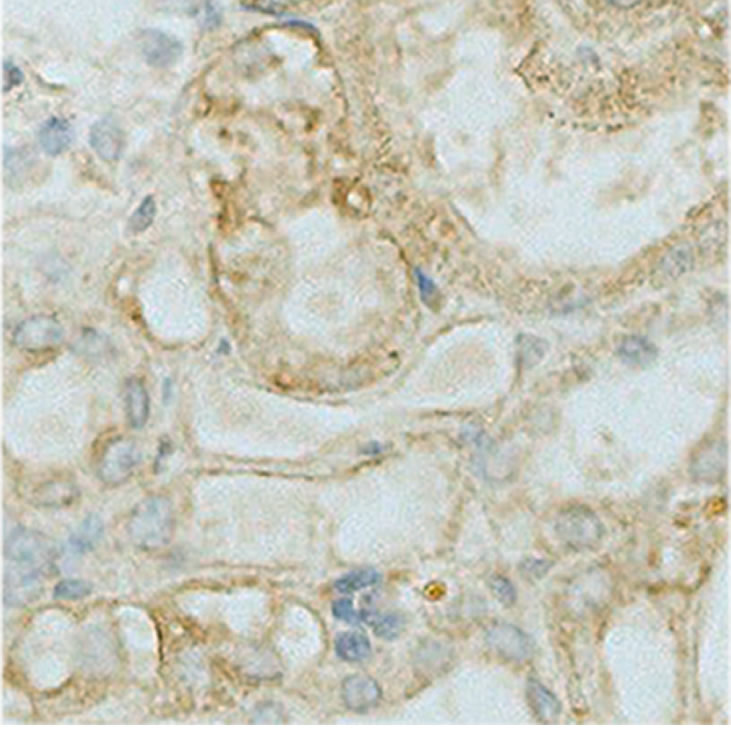

Supplement: Supplementary file 1 [file DataSheet1.zip › Original materials/Microscopic image/Fig 3/MMP7/AAN-2d 2.jpg]

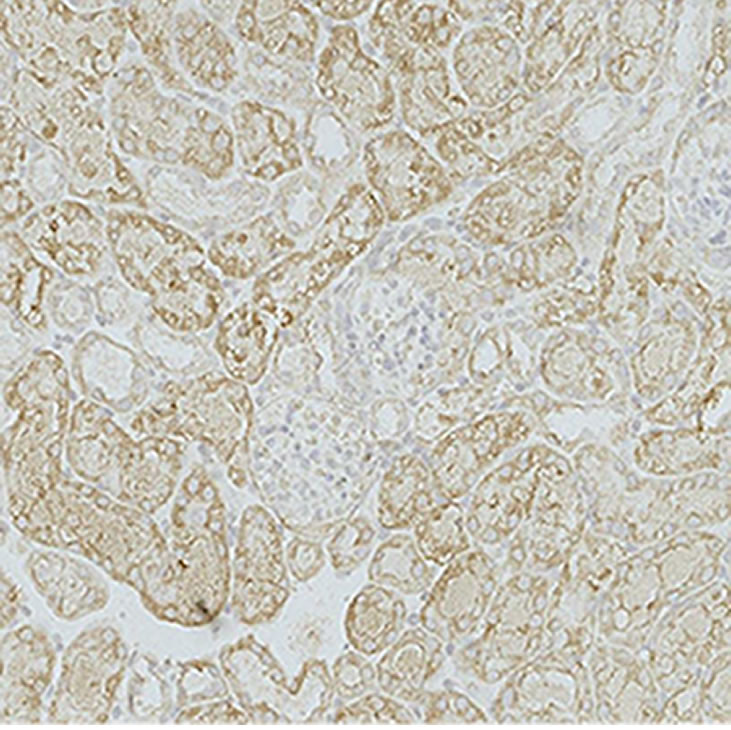

Supplement: Supplementary file 1 [file DataSheet1.zip › Original materials/Microscopic image/Fig 3/MMP7/AAN-4d 1.jpg]

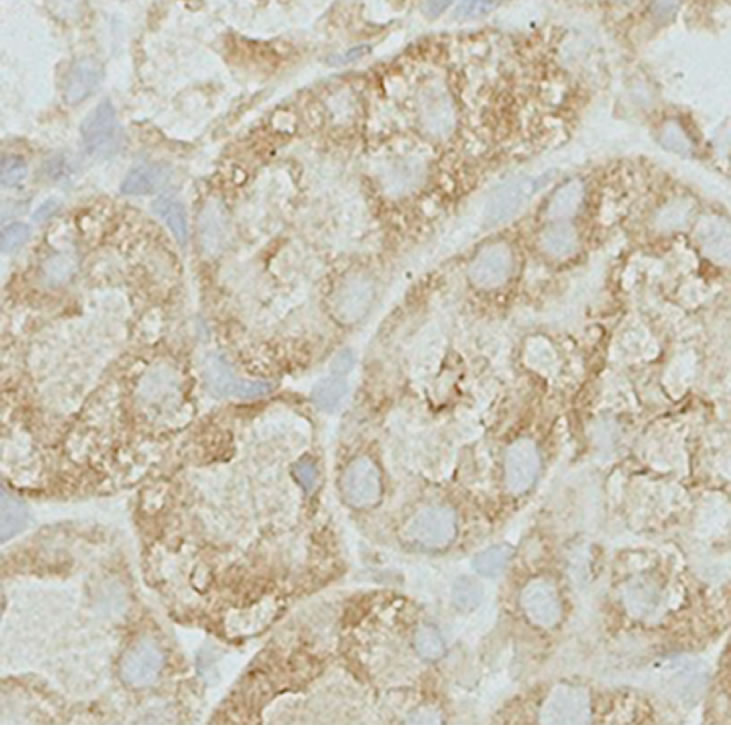

Supplement: Supplementary file 1 [file DataSheet1.zip › Original materials/Microscopic image/Fig 3/MMP7/AAN-4d 2.jpg]

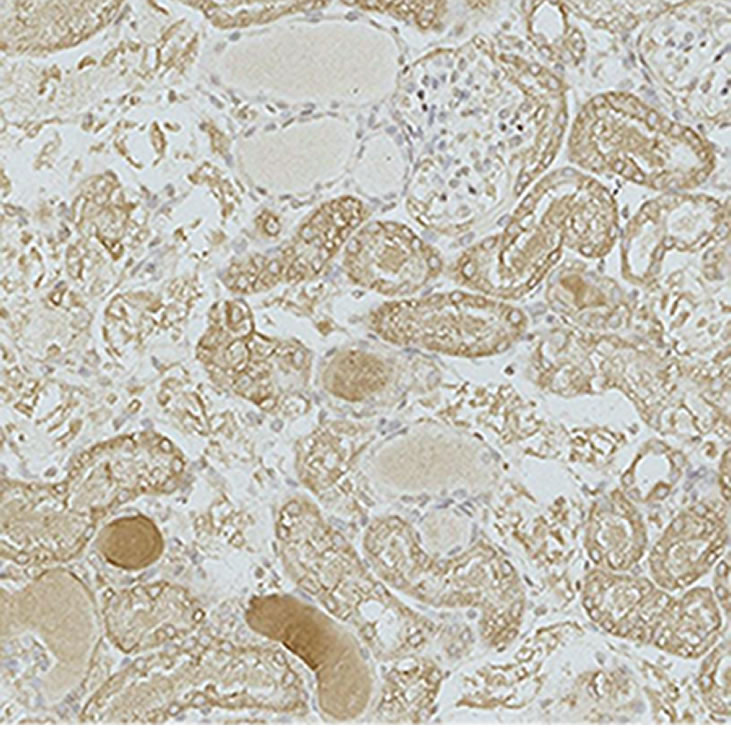

Supplement: Supplementary file 1 [file DataSheet1.zip › Original materials/Microscopic image/Fig 3/MMP7/AAN-6d 1.jpg]

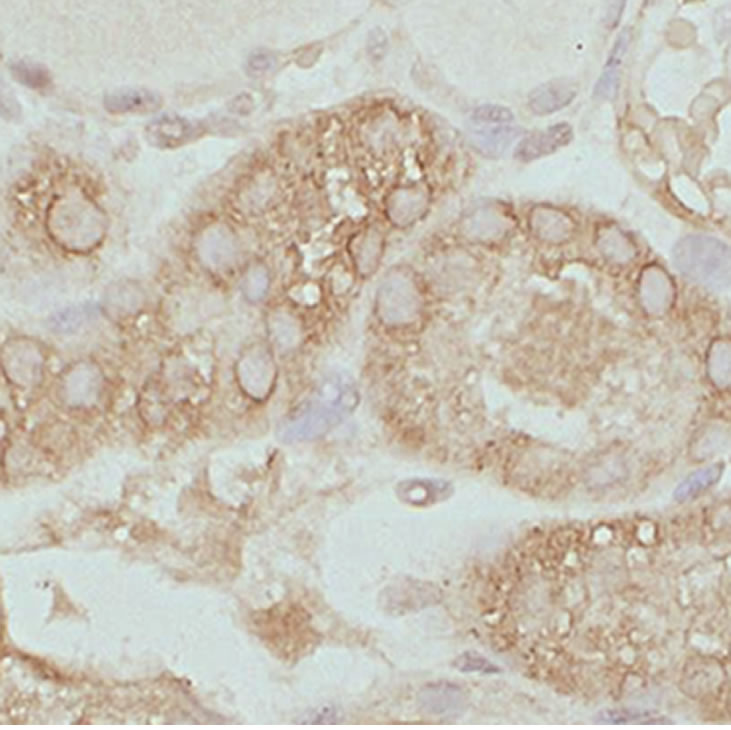

Supplement: Supplementary file 1 [file DataSheet1.zip › Original materials/Microscopic image/Fig 3/MMP7/AAN-6d 2.jpg]

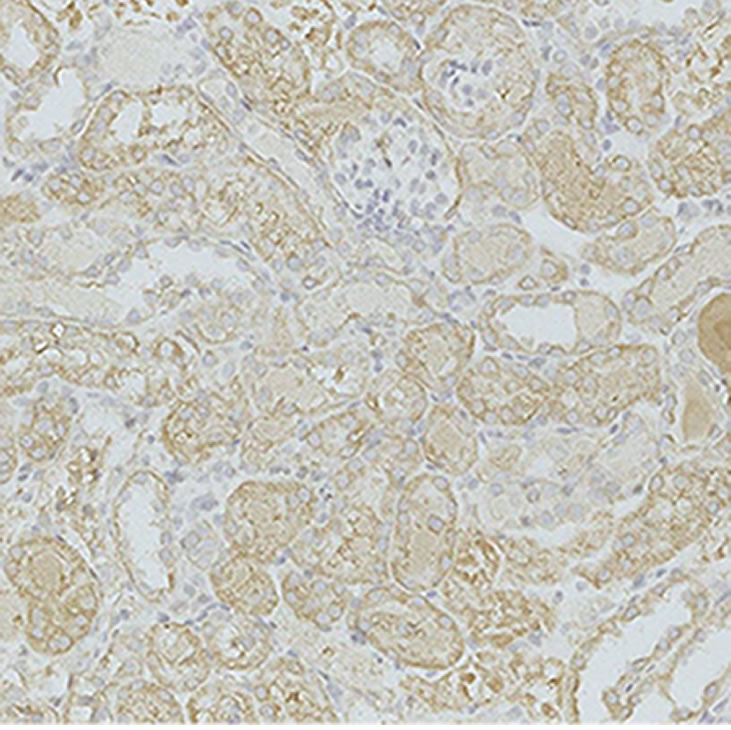

Supplement: Supplementary file 1 [file DataSheet1.zip › Original materials/Microscopic image/Fig 3/MMP7/AAN-8d 1.jpg]

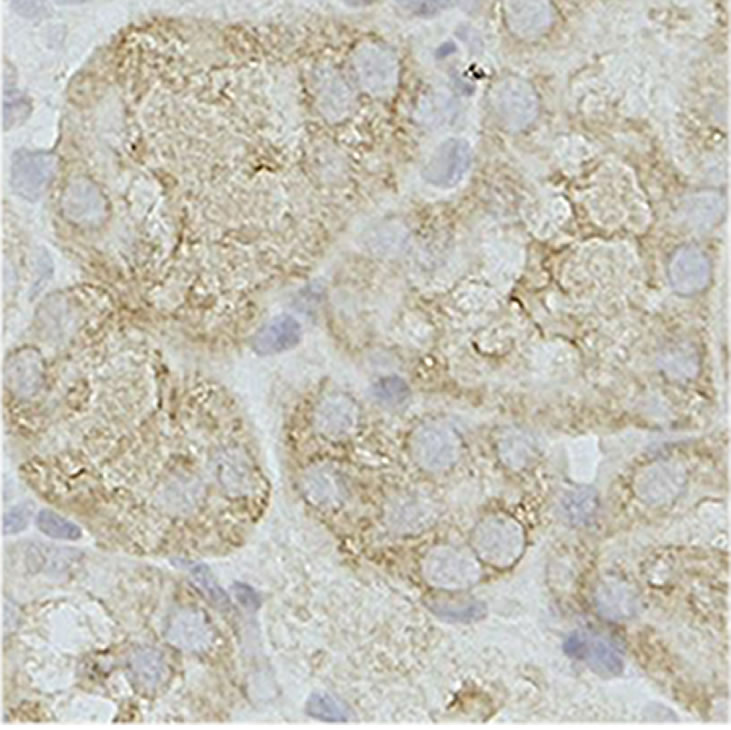

Supplement: Supplementary file 1 [file DataSheet1.zip › Original materials/Microscopic image/Fig 3/MMP7/AAN-8d 2.jpg]

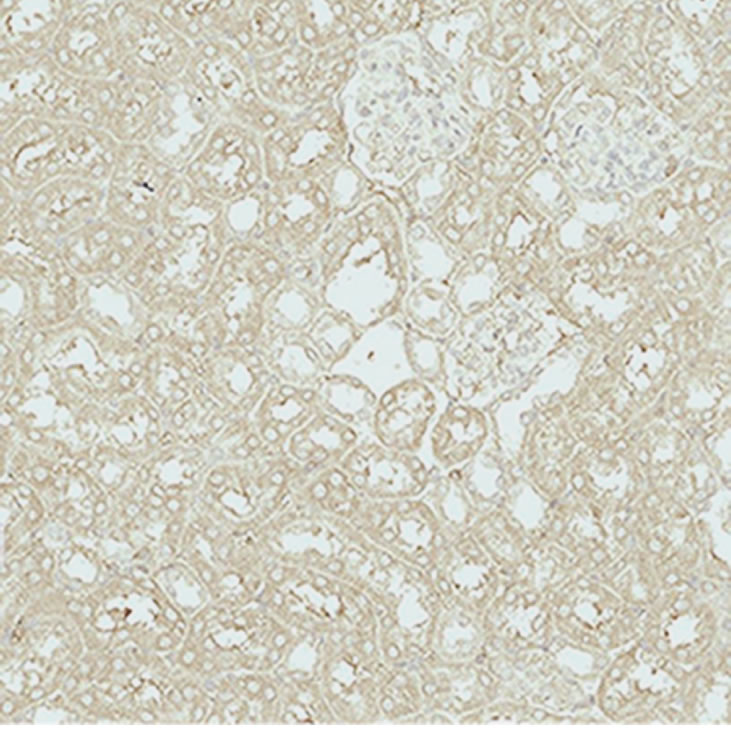

Supplement: Supplementary file 1 [file DataSheet1.zip › Original materials/Microscopic image/Fig 3/MMP7/AAN-Con 1.jpg]

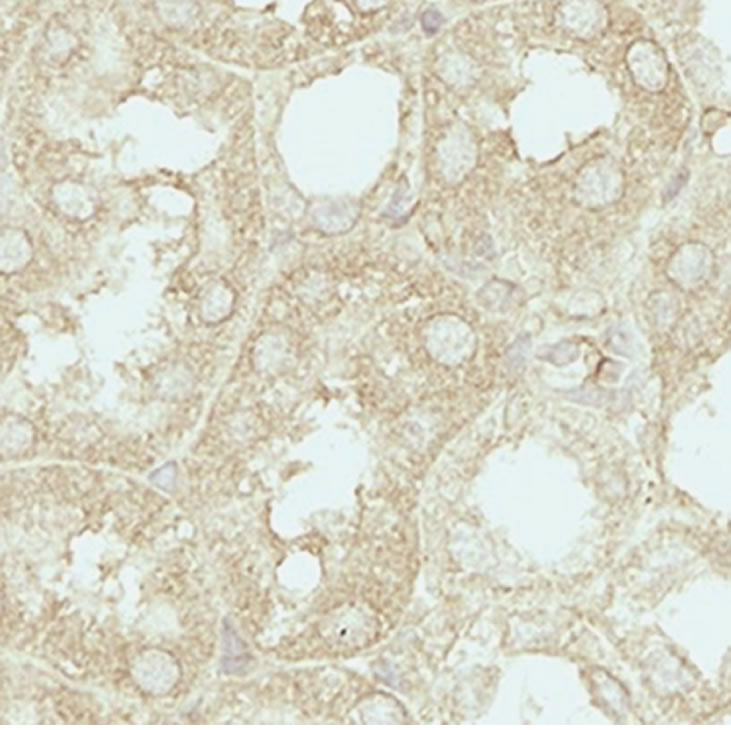

Supplement: Supplementary file 1 [file DataSheet1.zip › Original materials/Microscopic image/Fig 3/MMP7/AAN-Con 2.jpg]

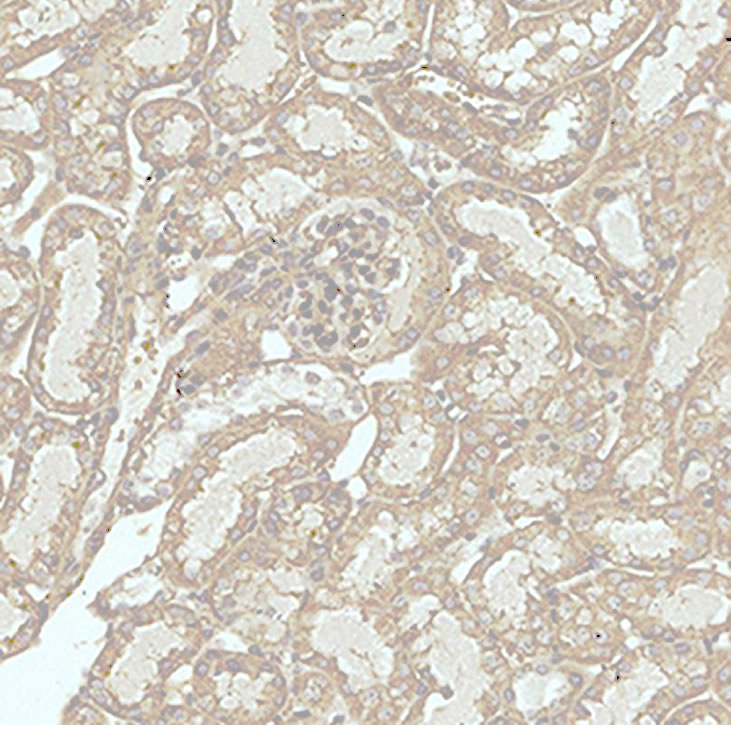

Supplement: Supplementary file 1 [file DataSheet1.zip › Original materials/Microscopic image/Fig 3/Wnt7b/AAN-2d 1.jpg]

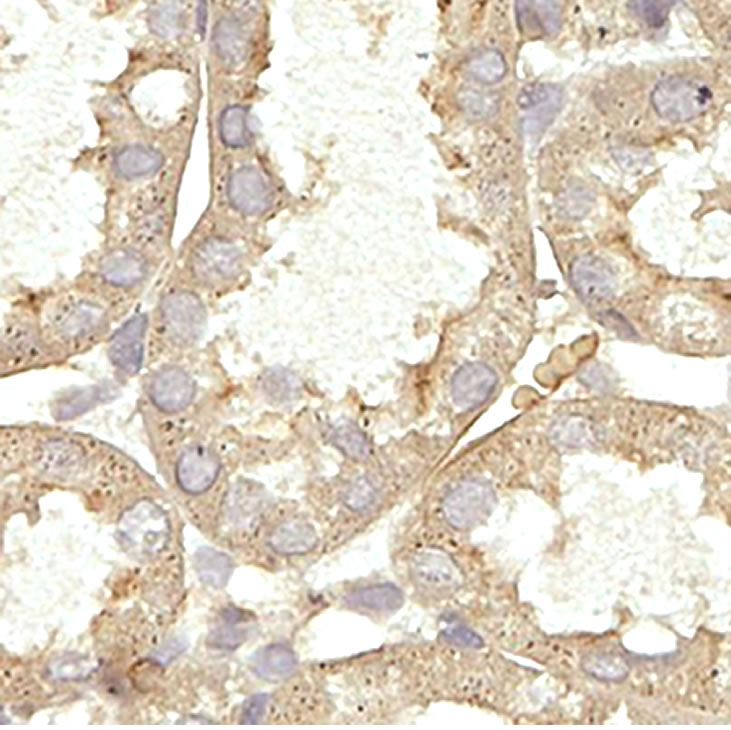

Supplement: Supplementary file 1 [file DataSheet1.zip › Original materials/Microscopic image/Fig 3/Wnt7b/AAN-2d 2.jpg]

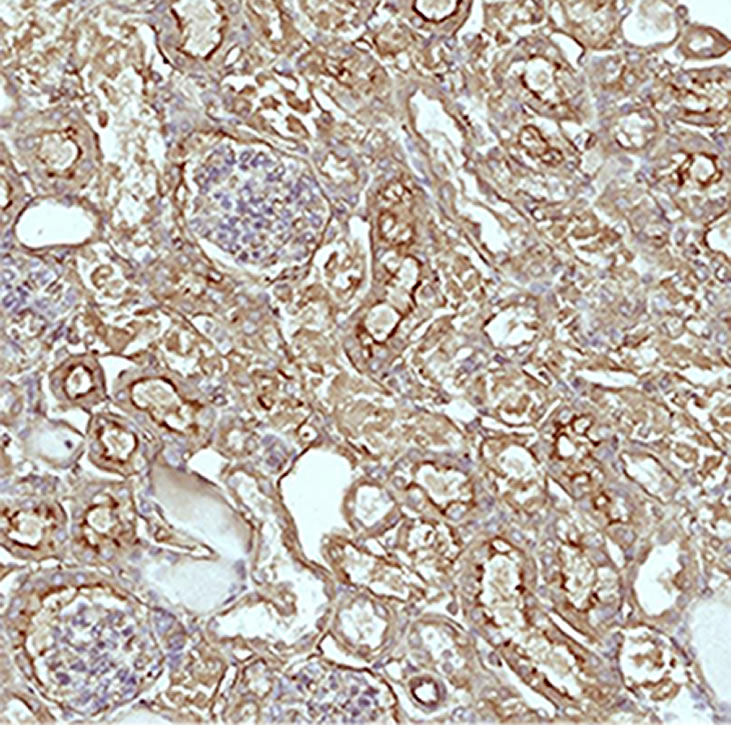

Supplement: Supplementary file 1 [file DataSheet1.zip › Original materials/Microscopic image/Fig 3/Wnt7b/AAN-4d 1.jpg]

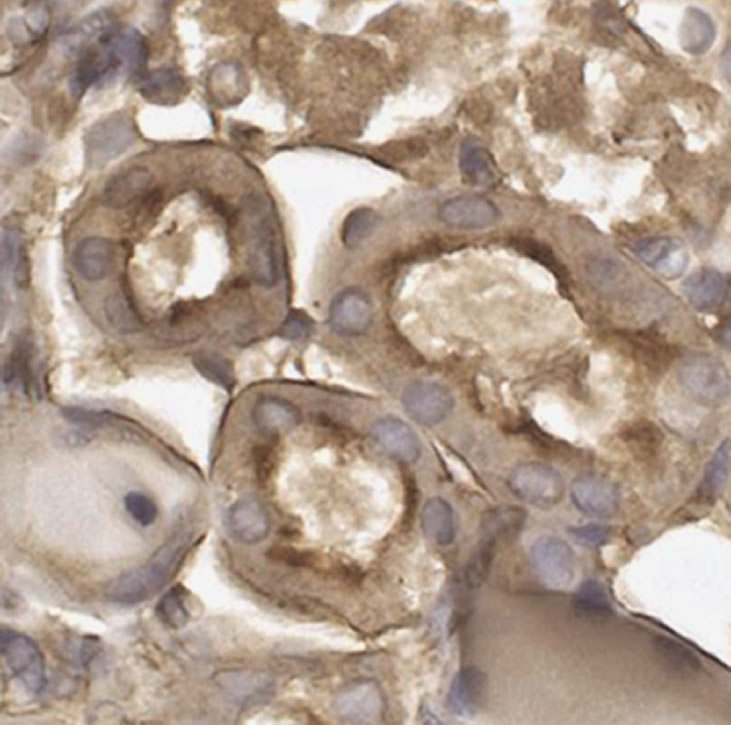

Supplement: Supplementary file 1 [file DataSheet1.zip › Original materials/Microscopic image/Fig 3/Wnt7b/AAN-4d 2.jpg]

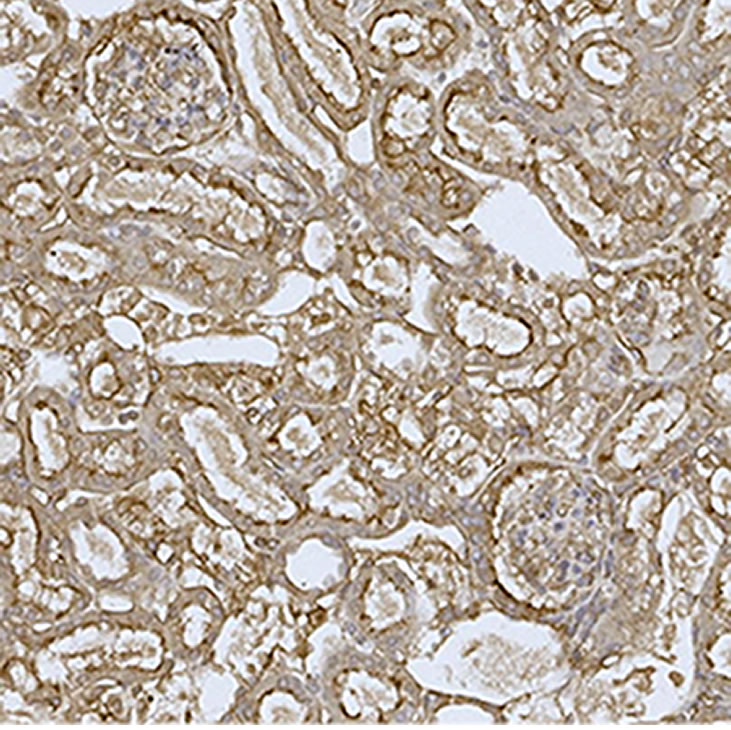

Supplement: Supplementary file 1 [file DataSheet1.zip › Original materials/Microscopic image/Fig 3/Wnt7b/AAN-6d 1.jpg]

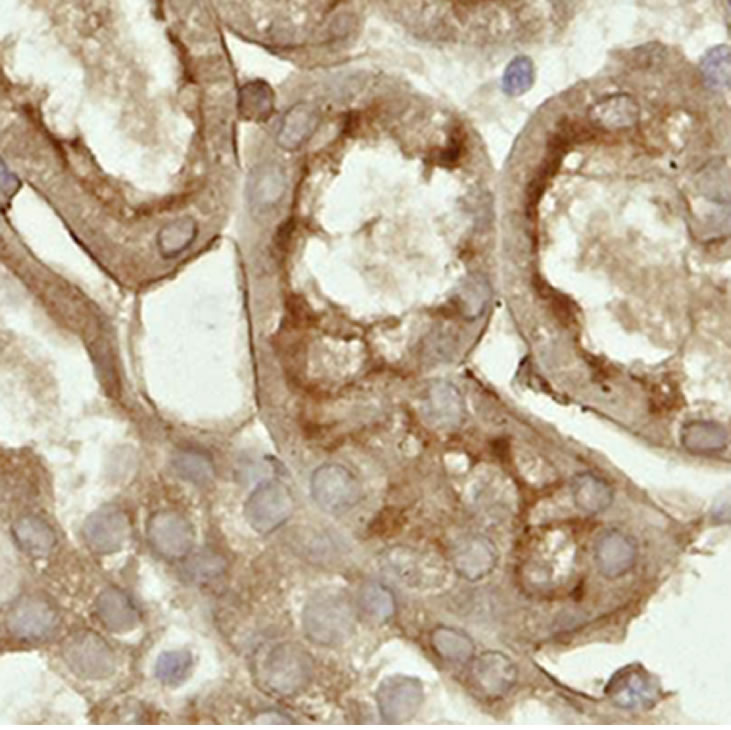

Supplement: Supplementary file 1 [file DataSheet1.zip › Original materials/Microscopic image/Fig 3/Wnt7b/AAN-6d 2.jpg]

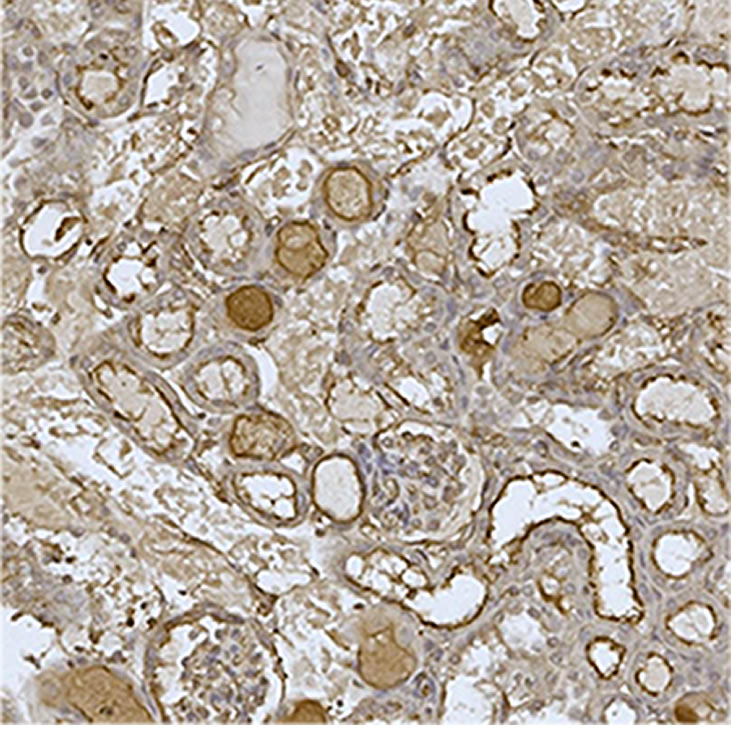

Supplement: Supplementary file 1 [file DataSheet1.zip › Original materials/Microscopic image/Fig 3/Wnt7b/AAN-8d 1.jpg]

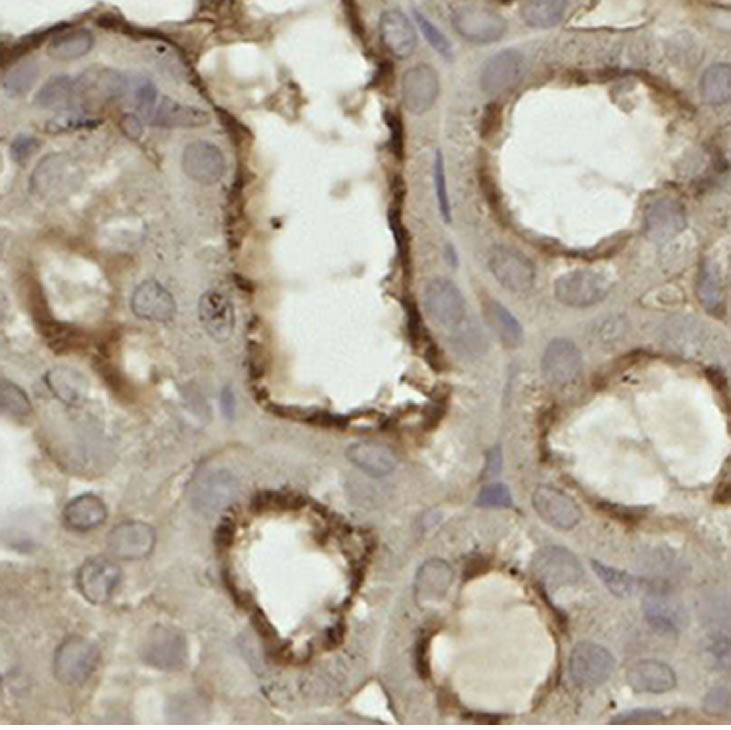

Supplement: Supplementary file 1 [file DataSheet1.zip › Original materials/Microscopic image/Fig 3/Wnt7b/AAN-8d 2.jpg]

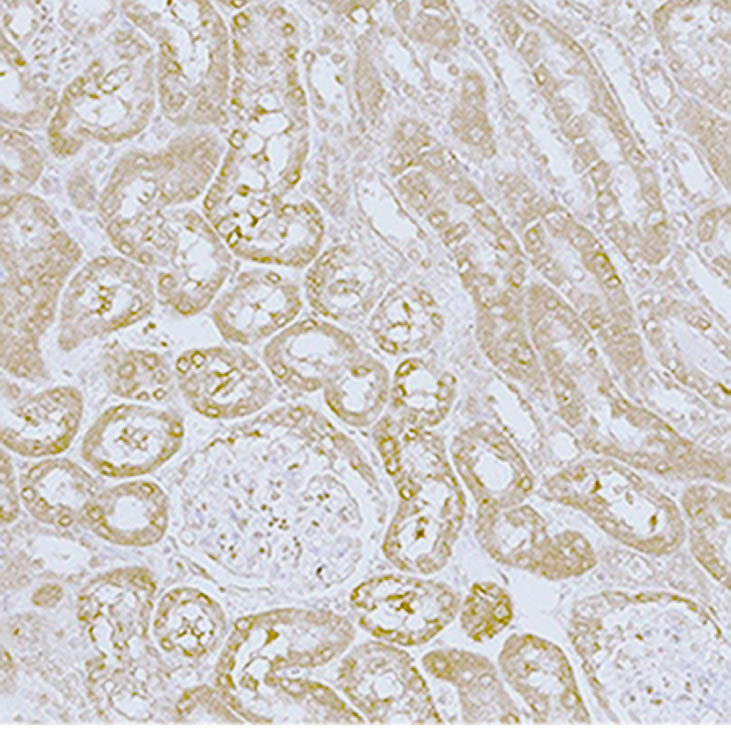

Supplement: Supplementary file 1 [file DataSheet1.zip › Original materials/Microscopic image/Fig 3/Wnt7b/AAN-Con 1.jpg]

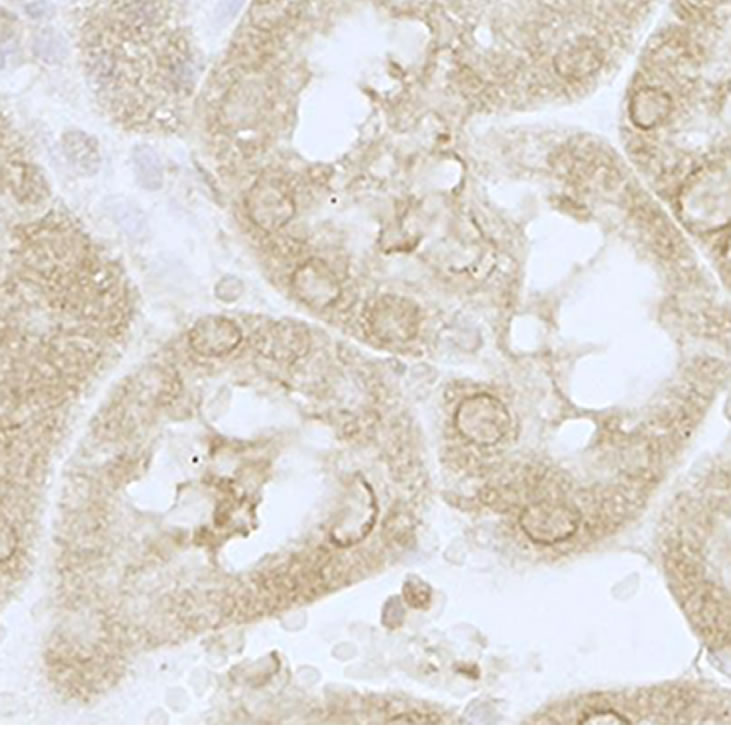

Supplement: Supplementary file 1 [file DataSheet1.zip › Original materials/Microscopic image/Fig 3/Wnt7b/AAN-Con 2.jpg]

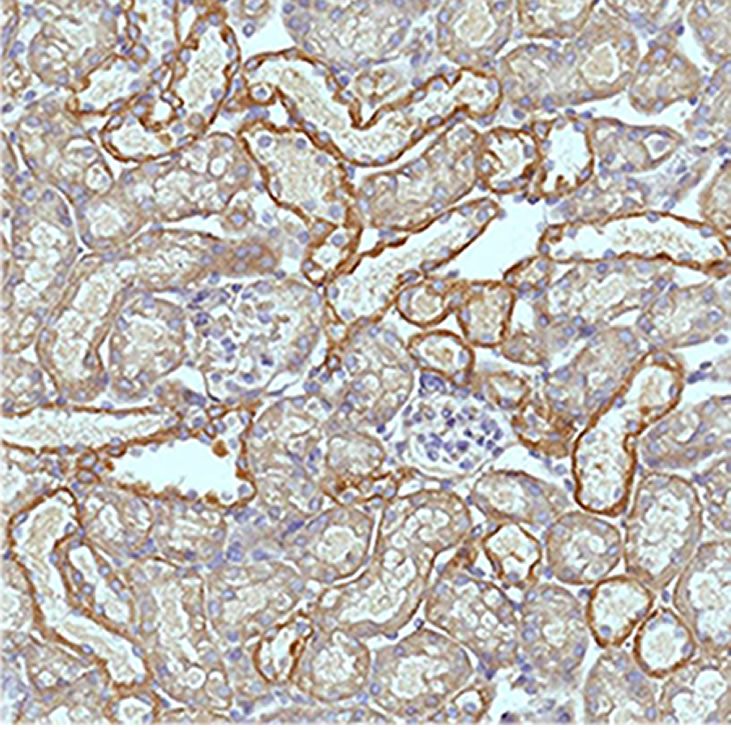

Supplement: Supplementary file 1 [file DataSheet1.zip › Original materials/Microscopic image/Fig 3/β-catenin/AAN-2d 1.jpg]

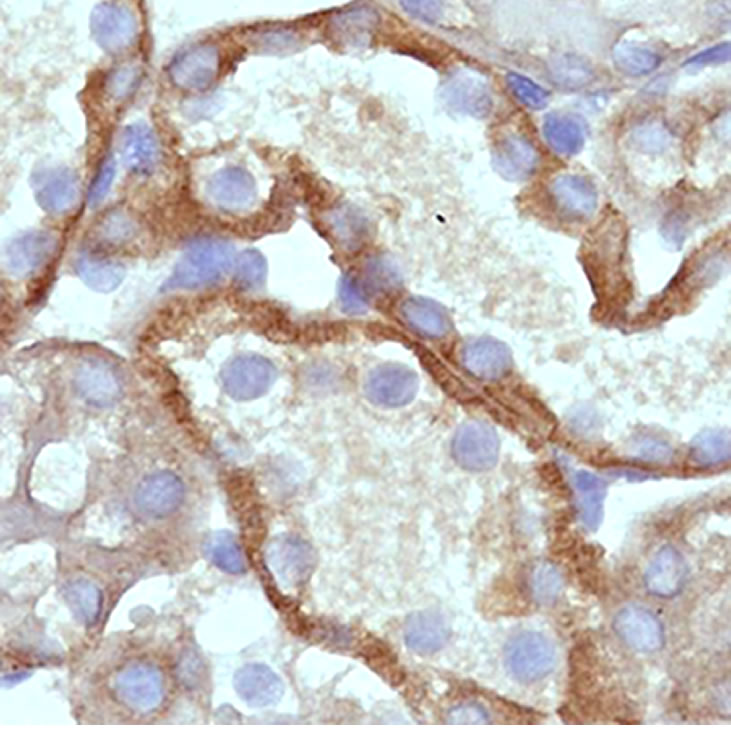

Supplement: Supplementary file 1 [file DataSheet1.zip › Original materials/Microscopic image/Fig 3/β-catenin/AAN-2d 2.jpg]

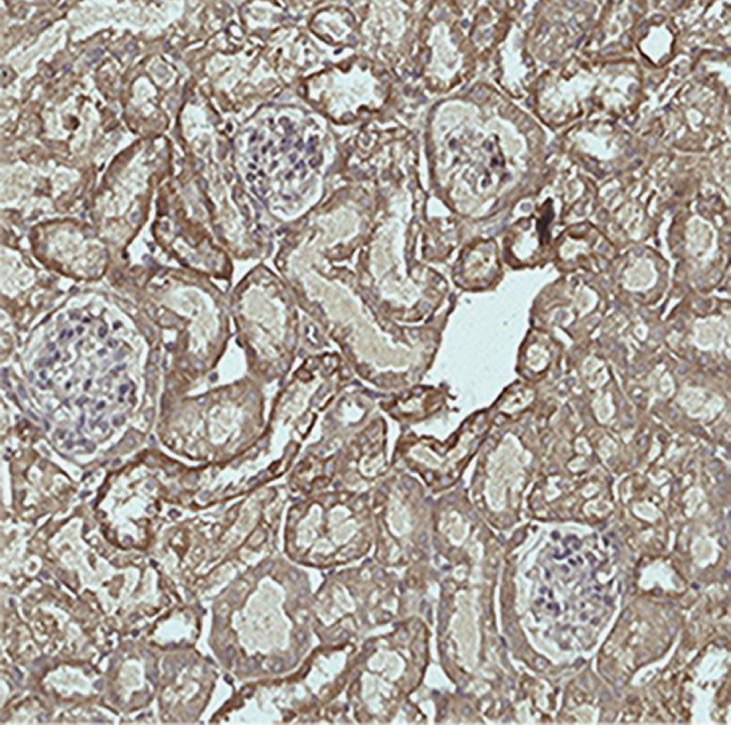

Supplement: Supplementary file 1 [file DataSheet1.zip › Original materials/Microscopic image/Fig 3/β-catenin/AAN-4d 1.jpg]

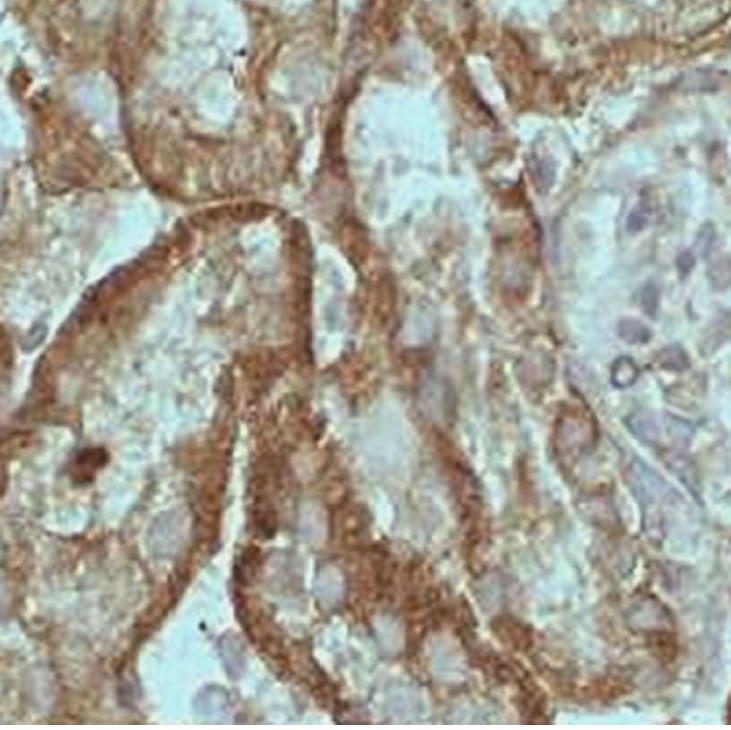

Supplement: Supplementary file 1 [file DataSheet1.zip › Original materials/Microscopic image/Fig 3/β-catenin/AAN-4d 2.jpg]

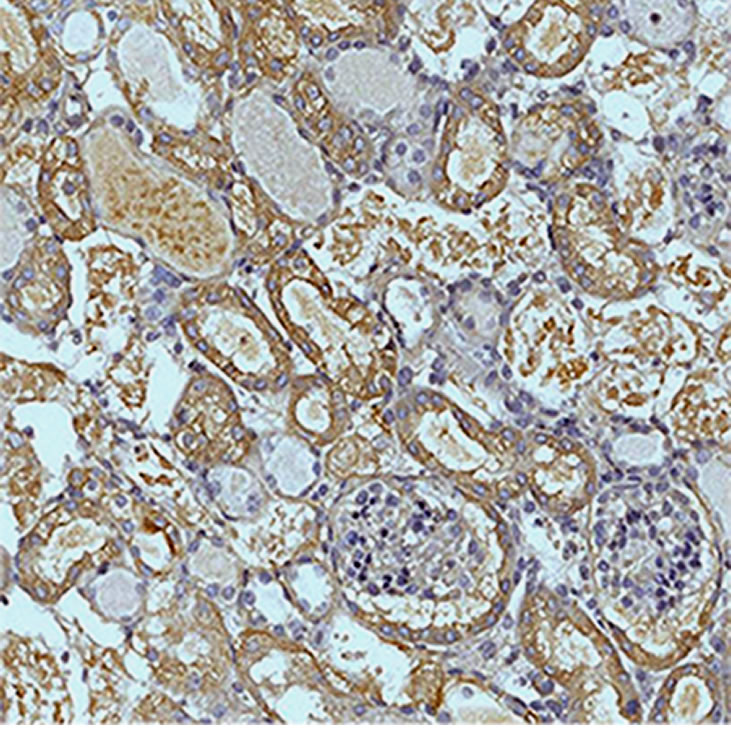

Supplement: Supplementary file 1 [file DataSheet1.zip › Original materials/Microscopic image/Fig 3/β-catenin/AAN-6d 1.jpg]

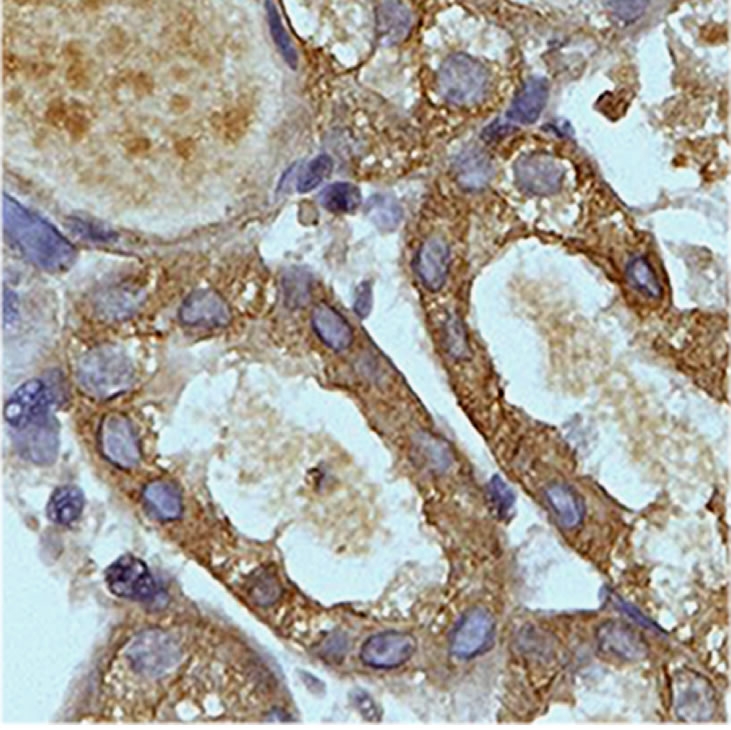

Supplement: Supplementary file 1 [file DataSheet1.zip › Original materials/Microscopic image/Fig 3/β-catenin/AAN-6d 2.jpg]

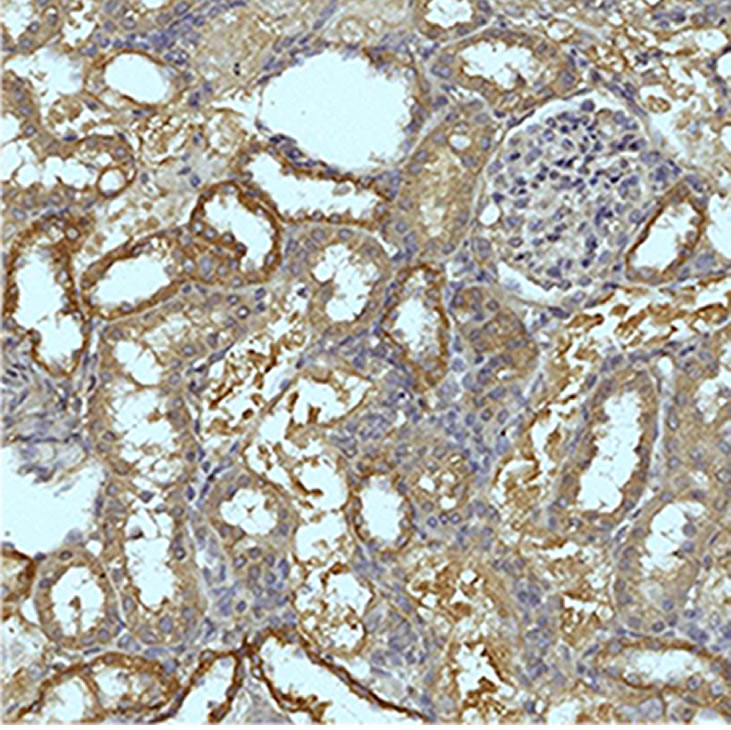

Supplement: Supplementary file 1 [file DataSheet1.zip › Original materials/Microscopic image/Fig 3/β-catenin/AAN-8d 1.jpg]

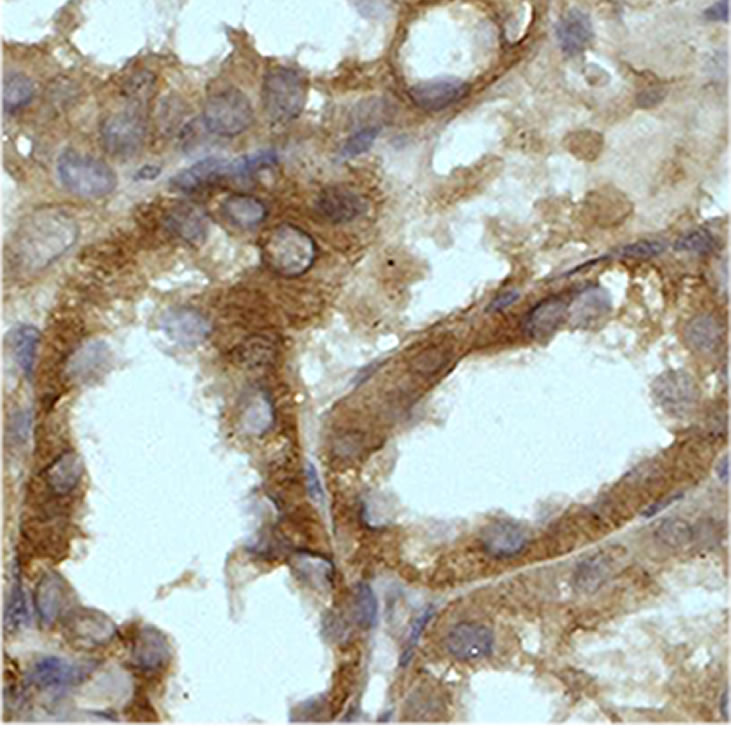

Supplement: Supplementary file 1 [file DataSheet1.zip › Original materials/Microscopic image/Fig 3/β-catenin/AAN-8d 2.jpg]

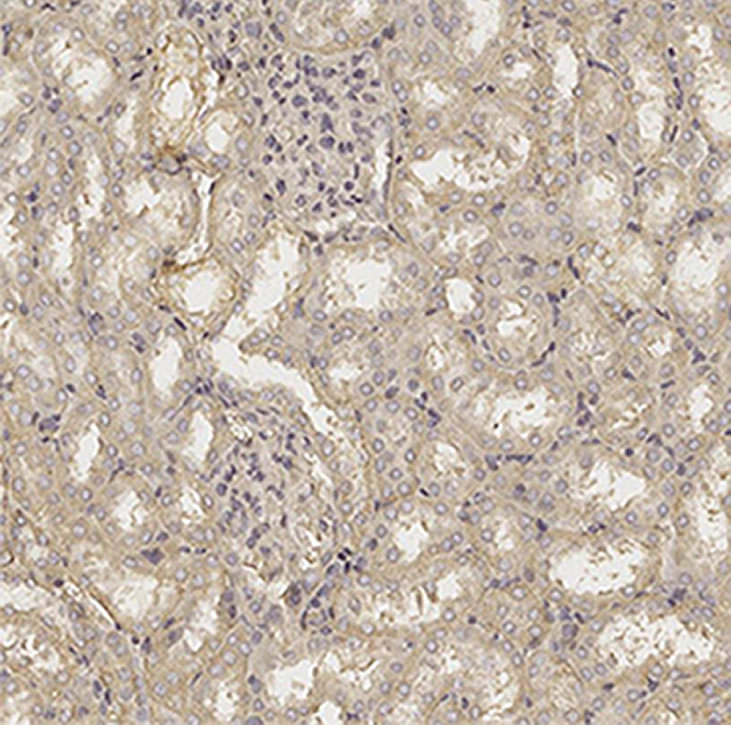

Supplement: Supplementary file 1 [file DataSheet1.zip › Original materials/Microscopic image/Fig 3/β-catenin/AAN-Con 1.jpg]

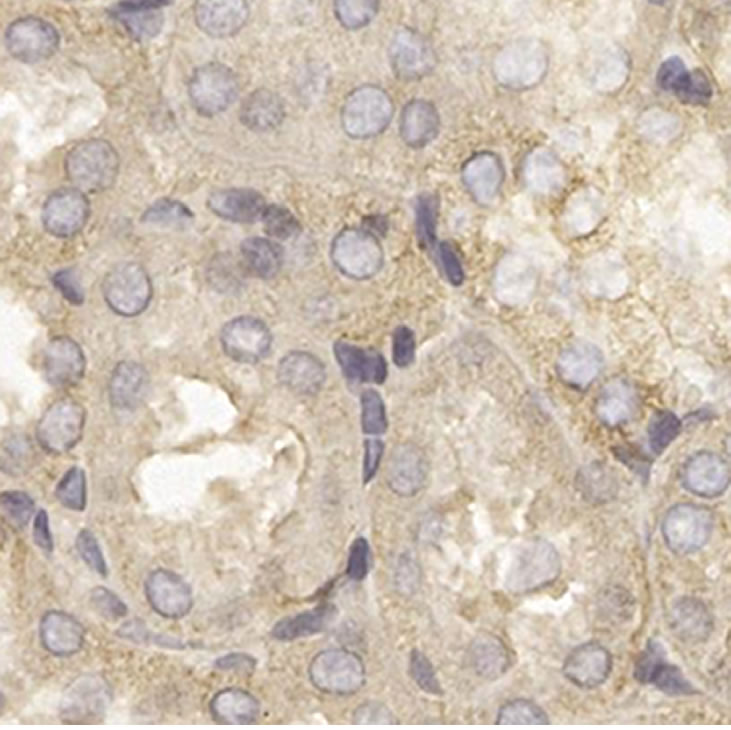

Supplement: Supplementary file 1 [file DataSheet1.zip › Original materials/Microscopic image/Fig 3/β-catenin/AAN-Con 2.jpg]

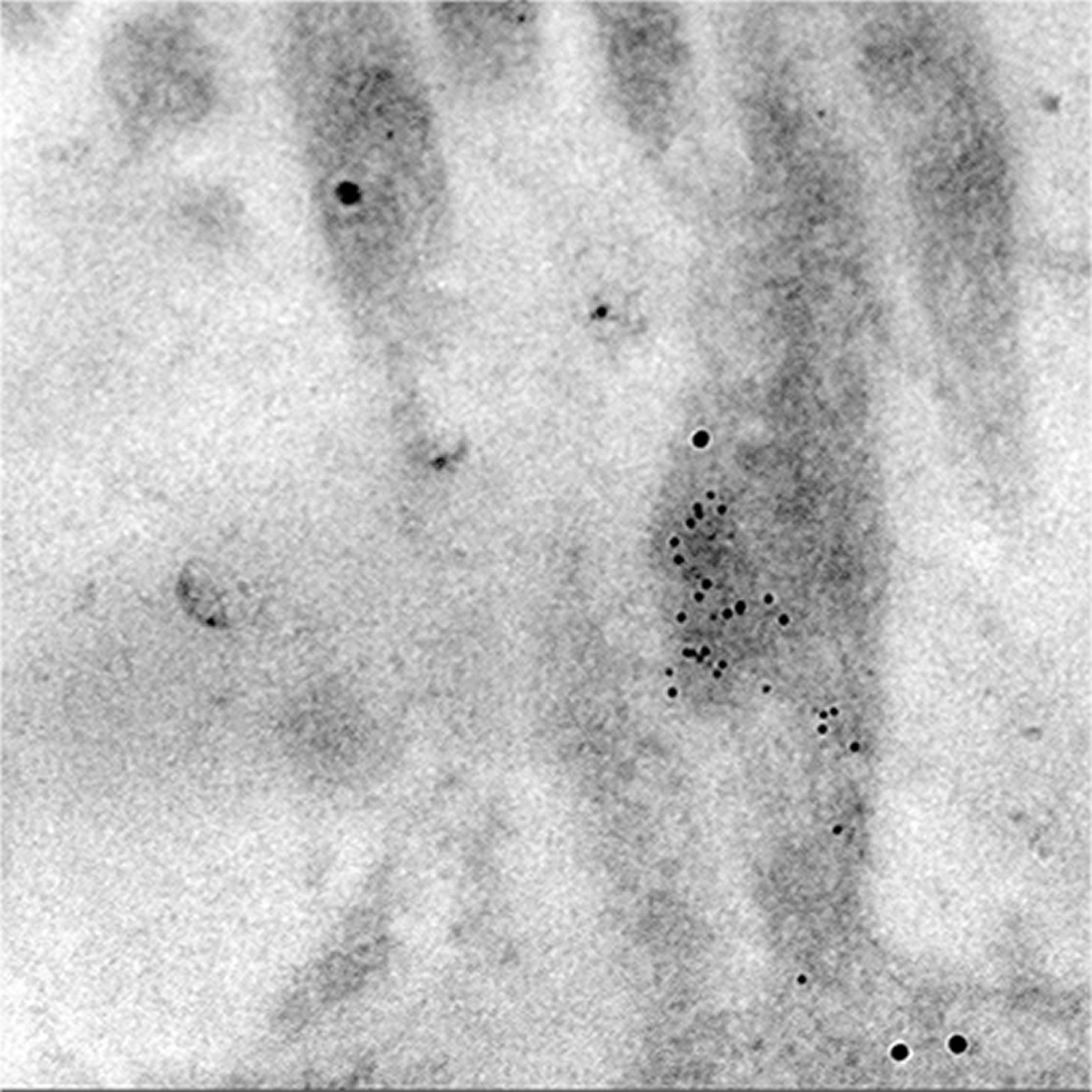

Supplement: Supplementary file 1 [file DataSheet1.zip › Original materials/Microscopic image/Fig 4/AAN-2d brush border.jpg]

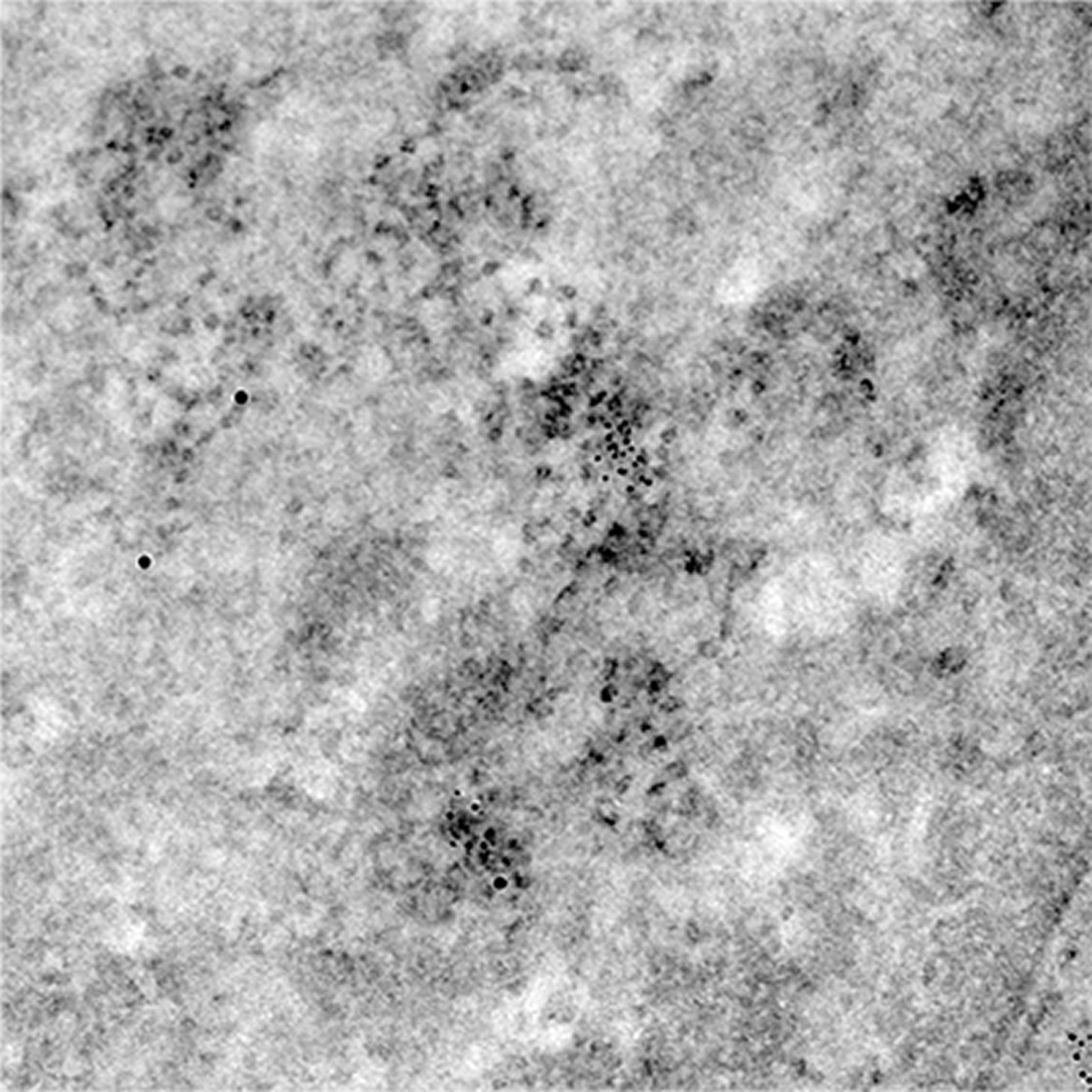

Supplement: Supplementary file 1 [file DataSheet1.zip › Original materials/Microscopic image/Fig 4/AAN-2d intranuclear.jpg]

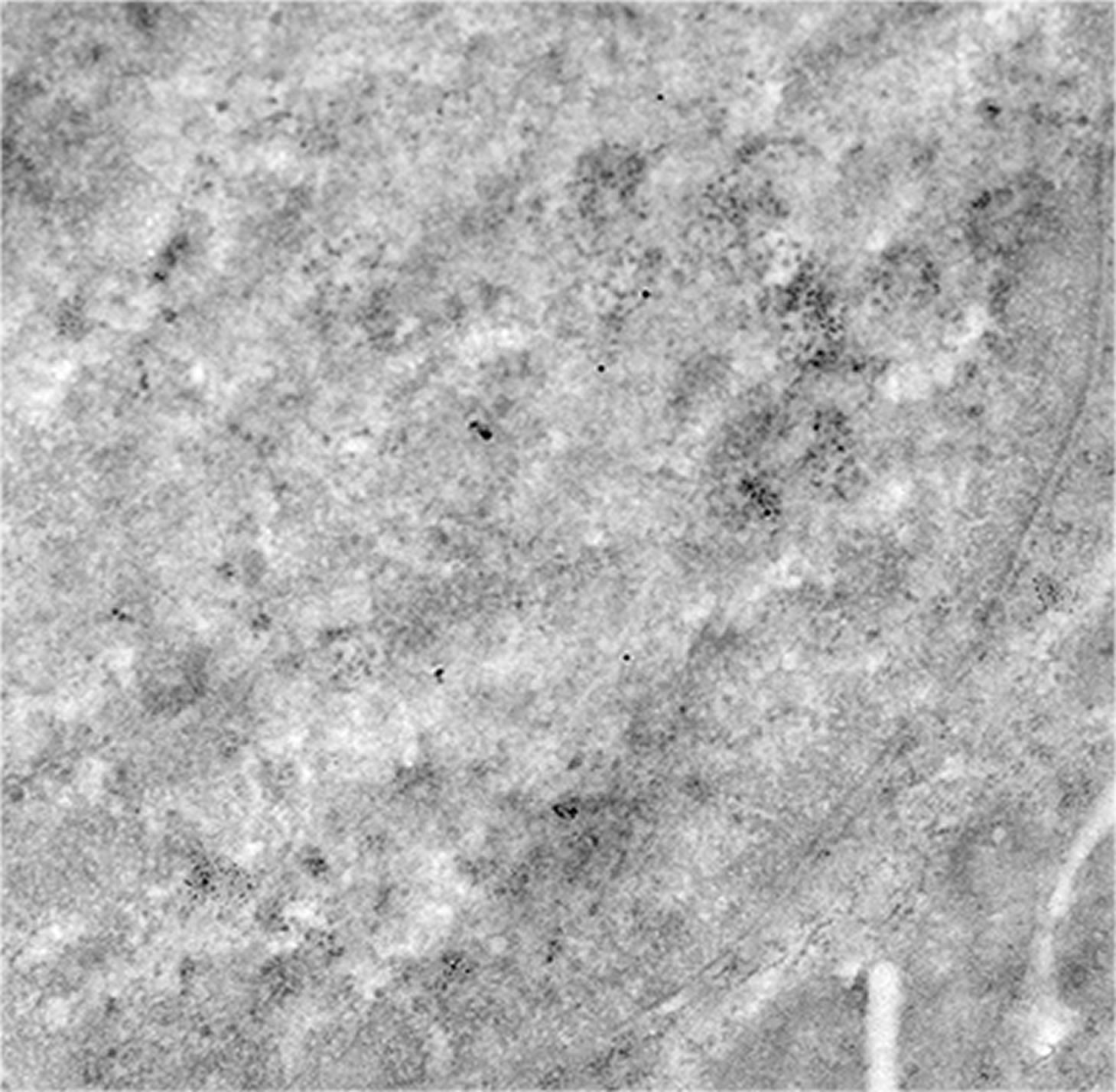

Supplement: Supplementary file 1 [file DataSheet1.zip › Original materials/Microscopic image/Fig 4/AAN-2d nucleus.jpg]

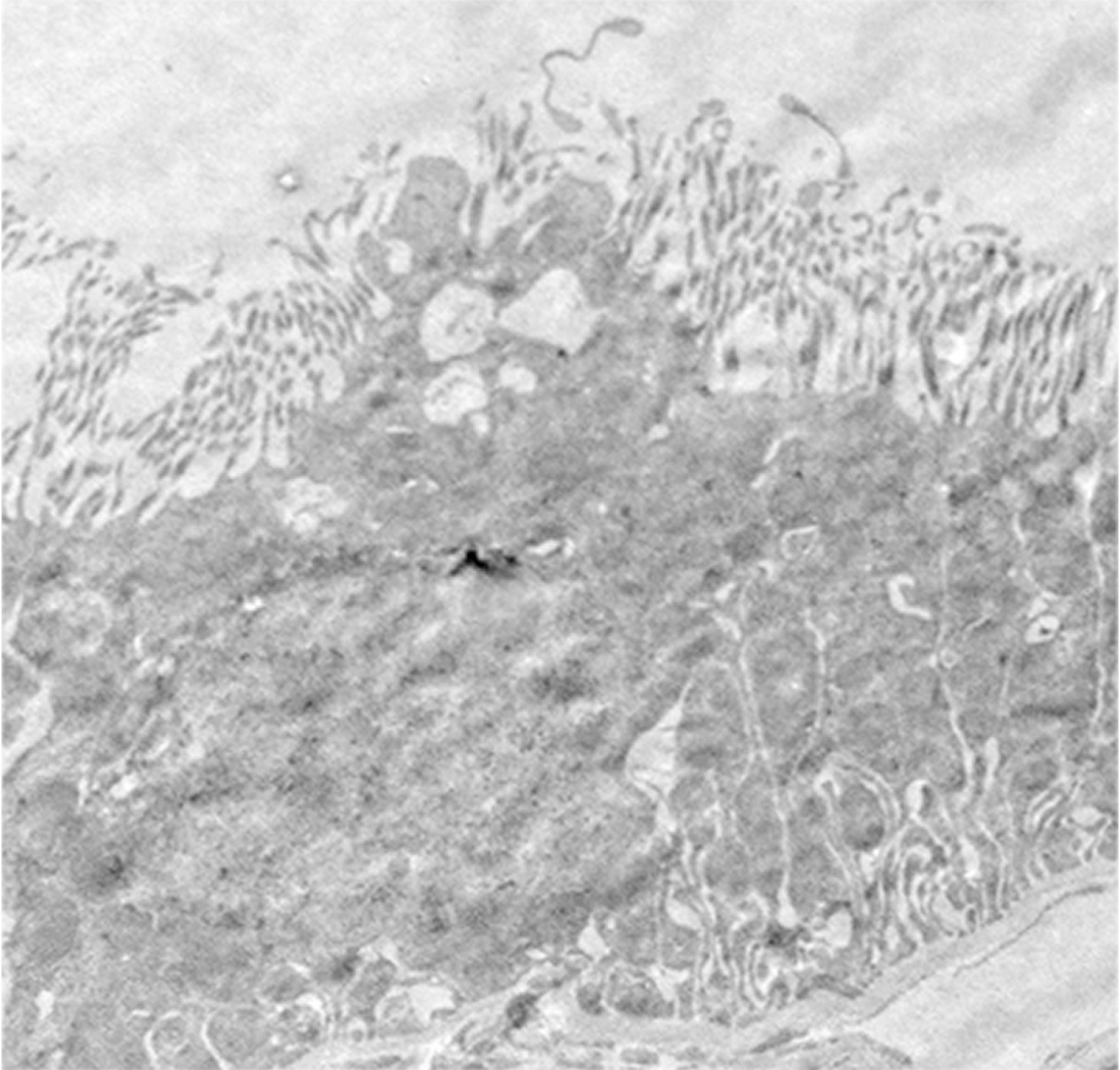

Supplement: Supplementary file 1 [file DataSheet1.zip › Original materials/Microscopic image/Fig 4/AAN-2d PCTEC.jpg]

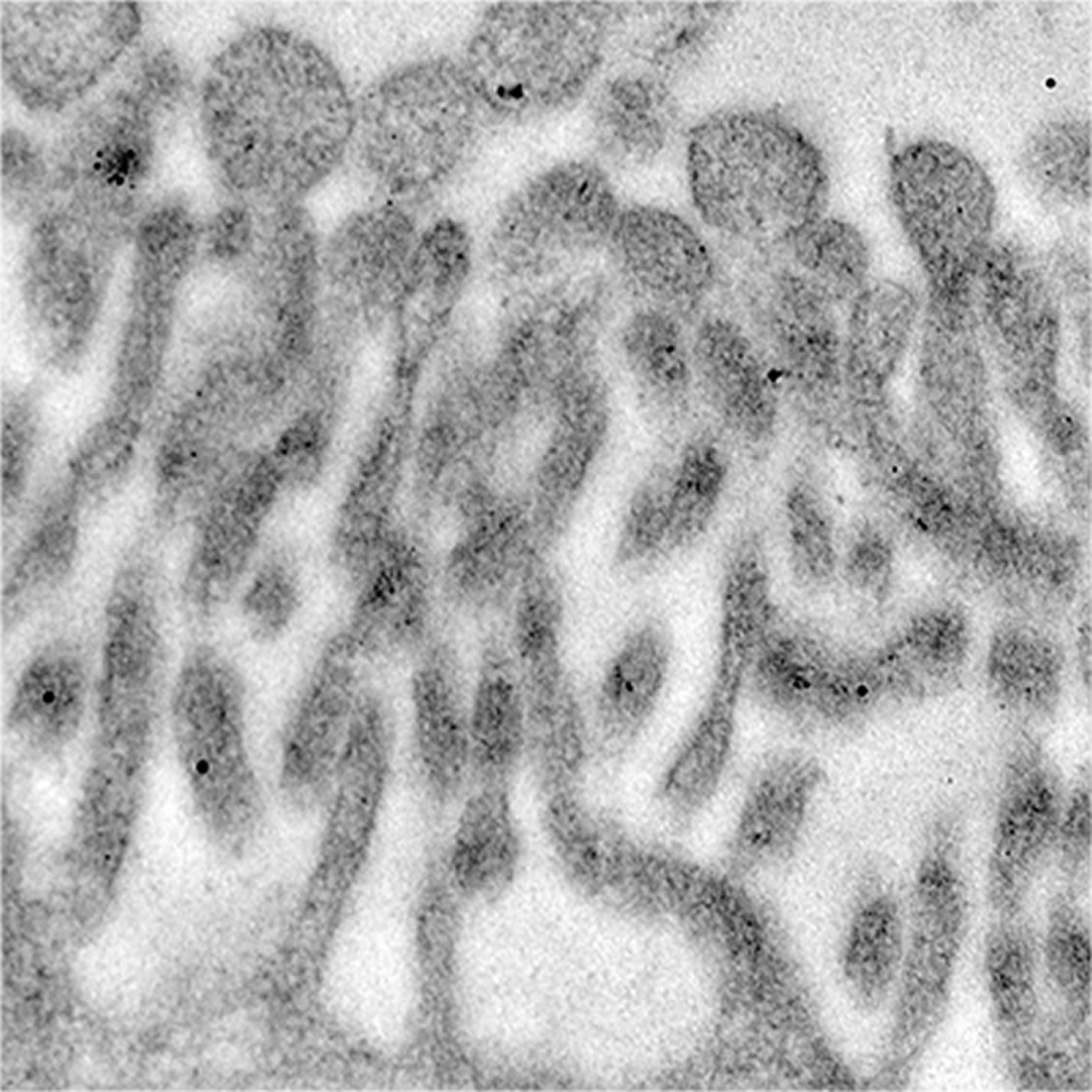

Supplement: Supplementary file 1 [file DataSheet1.zip › Original materials/Microscopic image/Fig 4/AAN-6d brush border.jpg]

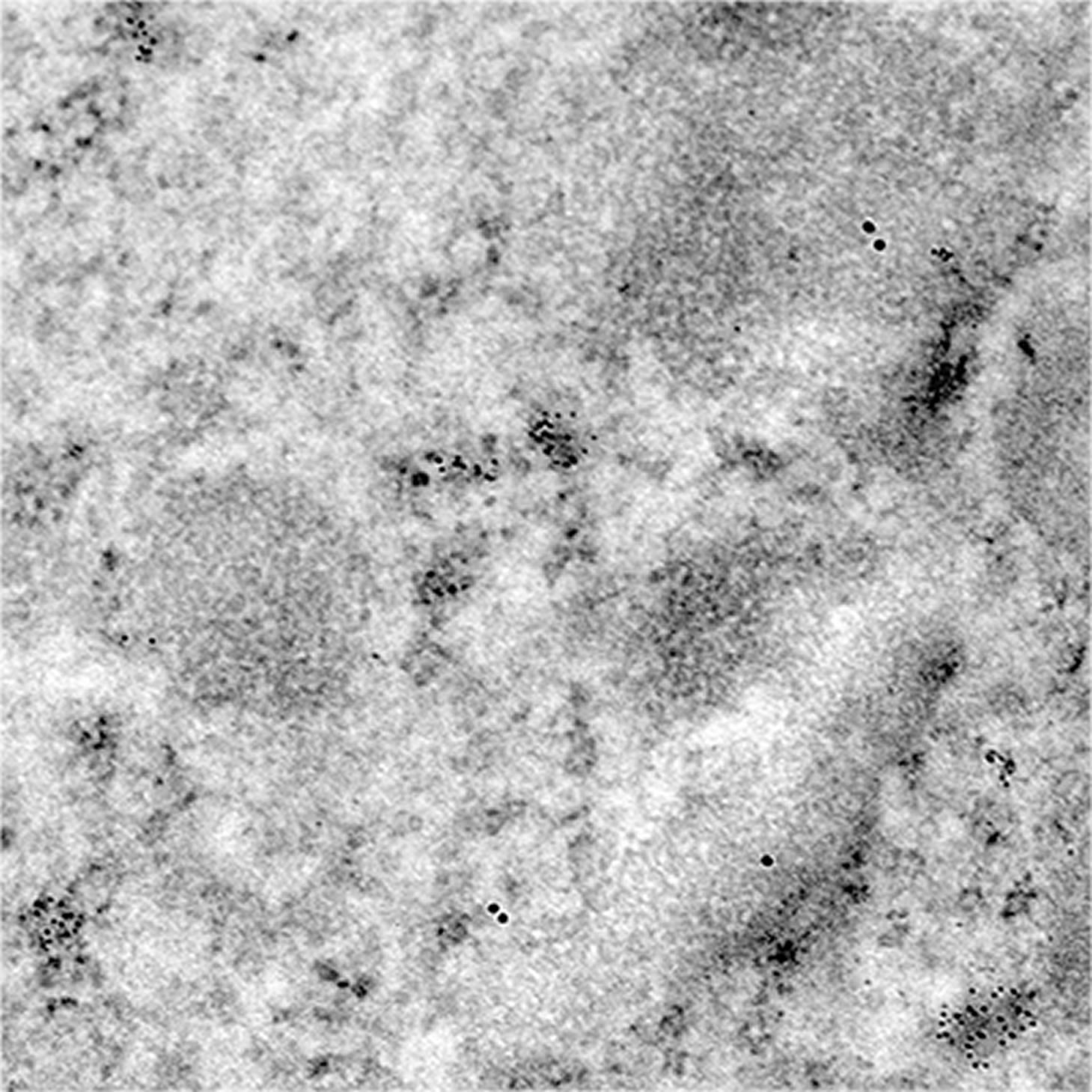

Supplement: Supplementary file 1 [file DataSheet1.zip › Original materials/Microscopic image/Fig 4/AAN-6d intranuclear.jpg]

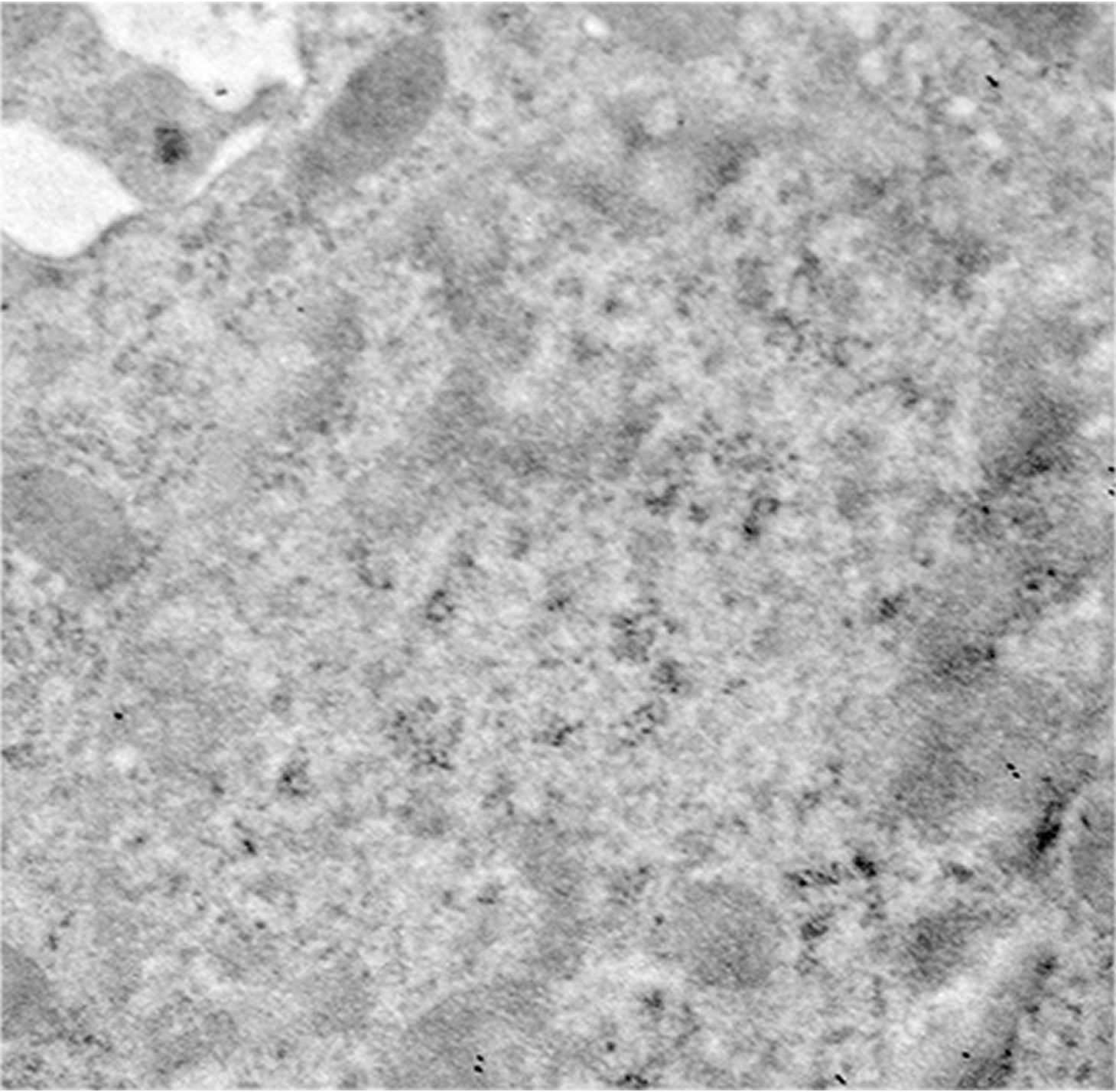

Supplement: Supplementary file 1 [file DataSheet1.zip › Original materials/Microscopic image/Fig 4/AAN-6d nucleus.jpg]

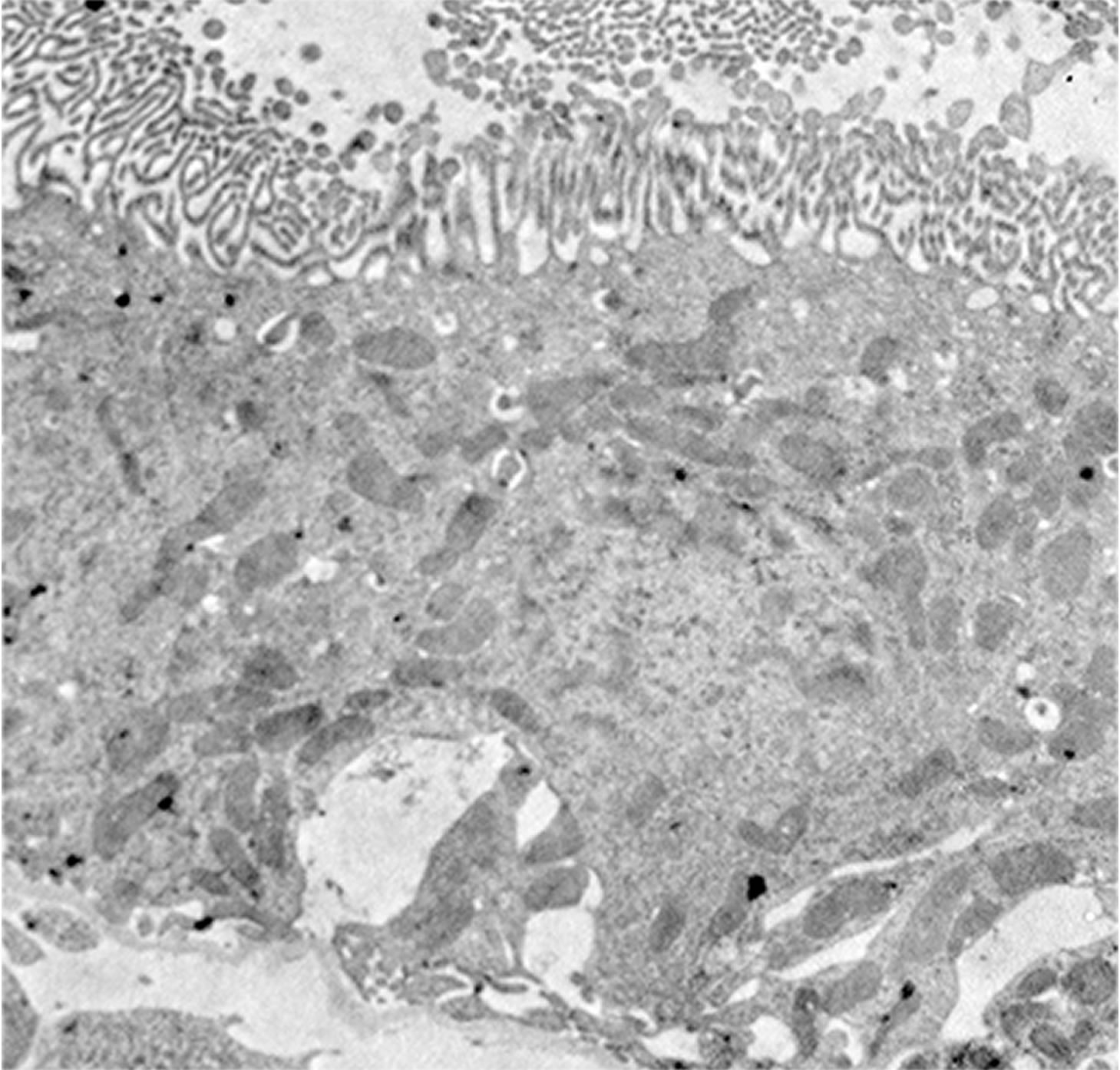

Supplement: Supplementary file 1 [file DataSheet1.zip › Original materials/Microscopic image/Fig 4/AAN-6d PCTEC.jpg]

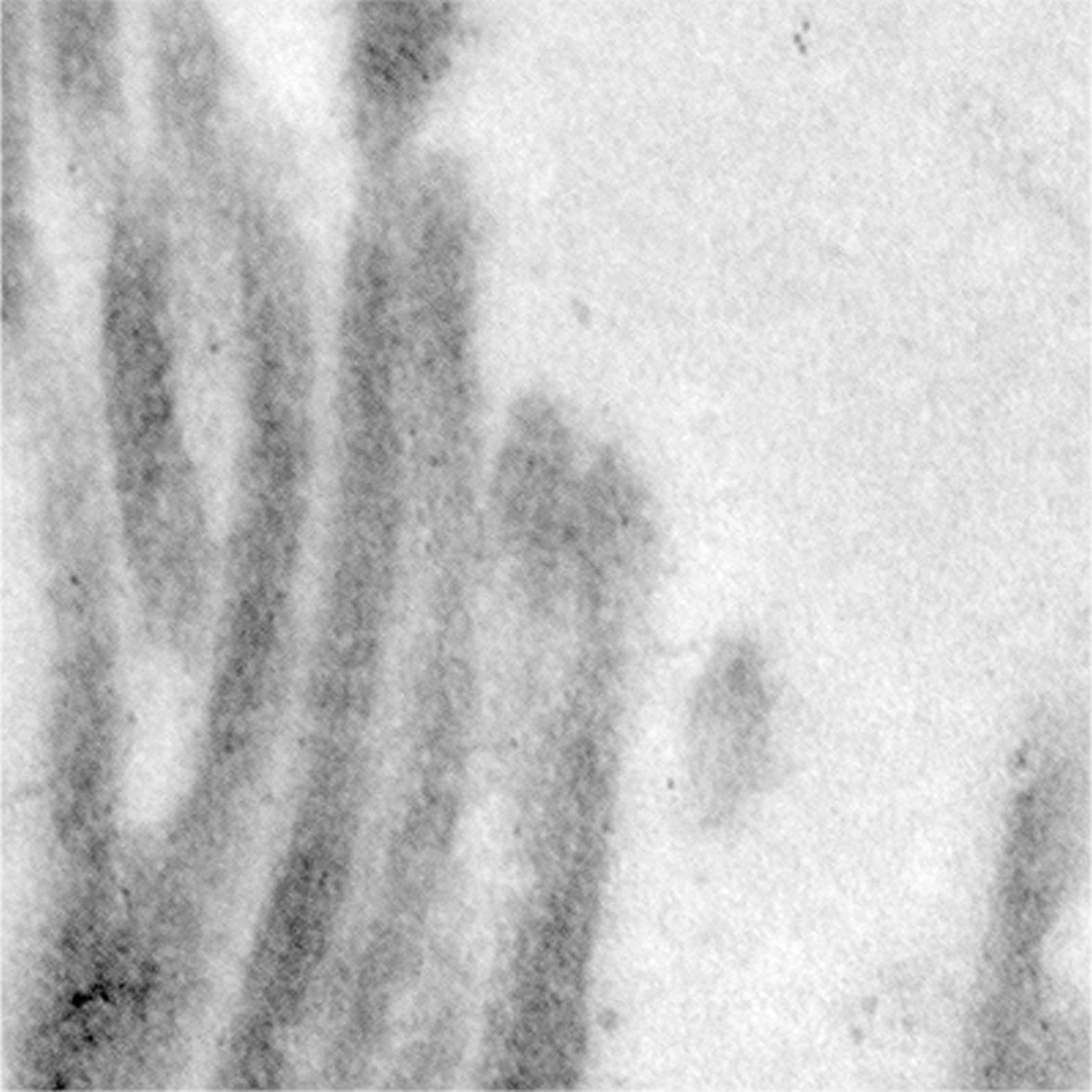

Supplement: Supplementary file 1 [file DataSheet1.zip › Original materials/Microscopic image/Fig 4/AAN-Con brush border.jpg]

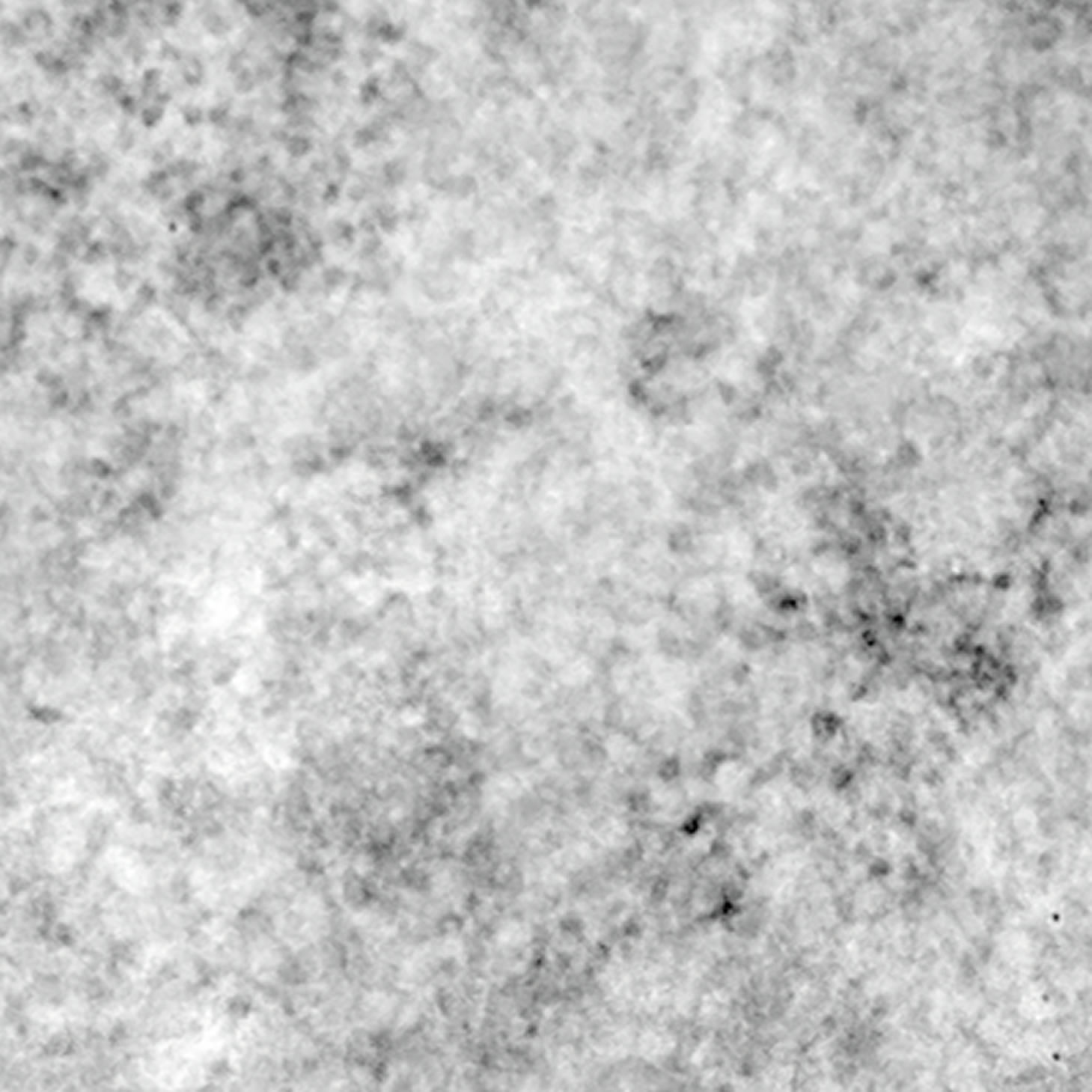

Supplement: Supplementary file 1 [file DataSheet1.zip › Original materials/Microscopic image/Fig 4/AAN-Con intranuclear.jpg]

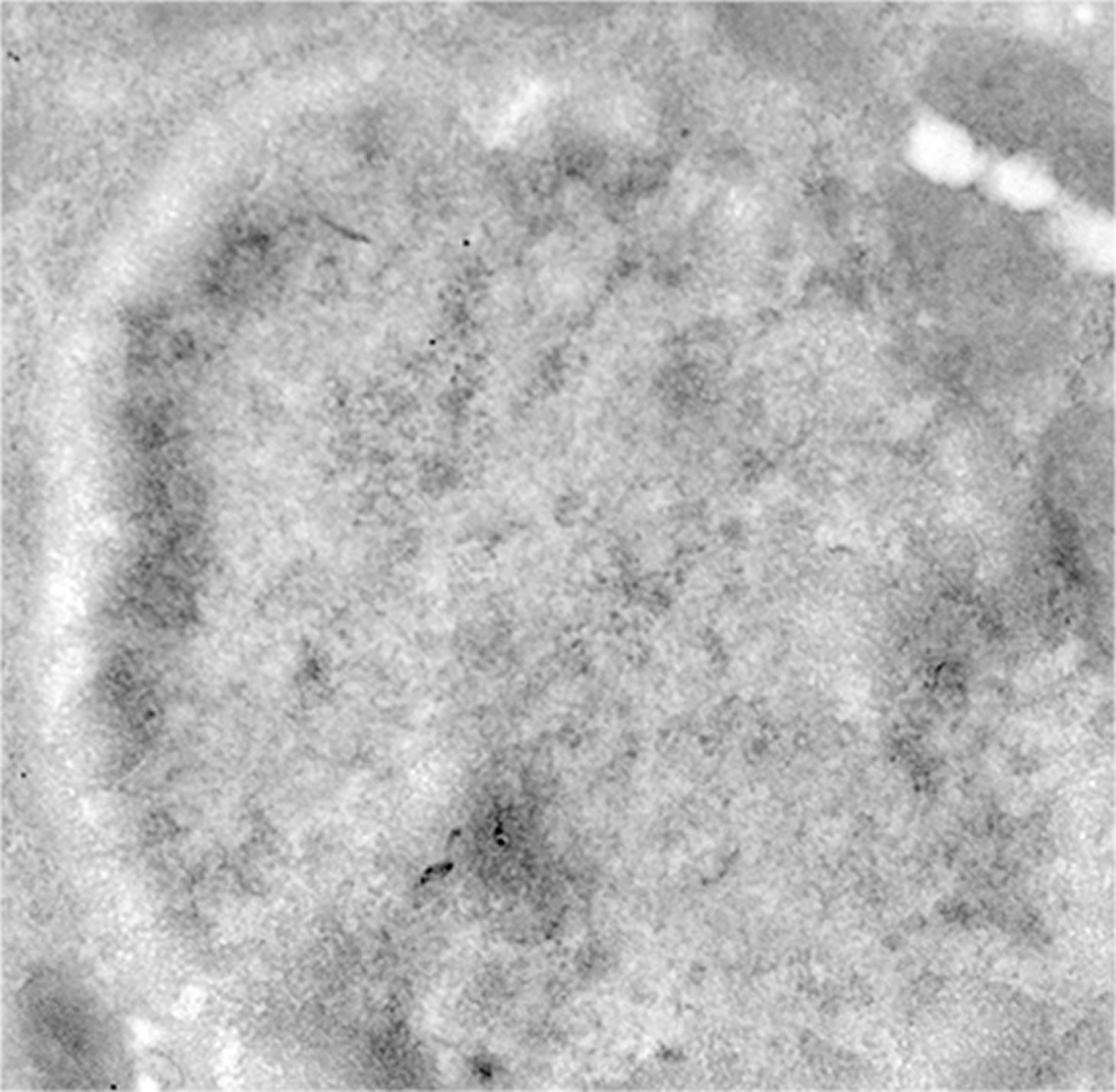

Supplement: Supplementary file 1 [file DataSheet1.zip › Original materials/Microscopic image/Fig 4/AAN-Con nucleus.jpg]

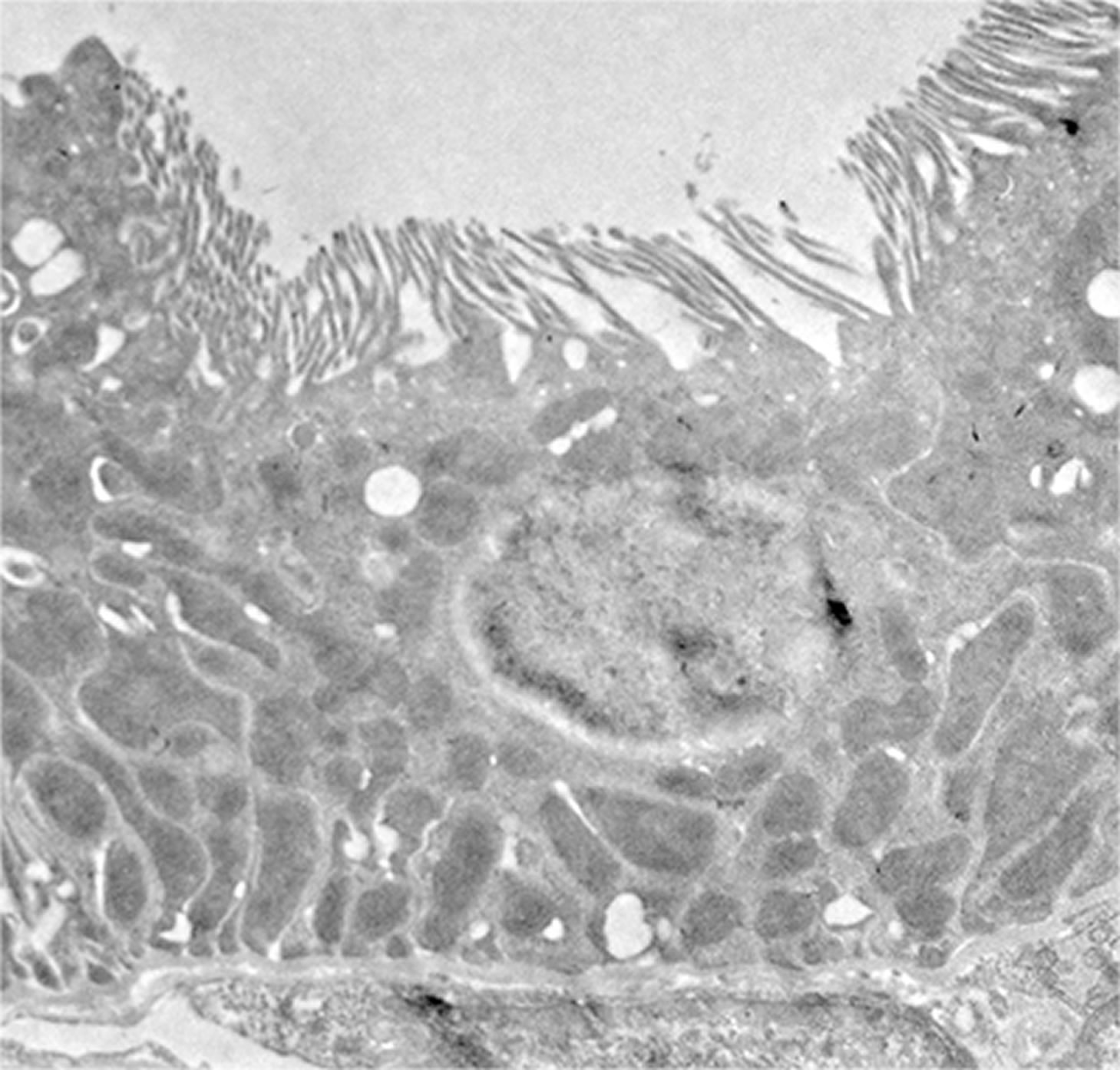

Supplement: Supplementary file 1 [file DataSheet1.zip › Original materials/Microscopic image/Fig 4/AAN-Con PCTEC.jpg]

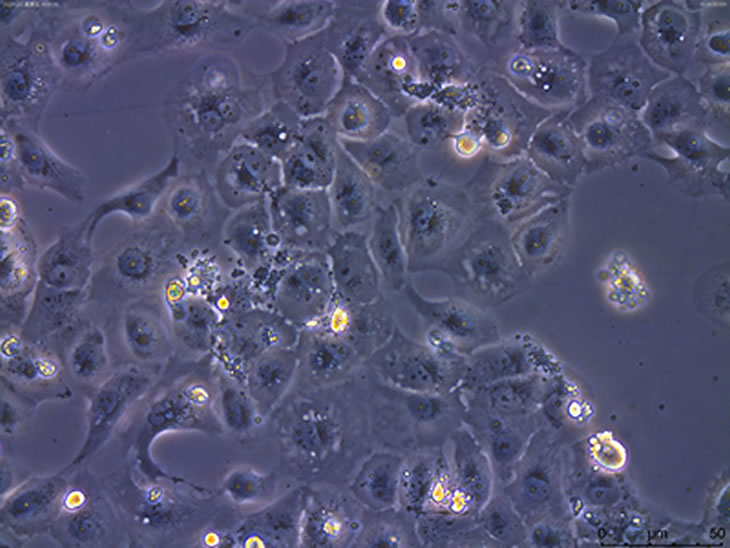

Supplement: Supplementary file 1 [file DataSheet1.zip › Original materials/Microscopic image/Fig 5/100μM AAI.jpg]

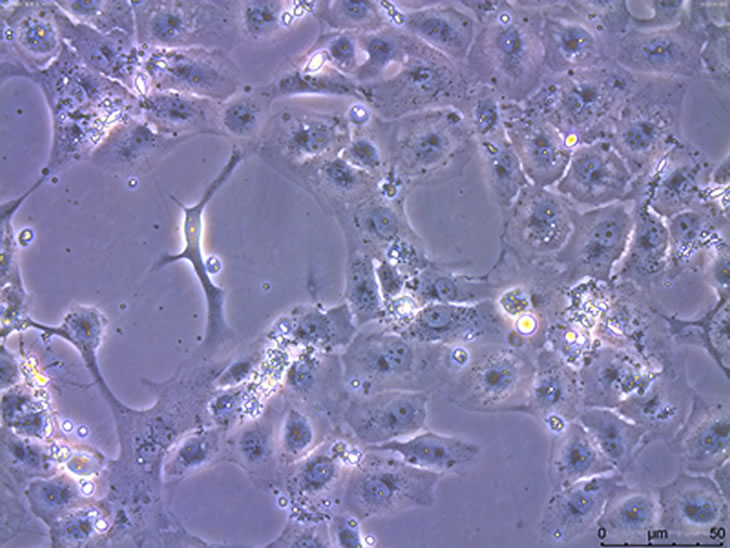

Supplement: Supplementary file 1 [file DataSheet1.zip › Original materials/Microscopic image/Fig 5/200μM AAI.jpg]

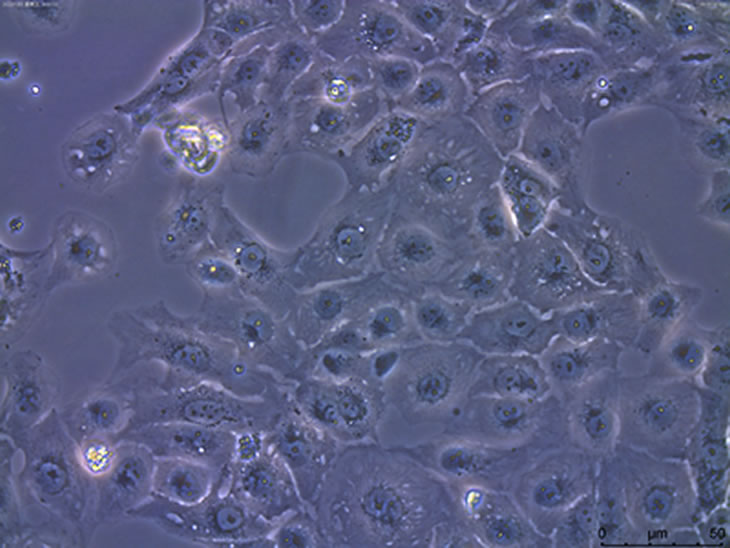

Supplement: Supplementary file 1 [file DataSheet1.zip › Original materials/Microscopic image/Fig 5/50μM AAI.jpg]

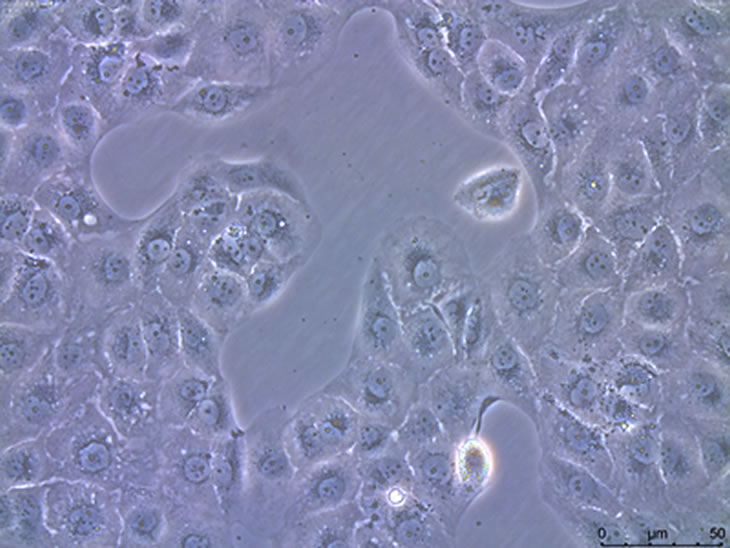

Supplement: Supplementary file 1 [file DataSheet1.zip › Original materials/Microscopic image/Fig 5/Cisplatin.jpg]

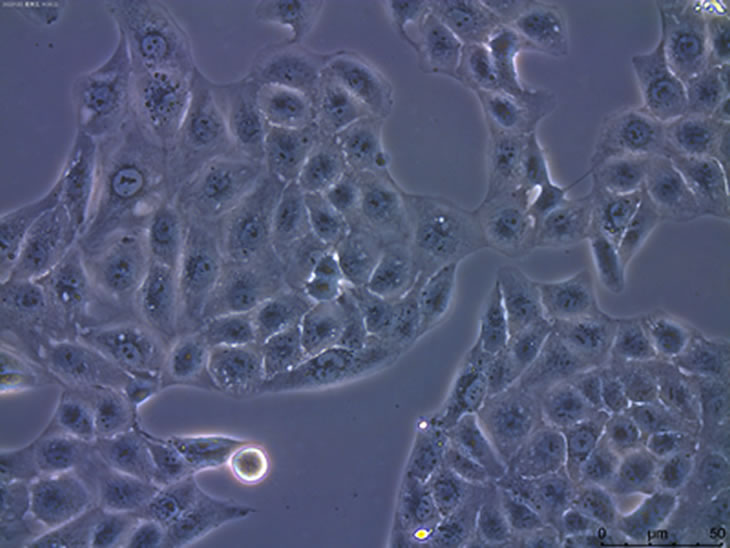

Supplement: Supplementary file 1 [file DataSheet1.zip › Original materials/Microscopic image/Fig 5/Con.jpg]

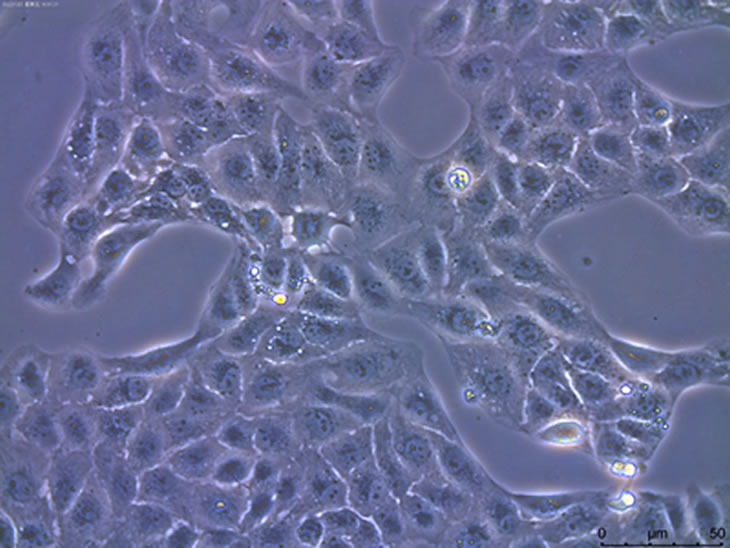

Supplement: Supplementary file 1 [file DataSheet1.zip › Original materials/Microscopic image/Fig 5/DMSO.jpg]

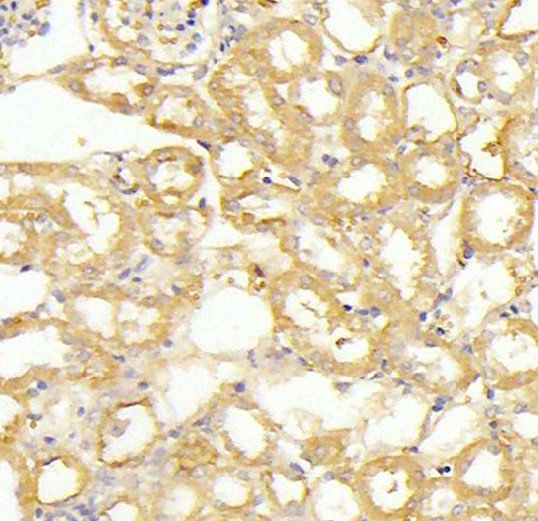

Supplement: Supplementary file 1 [file DataSheet1.zip › Original materials/Microscopic image/Fig 6/AQP1/AAN-4w.jpg]

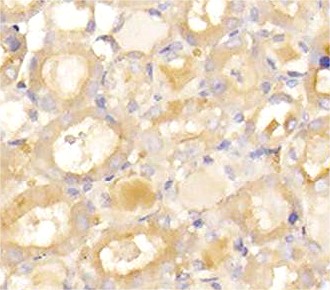

Supplement: Supplementary file 1 [file DataSheet1.zip › Original materials/Microscopic image/Fig 6/AQP1/AAN-8d.jpg]

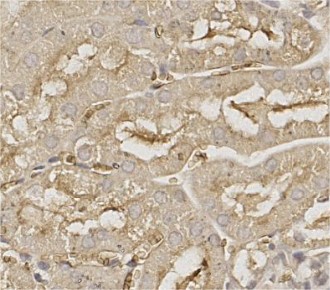

Supplement: Supplementary file 1 [file DataSheet1.zip › Original materials/Microscopic image/Fig 6/AQP1/AAN-Con.jpg]

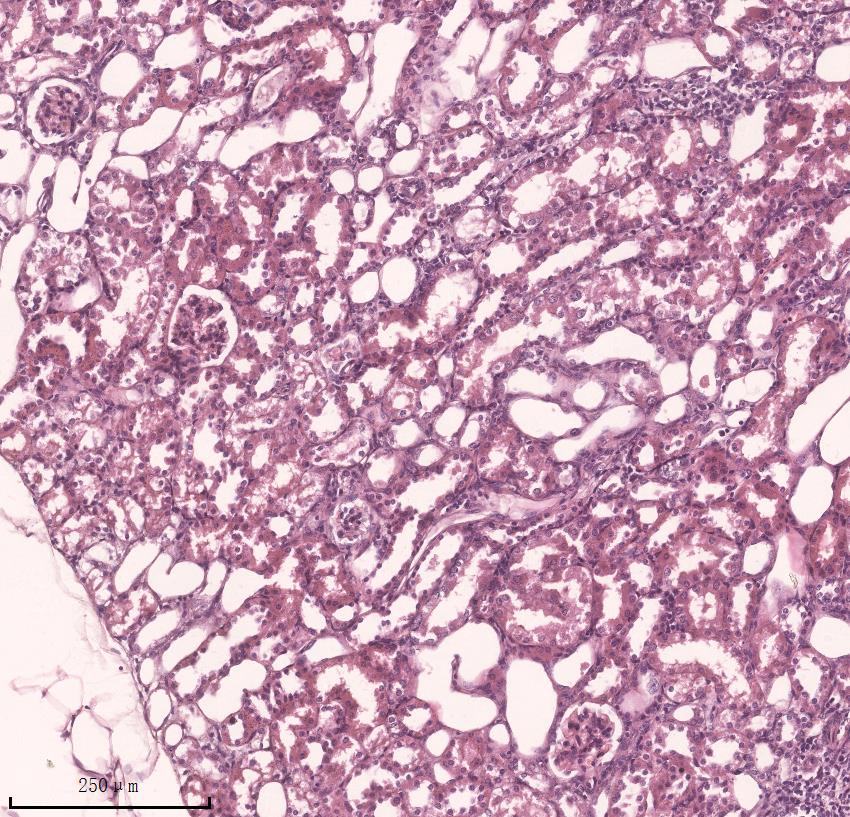

Supplement: Supplementary file 1 [file DataSheet1.zip › Original materials/Microscopic image/Fig 6/HE/AAN-4w 1.jpg]

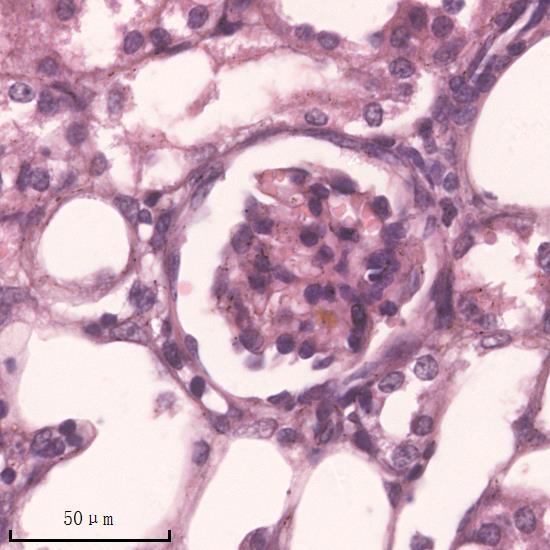

Supplement: Supplementary file 1 [file DataSheet1.zip › Original materials/Microscopic image/Fig 6/HE/AAN-4w 2.jpg]

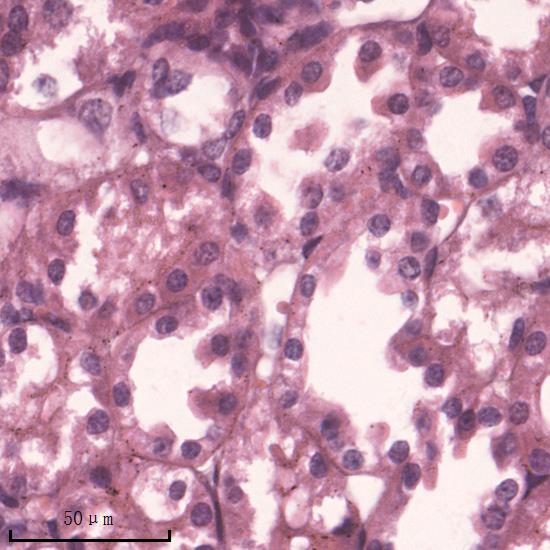

Supplement: Supplementary file 1 [file DataSheet1.zip › Original materials/Microscopic image/Fig 6/HE/AAN-4w 3.jpg]

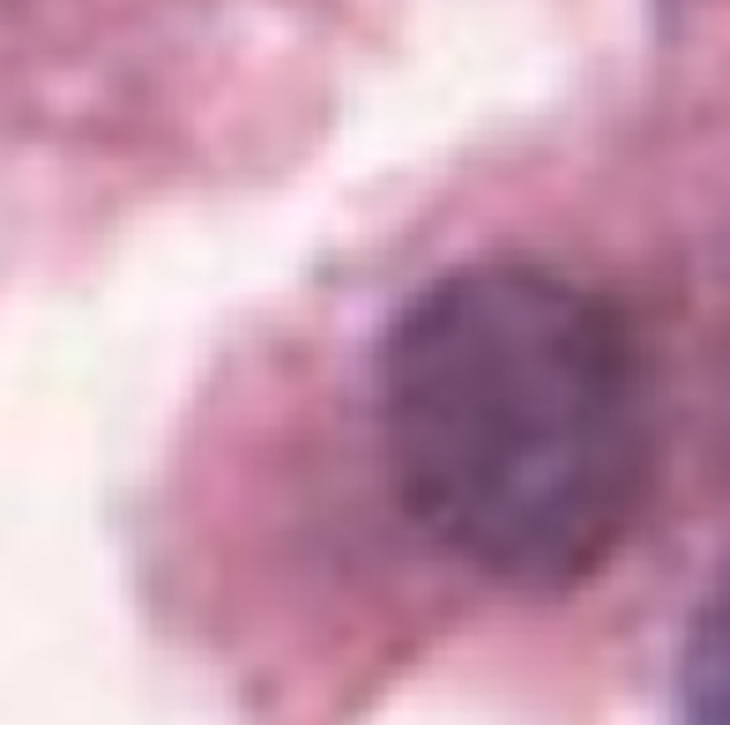

Supplement: Supplementary file 1 [file DataSheet1.zip › Original materials/Microscopic image/Fig 6/HE/AAN-4w 4.jpg]

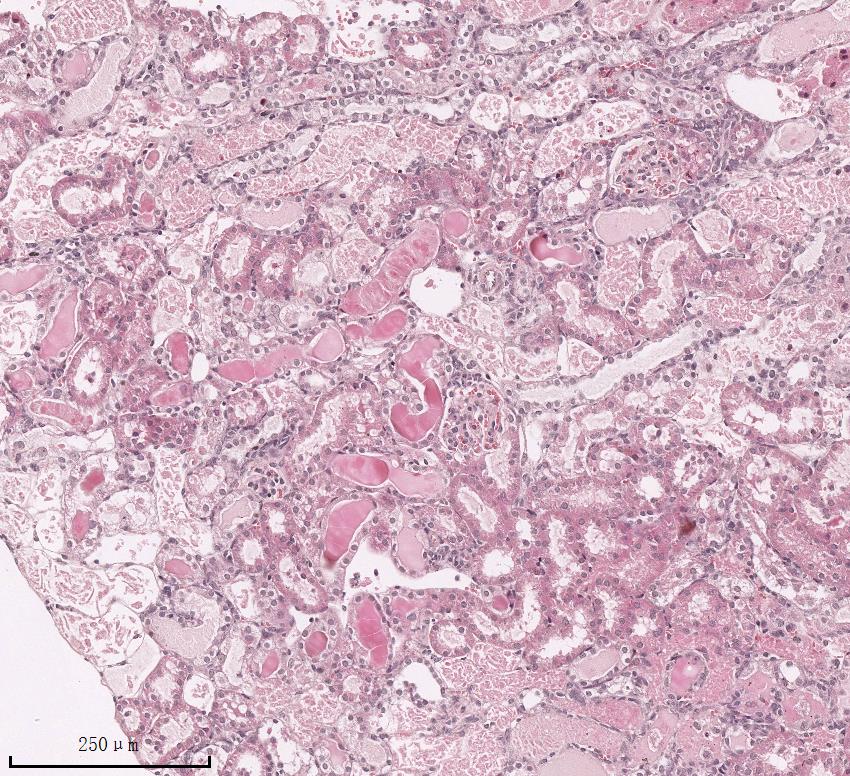

Supplement: Supplementary file 1 [file DataSheet1.zip › Original materials/Microscopic image/Fig 6/HE/AAN-8d 1.jpg]

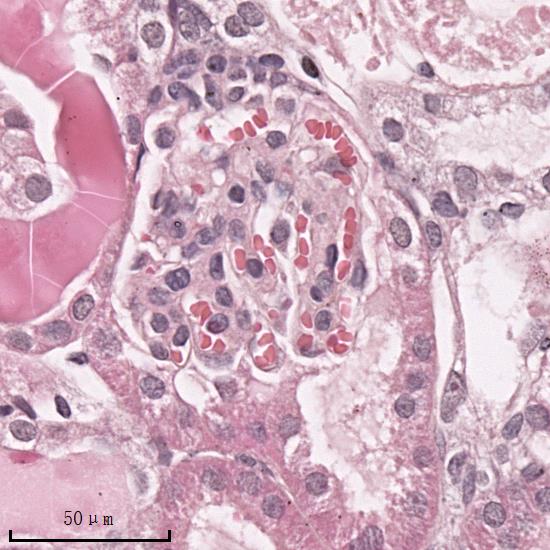

Supplement: Supplementary file 1 [file DataSheet1.zip › Original materials/Microscopic image/Fig 6/HE/AAN-8d 2.jpg]

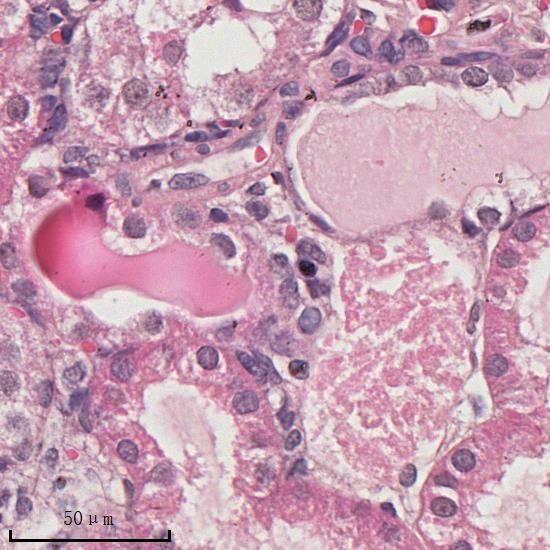

Supplement: Supplementary file 1 [file DataSheet1.zip › Original materials/Microscopic image/Fig 6/HE/AAN-8d 3.jpg]

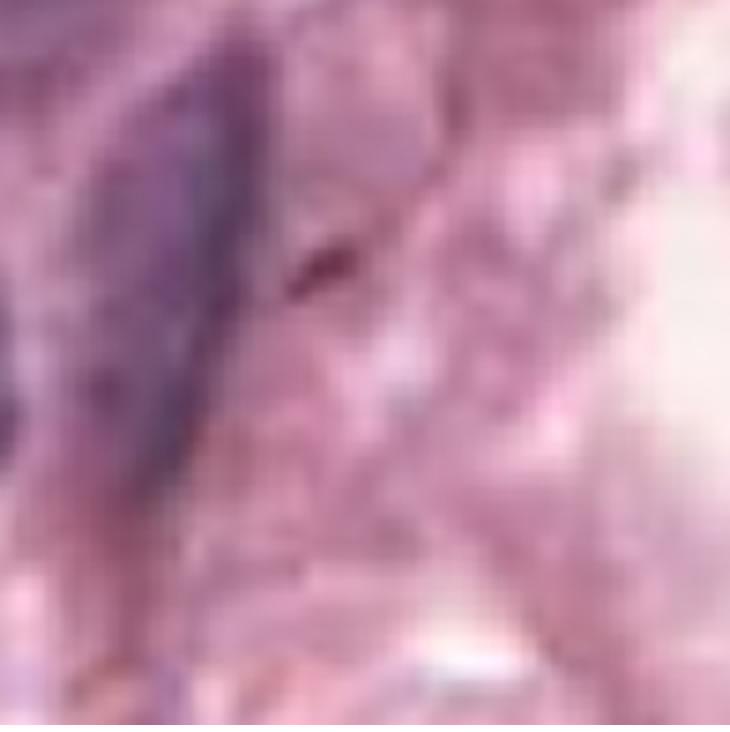

Supplement: Supplementary file 1 [file DataSheet1.zip › Original materials/Microscopic image/Fig 6/HE/AAN-8d 4.jpg]

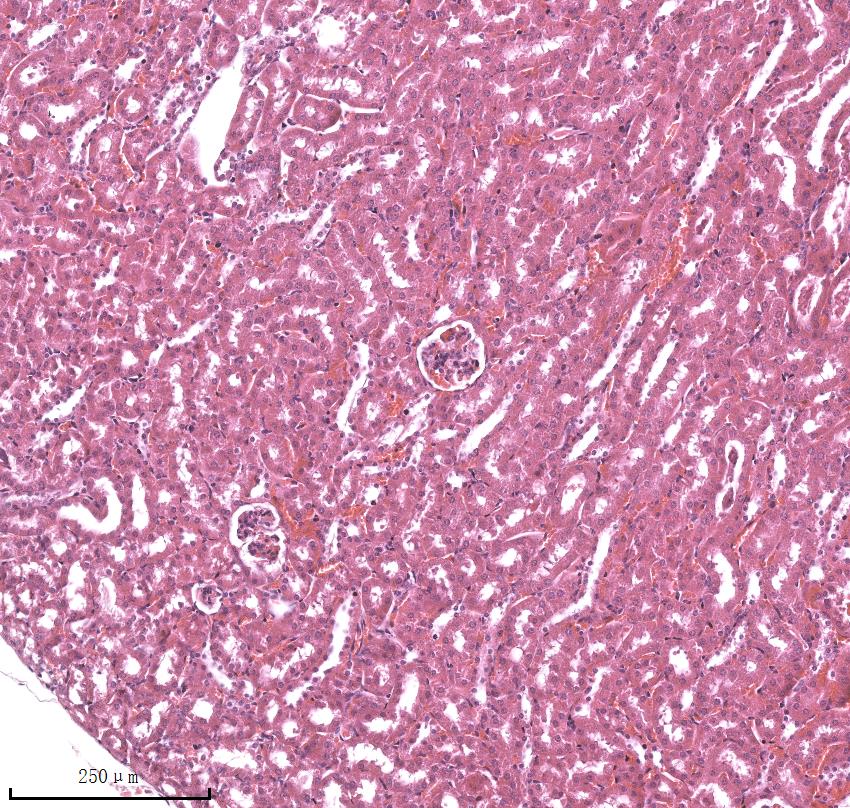

Supplement: Supplementary file 1 [file DataSheet1.zip › Original materials/Microscopic image/Fig 6/HE/AAN-Con 1.jpg]

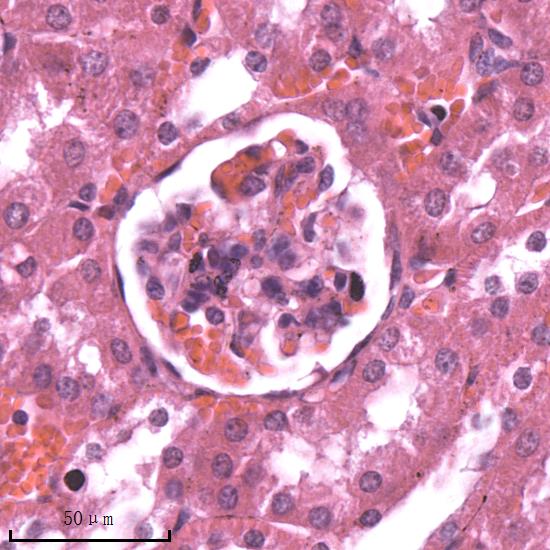

Supplement: Supplementary file 1 [file DataSheet1.zip › Original materials/Microscopic image/Fig 6/HE/AAN-Con 2.jpg]

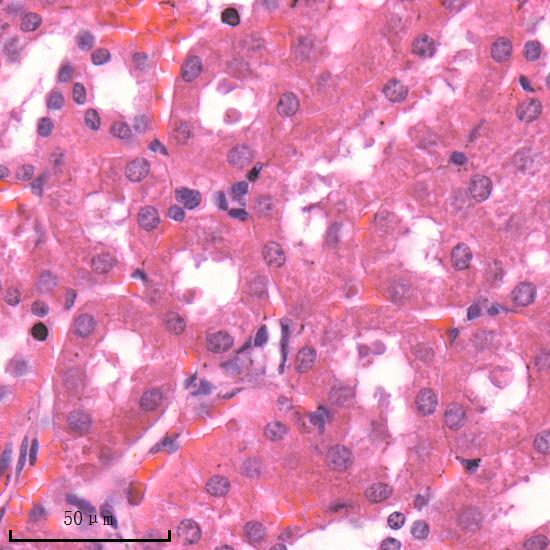

Supplement: Supplementary file 1 [file DataSheet1.zip › Original materials/Microscopic image/Fig 6/HE/AAN-Con 3.jpg]

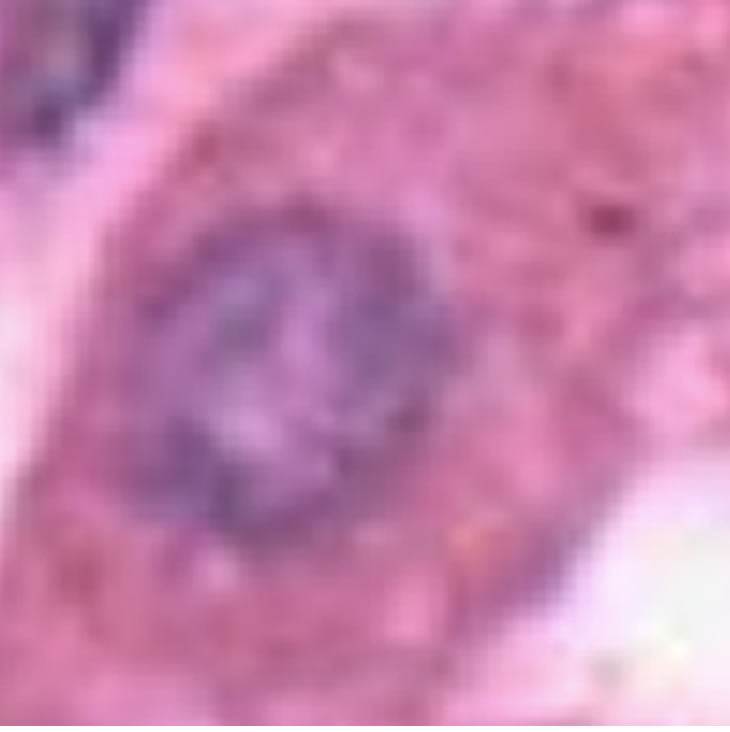

Supplement: Supplementary file 1 [file DataSheet1.zip › Original materials/Microscopic image/Fig 6/HE/AAN-Con 4.jpg]

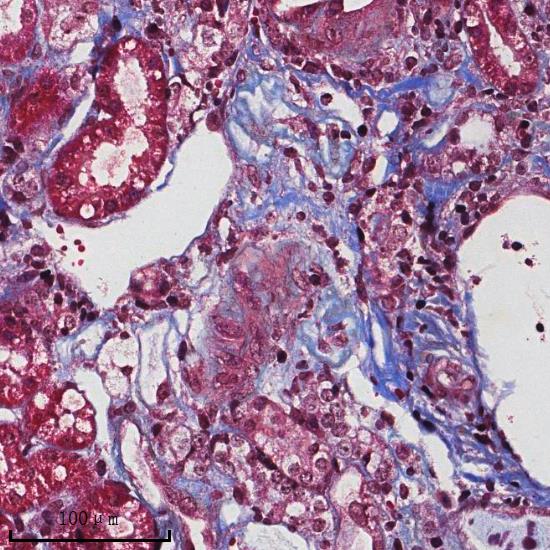

Supplement: Supplementary file 1 [file DataSheet1.zip › Original materials/Microscopic image/Fig 6/Masson/AAN-4w.jpg]

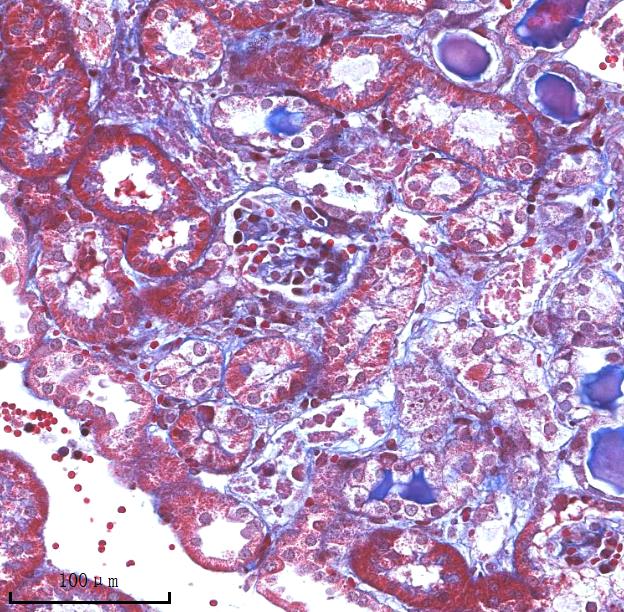

Supplement: Supplementary file 1 [file DataSheet1.zip › Original materials/Microscopic image/Fig 6/Masson/AAN-8d.jpg]

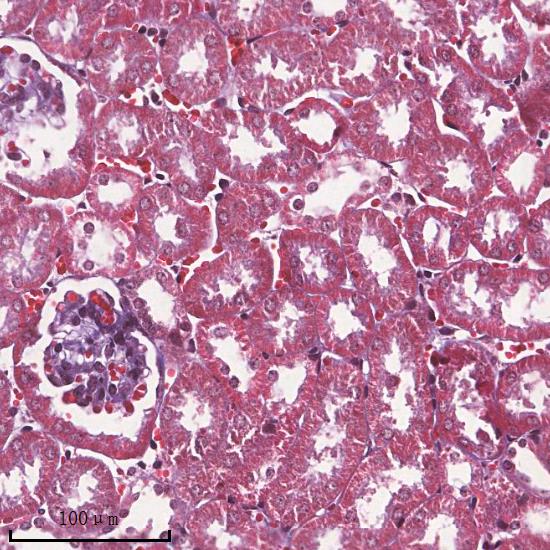

Supplement: Supplementary file 1 [file DataSheet1.zip › Original materials/Microscopic image/Fig 6/Masson/AAN-Con.jpg]

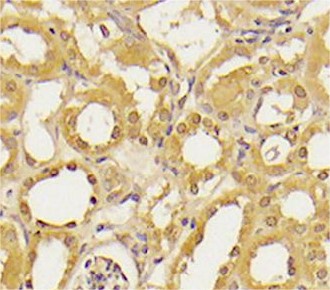

Supplement: Supplementary file 1 [file DataSheet1.zip › Original materials/Microscopic image/Fig 6/TOP2a/AAN-4w.jpg]

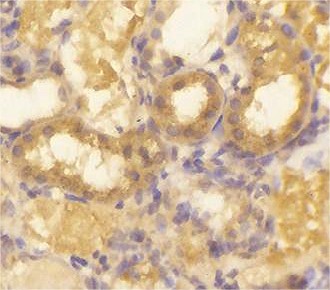

Supplement: Supplementary file 1 [file DataSheet1.zip › Original materials/Microscopic image/Fig 6/TOP2a/AAN-8d.jpg]

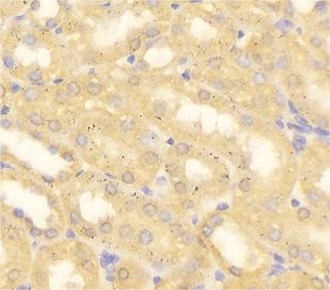

Supplement: Supplementary file 1 [file DataSheet1.zip › Original materials/Microscopic image/Fig 6/TOP2a/AAN-Con.jpg]

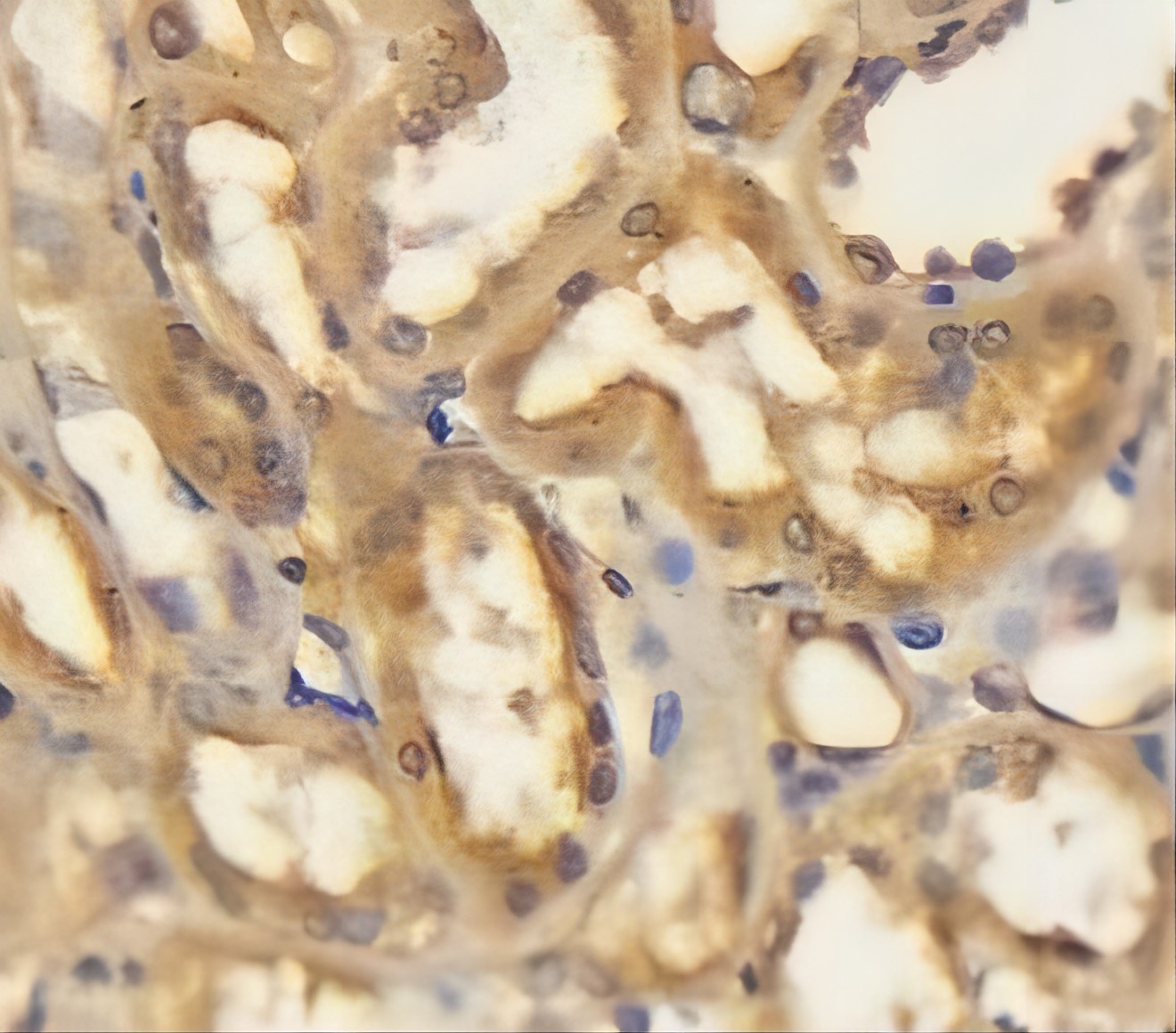

Supplement: Supplementary file 1 [file DataSheet1.zip › Original materials/Microscopic image/Fig 6/VCAM1/AAN-4w.jpg]

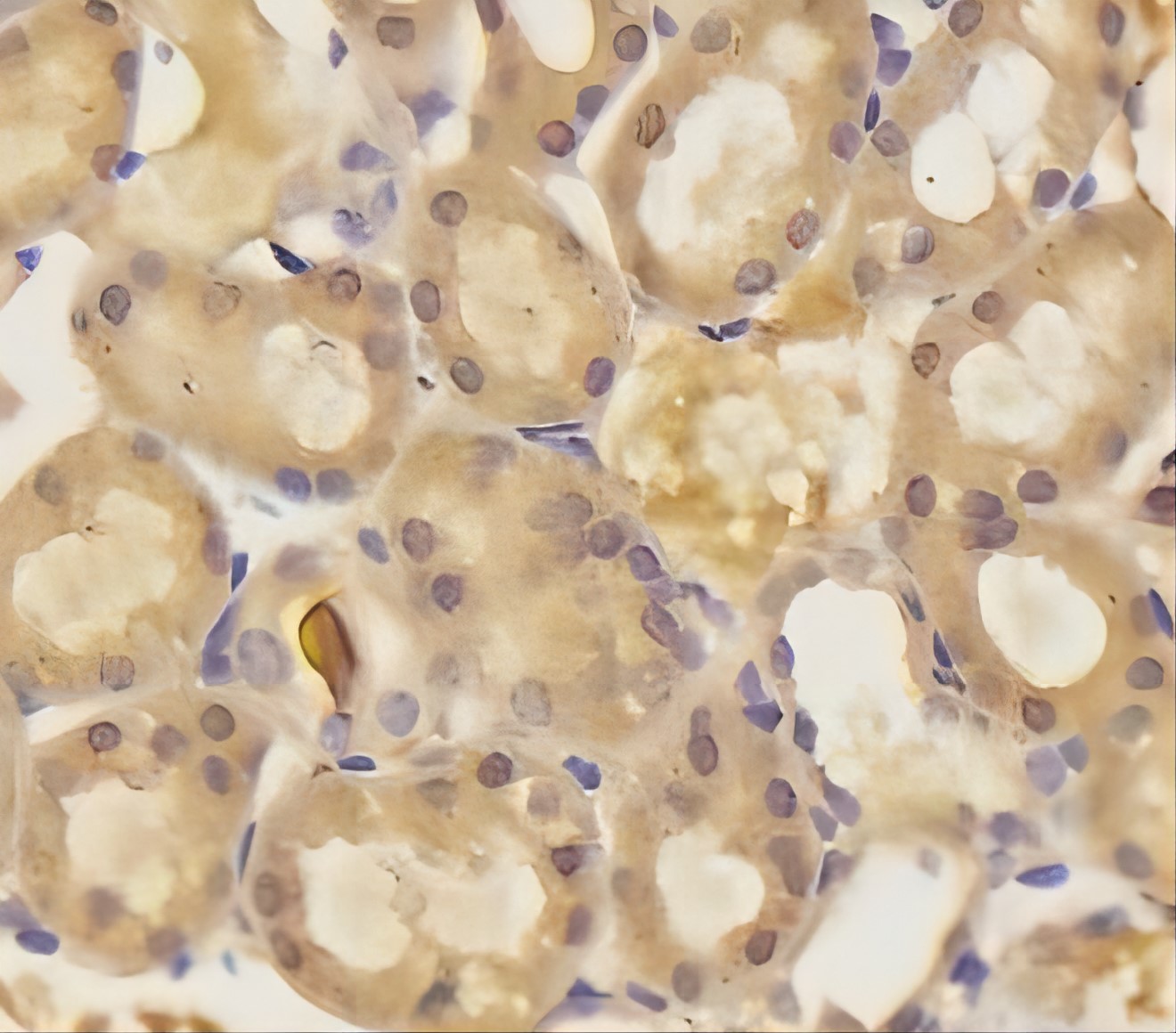

Supplement: Supplementary file 1 [file DataSheet1.zip › Original materials/Microscopic image/Fig 6/VCAM1/AAN-8d.jpg]

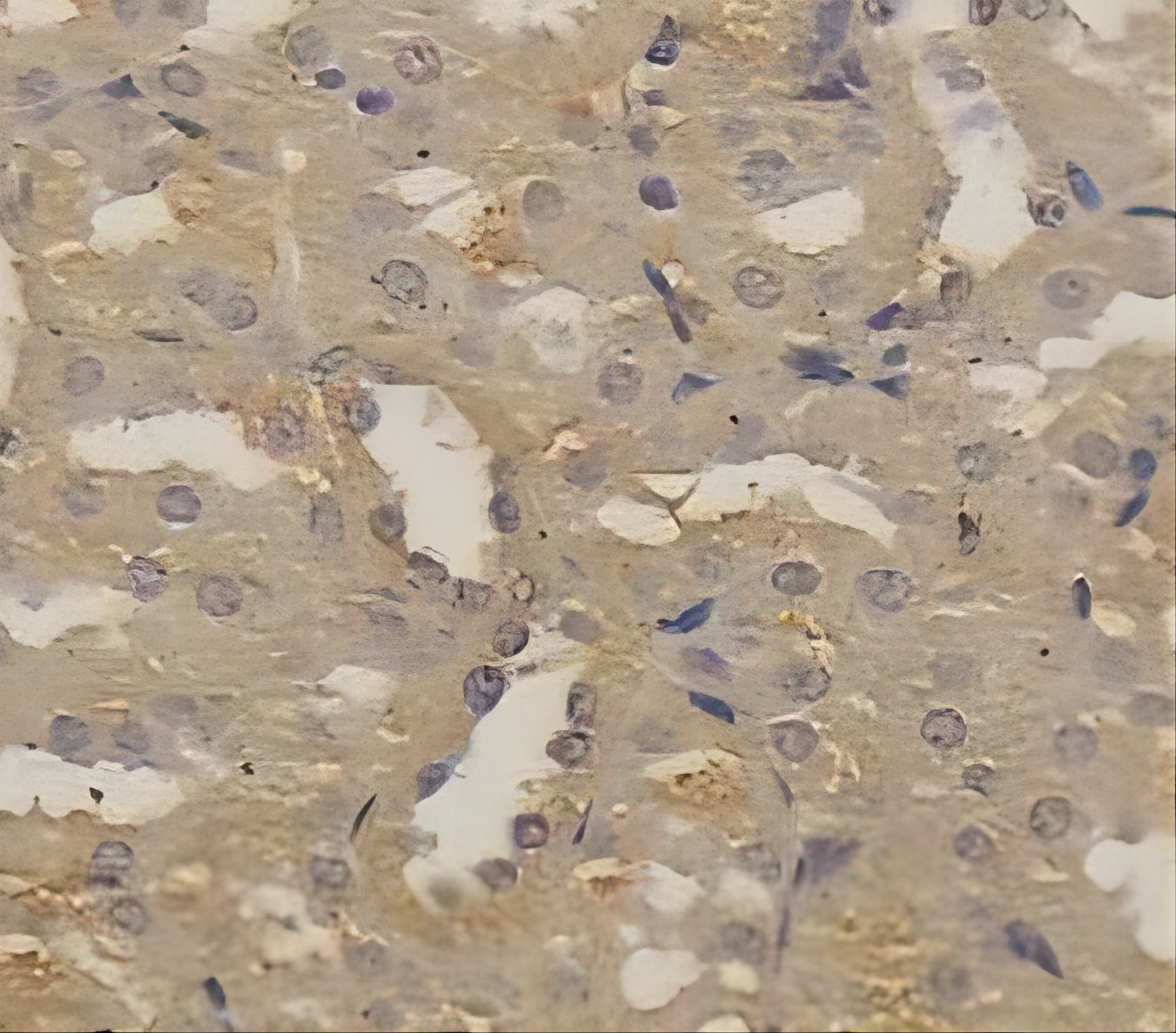

Supplement: Supplementary file 1 [file DataSheet1.zip › Original materials/Microscopic image/Fig 6/VCAM1/AAN-Con.jpg]

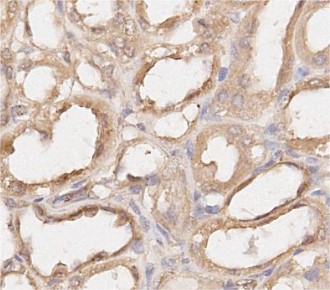

Supplement: Supplementary file 1 [file DataSheet1.zip › Original materials/Microscopic image/Fig 6/α-SMA/AAN-4w.jpg]

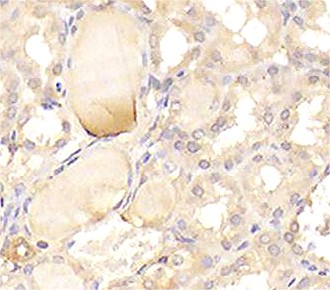

Supplement: Supplementary file 1 [file DataSheet1.zip › Original materials/Microscopic image/Fig 6/α-SMA/AAN-8d.jpg]

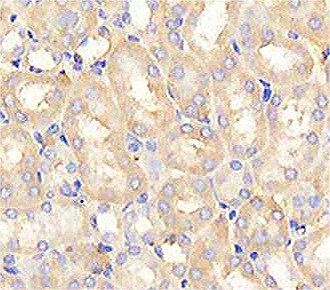

Supplement: Supplementary file 1 [file DataSheet1.zip › Original materials/Microscopic image/Fig 6/α-SMA/AAN-Con.jpg]
